# Genomic Analysis of Sequence-Dependent DNA Curvature in *Leishmania*

**DOI:** 10.1371/journal.pone.0063068

**Published:** 2013-04-30

**Authors:** Pablo Smircich, Diego Forteza, Najib M. El-Sayed, Beatriz Garat

**Affiliations:** 1 Laboratorio de Interacciones Moleculares, Facultad de Ciencias, Montevideo, Uruguay; 2 Departamento de Genética, Facultad de Medicina, Montevideo, Uruguay; 3 Department of Cell Biology and Molecular Genetics and Center for Bioinformatics and Computational Biology, University of Maryland College Park, Maryland, United States of America; NIGMS, NIH, United States of America

## Abstract

*Leishmania major* is a flagellated protozoan parasite of medical importance. Like other members of the Trypanosomatidae family, it possesses unique mechanisms of gene expression such as constitutive polycistronic transcription of directional gene clusters, gene amplification, mRNA *trans*-splicing, and extensive editing of mitochondrial transcripts. The molecular signals underlying most of these processes remain under investigation. In order to investigate the role of DNA secondary structure signals in gene expression, we carried out a genome-wide *in silico* analysis of the intrinsic DNA curvature. The *L. major* genome revealed a lower frequency of high intrinsic curvature regions as well as inter- and intra- chromosomal distribution heterogeneity, when compared to prokaryotic and eukaryotic organisms. Using a novel method aimed at detecting region-integrated intrinsic curvature (RIIC), high DNA curvature was found to be associated with regions implicated in transcription initiation. Those include divergent strand-switch regions between directional gene clusters and regions linked to markers of active transcription initiation such as acetylated H3 histone, TRF4 and SNAP50. These findings suggest a role for DNA curvature in transcription initiation in *Leishmania* supporting the relevance of DNA secondary structures signals.

## Introduction

*Leishmania* is a flagellated protozoan parasite (order Kinetoplastida) of significant medical importance in tropical and subtropical regions of the world. The parasite alternates between an intracellular amastigote form residing in vertebrate macrophages and an extracellular promastigote form living in the digestive tract of sandflies. The numerous human-infective *Leishmania* species cause a spectrum of diseases known as leishmaniasis, ranging from asymptomatic to lethal infection of internal organs. It has been estimated that more than two million new leishmaniasis cases occur each year and that 367 million people are at risk of infection [1].

*Leishmania* and other members of the Trypanosomatidae family possess unique mechanisms of gene expression, nonetheless, little is known about the nucleic acid signals driving them [2].

Although no precise element participating in chromosome replication, segregation and mitotic stability has been described in *Leishmania*, sequences with similarity to the yeast autonomously replicating consensus sequence and centromere DNA elements have been detected in *L. donovani* [3]. Functional characterization using unstable artificial chromosomes suggests the existence of multiple dispersed elements for mitotic stability in *L. major* [4]. Repeated sequences (direct or inverted) involved in DNA rearrangements, alteration of gene copy number (deletion or amplification), formation of extrachromosomal circular or linear amplicons and supernumerary chromosomes have been described in *Leishmania* [5], [6]. In addition, retroposon traces have been reported [7], [8], [9] and their involvement in mRNA instability and in the control of transcription initiation have been proposed [10].

Similar to other eukaryotes, transcription initiation by RNA polymerase I and III occurs at defined core promoters in trypanosomatids [11], [12], [13]. Canonical signals for RNA polymerase II recruitment have been characterized for the promoters of spliced leader, (SL) genes [14], [15], but not for the transcription of genes contained within the directional gene clusters (DGCs). Nuclear run-on assays performed in *L. major* indicate that RNA polymerase II transcription of genes initiates at the beginning of each divergent DGC [16], [17]. ChIP-chip assays in *L. major* revealed the enrichment of acetylated H3 histone at divergent strand switch regions (SSRs), as well as the increased binding frequency of two transcription factors –TRF4 and SNAP50. Interestingly, these features also occur at other specific regions putatively related to transcription initiation [18]. Although a high G+C content of SSRs has been observed, no signals such as TATA box or other typical RNA polymerase II core promoter elements have been detected [19]. Transcription termination signals have not been clearly defined for RNA polymerase II transcription of protein-coding genes, although a tract of Ts seems to be required in the case of the spliced leader genes in *Leishmania tarentolae* [20].The analysis of the regions between convergent DGCs, where termination signals may occur, reveals the presence of tRNA genes as well as other genes transcribed by RNA polymerase III [21], [22]. More recently, the presence of the modified base J has been observed at polimerase II transcription termination sites by chromatin immunoprecipitation studies. The results show the importance of this modified base in the transcription mechanisms of these parasites [23]. In the case of RNA polymerase I, regions with the ability to form stem-loop conformations reminiscent of the prokaryotic *rho*-independent termination have been described in *L. infantum* [24] and *L. major* [25]. RNA polymerase III termination in *L. major* occurs at T runs averaging 4.87 in length [21]. The maturation of individual trypanosomatid mRNAs derived from long nascent transcripts is marked by the *trans*-splicing of a mini-exon sequence at the 5′ end and polyadenylation of the 3′end of the processed mRNA [26]. While conserved eukaryotic splicing signals (AG dinucleotide at the 3′ splice site and upstream polypyrimidine tract) have been reported for the SL addition process, no specific consensus sequence or site selection mechanism have been identified for polyadenylation. Nevertheless, *trans*-splicing and polyadenylation of adjacent genes are coordinated [27]. Efforts to determine the *cis*-elements responsible for post-transcriptional regulation of gene expression have led to the identification of some sequence elements, secondary structures or a combination of both (mostly in the 3′ untranslated regions (UTRs)) [28], [29], [30], [31], [32], [33]. In summary, sequence elements regulating transcriptional and post-transcriptional processes, particularly for those genes transcribed by RNA polymerase II, remain largely unknown in *Leishmania.*

Difficulties in identifying DNA regulatory signals in trypanosomatids may be derived from the focus on primary structure analysis of DNA. Nevertheless, DNA conformations have been largely recognized as signals for regulation of DNA function. While evidence for the role of conformational signals in replication, DNA rearrangements and gene expression continues to accumulate in prokaryotic and eukaryotic cells [34], little is known about genomic DNA conformation in kinetoplastid parasites. Early work on *L. tarentolae* mitochondrial minicircle DNA showed the anomalous migration of restriction fragments due to the natural curvature of the DNA helix [35], [36]. More recently, bioinformatic analyses of some strand switch regions in *L. major* suggested a functional role for DNA secondary structures as replication or transcription boundaries [37]. A bias in nucleotide composition [18] and poly-dinucleotides abundance [38] has also been reported for those regions.

We have carried out a detailed genome-wide analysis of intrinsic curvature (IC) in *L. major* in order to explore the enormous potential of DNA regulatory signals. When compared to other organisms, the *L. major* genome revealed a lower frequency of high intrinsic curvature regions as well as inter- and intra- chromosomal distribution heterogeneity. Using a novel method aimed at detecting region-integrated intrinsic curvature (RIIC) and based on the additive contribution of IC along regions of a given length, we identified divergent SSRs as high scoring regions. Since those regions are thought to be implicated in transcription initiation, and assuming that curvature profiles provide a relevant signal, we used those RIIC characteristics to search the rest of the *L. major* genome for other regions with similar predicted curvature output. The identified regions matched regions within the DGCs which have been reported to be associated with markers of active transcription initiation (acetylated H3 histone, TRF4 and SNAP50) [18]. A high degree of conservation of the curvature locations was observed for the two *Leishmania* species studied, that is *L. major* and *L. infantum*.

## Materials and Methods

### Data Sources

The genome data for L. major, L. infantum, L. mexicana, and L. braziliensis, T. cruzi and T. brucei were downloaded from TritrypDB (version 2.1 for L. major, T. brucei and T. cruzi, version 3.1 for L. infantum, L. mexicana and L. braziliensis). A fragment of approx. 7 Mb (spanning bases 136,666,200 to 143,535,568) of human chromosome 1 (Chr 1) was obtained from the NCBI reference sequence (Build 37.2) and used as an external reference group. The E. coli BW2952 strain genome sequence was downloaded from NCBI (Acc. NC_012759.1) and used as a prokaryotic reference genome.

### Genome-wide Intrinsic Curvature Calculation

Individual chromosomes were split into 200 Kbases (Kb) non overlapping fragments and submitted for IC prediction using in-house scripts. The bend.it algorithm [39] was kindly provided by Dr. S. Pongor and was run locally. We used the default window size (31 bp) and bendability values (derived from nucleosome binding and DNaseI analysis) to estimate curvature. The output consisted of IC predicted angles per helical turn of the double helix (degrees/hel. turn). Results for all the fragments were parsed using in-house scripts and wiggle (WIG) files were generated for each chromosome in order to visualize the obtained results in genome browsers. Results were filtered and/or further analyzed using in-house scripts either in Python or in R programming languages [40].

### Curvature Analysis of Putative Transcription Start Sites

For the statistical analysis of the IC of regions of interest, the area under the curvature plot was calculated using the Riemann sum [41]. For a given region, the value of this region integrated intrinsic curvature (RIIC) parameter was compared to a density function representing the population of RIIC scores for equal-length regions in the genome. This probability density function was estimated by Gaussian kernel density estimation based on 5×10^3^ RIIC values for random windows in the parasite genome (excluding other regions of interest). A region was classified as highly curved if its RIIC score was greater than the 95% confidence interval calculated using the estimated density function (p<0.05) (for a graphical representation see Supplementary Figure 1). SSRs were selected for RIIC analysis. The coordinates for all the SSRs were collected using in-house perl scripts and manually curated. For each analysis, the region length was defined based on the distance between the adjacent DGCs. In addition, SSRs were considered as convergent (CSSR) or divergent (DSSR) depending on the orientation of the flanking DGCs. The number of SSRs that could be classified as highly curved regions was counted. The likelihood of counting a given number of highly curved regions was evaluated using the exact binomial test considering 0.5 as the expected probability of random category assignment.

**Figure 1:**
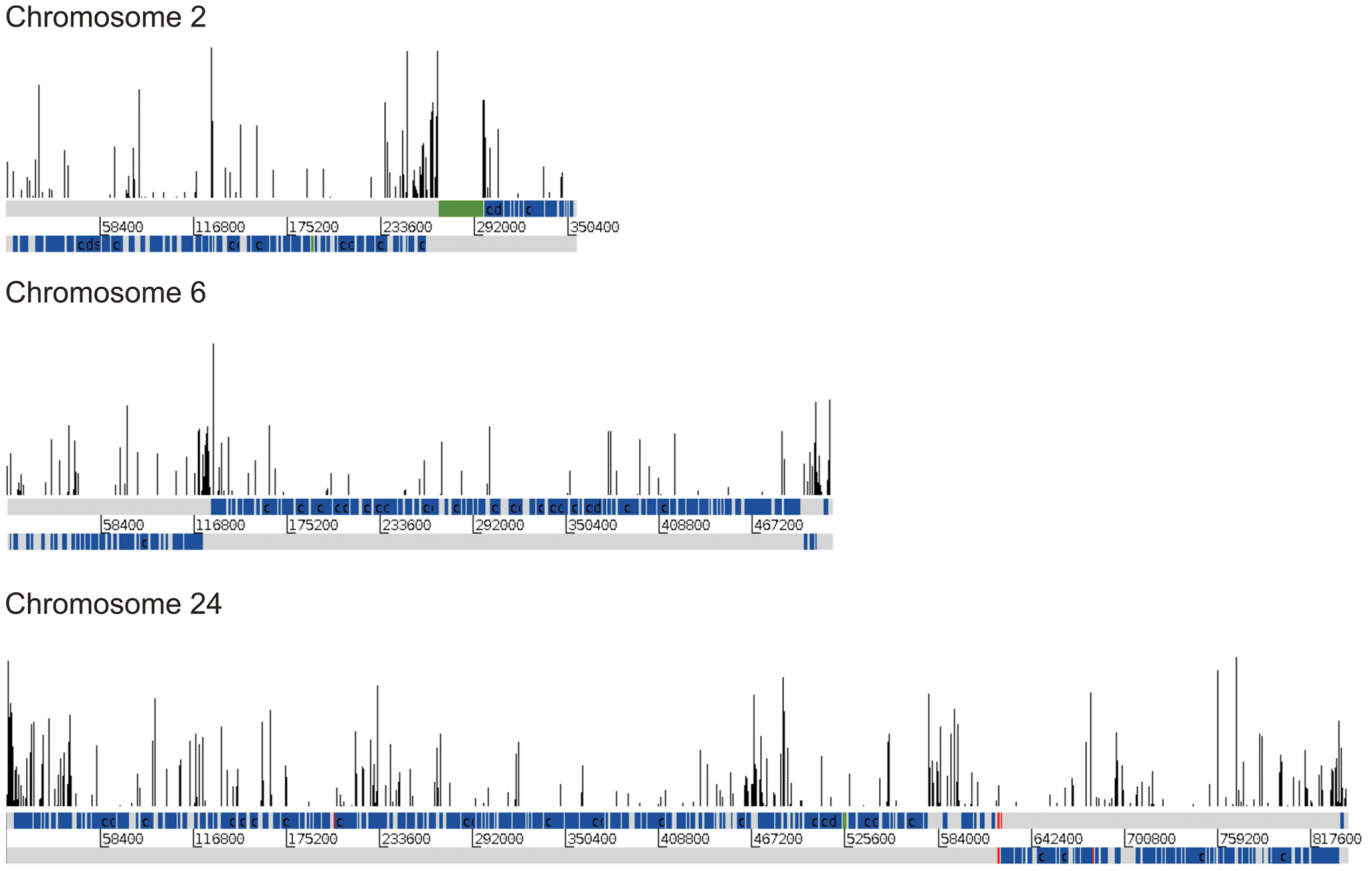
Graphical representation of IC peaks on selected *L.* *major* chromosomes. Bar plots of IC positions with an IC value greater than 9 degrees per helical turn. Both DNA strands are depicted in grey below bar plots, overlaid with CDS features shown in blue. Features labeled as ncRNA, snRNA or snoRNAs are shown in green. tRNAs are shown in red.

### Genome-wide Search for High RIIC Locations and Synteny

In order to predict regions of high curvature and reduce background signal generated by isolated high IC peaks, R language scripts were written to search every chromosome for 600 bp regions with a RIIC greater than the 85^th^ percentile value for that chromosome. The number of regions of high RIIC was computed and their location compared to the reported for TSS markers. A contingency table was built, using experimental data as the positive reference, and the Matthews correlation coefficient was calculated to determine the plausibility of using the RIIC score to predict the presence of TSS markers.

Syntenic regions were evaluated with the Artemis Comparison Tool (ACT) tool [42]. Chromosome-wide alignments were performed with BLASTN [43].

## Results

### Intrinsic Curvature Distribution in the *L. major* Genome

In order to characterize the secondary structure of the *Leishmania* genomic sequences, an analysis of the intrinsic curvature distribution was carried out using the bend.it algorithm. For comparison purposes, the profile obtained for a similarly sized fragment of the human chromosome 1 and the *E. coli* genome were included. All sequences assayed showed a non-symmetrical random distribution of IC (Supplementary Figure 2), a result consistent with reports for other organisms [44]. Remarkably, a clear shift towards lower values of IC was observed in *L. major*. Although less evident, a similar shift was also observed for the two other trypanosomatids, *T. brucei* and *T. cruzi*. Very similar and indistinguishable profiles were observed for all the different *Leishmania* species with available genomes (*L. major*, *L. infantum*, *L. braziliensis*, *L. mexicana*) (data not shown), probably due to their significant sequence homology [45]. In comparison to other organisms, ranging from prokaryotes to humans, including trypanosomes, the *Leishmania* IC profile showed fewer regions with high curvature and a sharper peak corresponding to a higher density of regions with lower curvature. Because of this peculiarity, we focused on the analysis of curvature distribution in *Leishmania*.

**Figure 2:**
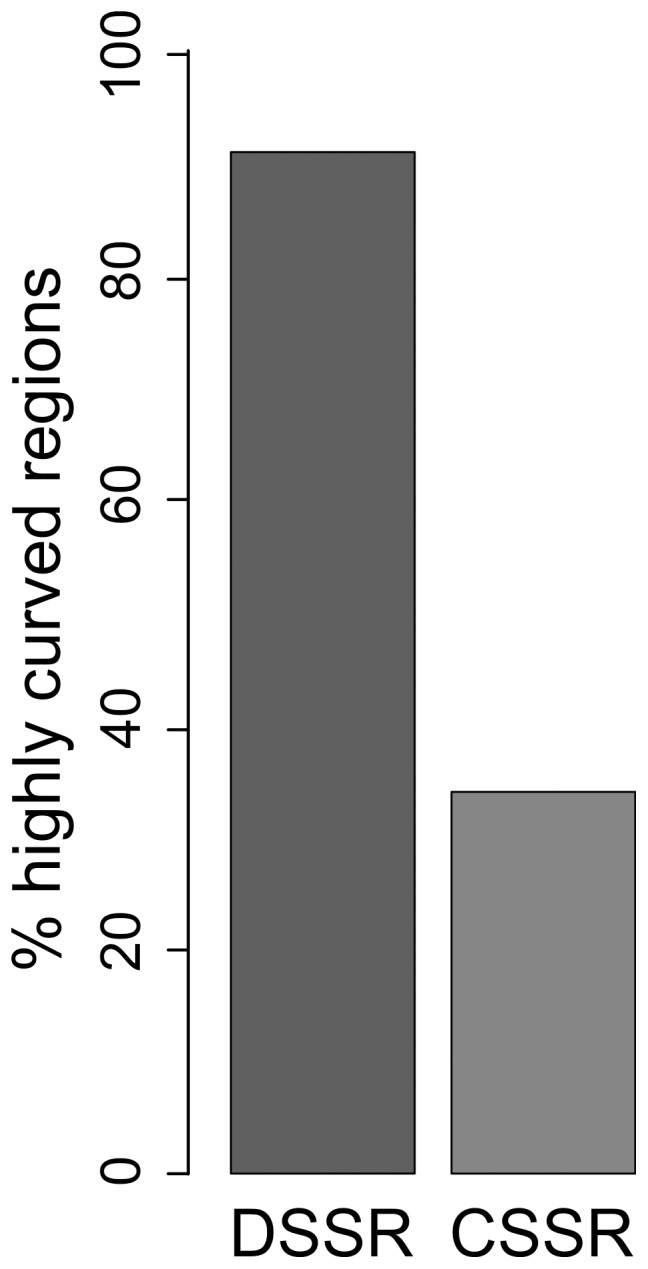
Regional Integrated Intrinsic Curvature analysis in *L.* *major*. Bar plot showing the percentage of regions with significant RIIC score (p<0.05), indicated as highly curved, for divergent (DSSR) and convergent (CSSR) strand switch regions.

No significant differences in IC values were observed among the *L. major* chromosomes, with medians ranging from 2.37 (Chr 1) to 2.78 (Chr 36) degrees per helical turn. A similar profile was observed also for *L. infantum*, *L. braziliensis* and *L. mexicana*. Interestingly, a slight increase of the IC medians accompanying chromosome lengths could be noted in all the *Leishmania* species analyzed (Supplementary Figure 3). Indeed, when only the number of peaks with high IC (≥9 degrees/hel. turn) was plotted, a non-linear increase was observed with augmenting chromosome length. Accordingly, the average distance between high IC peaks (density of high peaks) changes from a peak every 450 bp for the smaller chromosomes to a peak every 150 bp for the larger ones (Supplementary Figure 4). In contrast to various organisms where the mean values of curvature were previously shown not to be related to the G+C genome content [44], we found the IC medians to correlate inversely with G+C content within the Tritryp genomes (Supplementary Table 1) and *Leishmania* chromosomes (Supplementary Table 2), (R^2^ = 0.8941).

**Figure 3:**
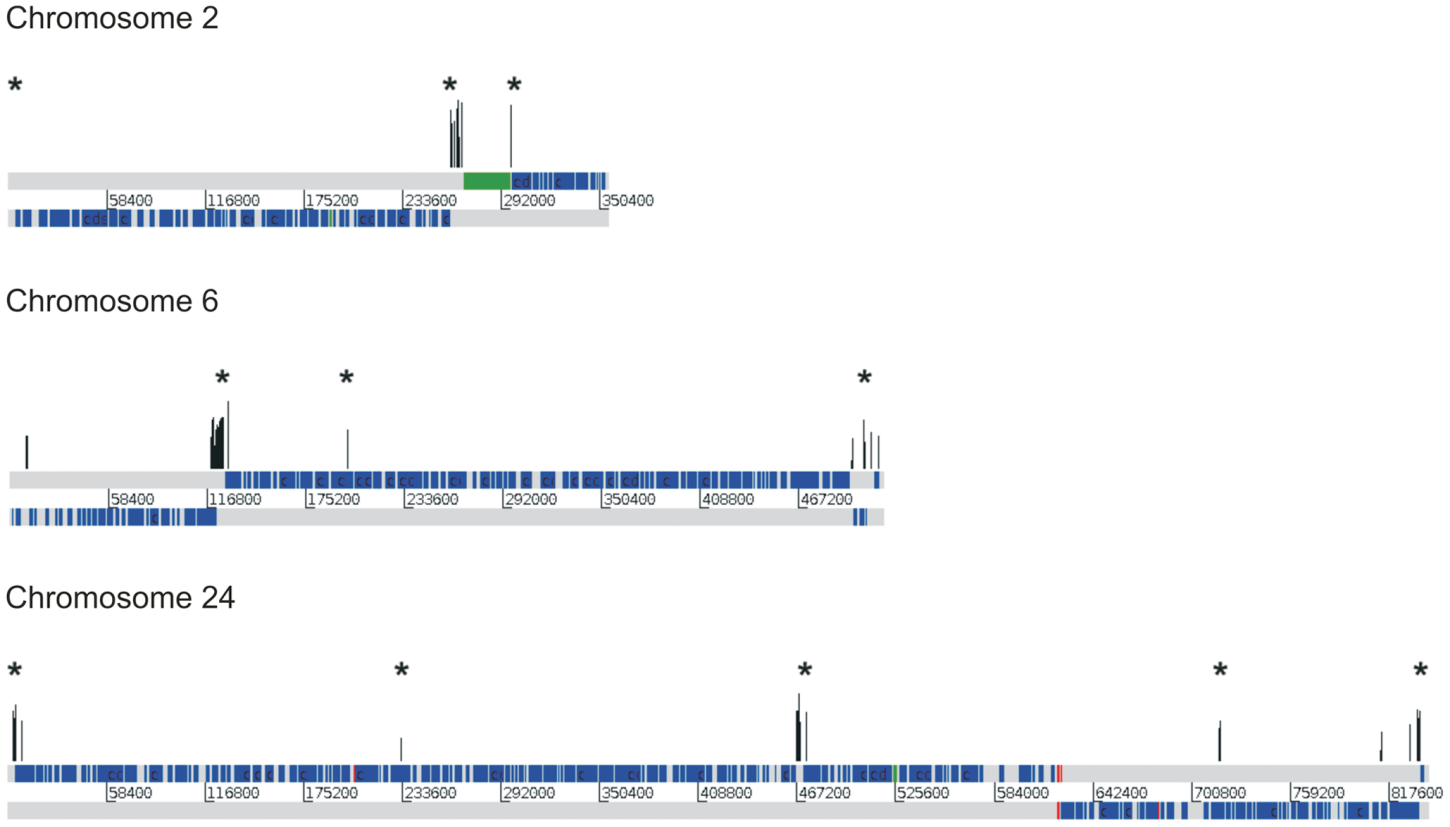
Graphical representation of IC for regions with high RIIC score for three *L.* *major* chromosomes. The graphs are the same as figure 1. IC for regions with high RIIC score are represented on top. Asterisks mark sites defined as sites associated with acetylated H3 histone [18].

**Figure 4:**
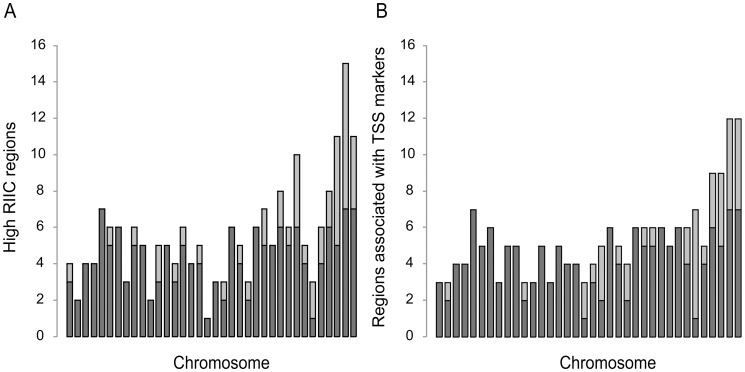
Comparative analysis of high RIIC regions *vs.* reported putative TSS for each *L.* *major* chromosome. **A**. The total number of high RIIC regions for each *L. major* chromosome, depicted in ascending order from left to right, is represented. Dark grey indicates high RIIC scoring regions that overlap with regions associated with TSS markers reported by Thomas *et al.* [18]. **B**. The total number of regions associated with TSS markers obtained in Thomas, *et al*. [18] for each *L. major* chromosome, depicted in ascending order from left to right, is represented. Dark grey indicates the number of regions associated with TSS markers that overlap high RIIC regions.

To further evaluate a potential functional role for sequence-dependent DNA curvature in specific chromosomal locations, we analyzed the location and context of peaks of high intrinsic curvature along each *L. major* chromosome. Representative profiles of some chromosomes are shown in Figure 1 (see Supplementary Figure 5 for IC profiles of all *L. major* chromosomes). To reduce the background, only IC peaks above 9 degrees per helical turn were considered. For short chromosomes, such as Chr 2 or Chr 6, a high density and/or intensity of IC regions can be observed close to the SSRs. At such regions, high AT content [37] and poly-dinucleotide abundance [38] have been described. Using a similar approach and the bend.it algorithm, Tosato *et al.* [37] reported the presence of peaks of high DNA curvature at SSRs after the analysis of the available chromosome data of *L. major* at the time (complete sequences of Chr 1 and 3 and partial sequences of Chrs 4, 19 and 21). While we make the same observation genome-wide, we could not assert a specific correlation between high intrinsic curvature and SSR due to the high noise levels (see for example Chr24). In addition, the absence of curvature enrichment at the convergent SSRs can be noted (see for example Chr24). These results prompted us to search for more stringent conditions or approaches for the analysis of IC profiles.

**Figure 5:**
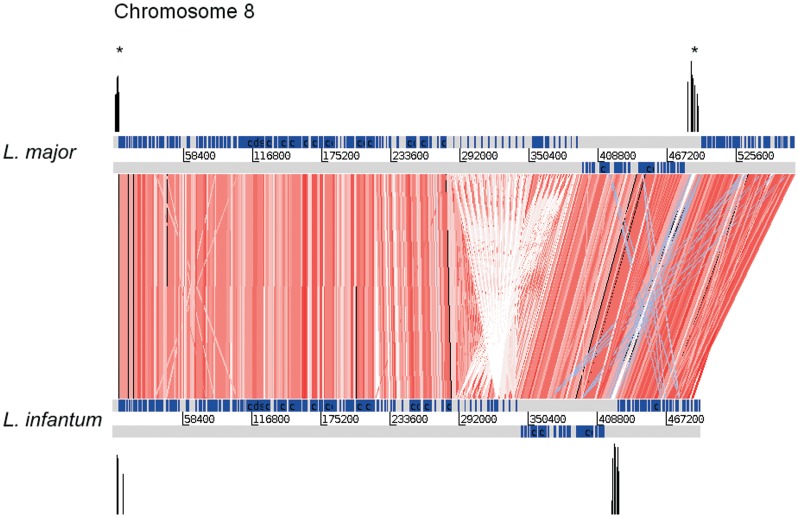
Location conservation of high RIIC scoring regions in *L.* *major* and *L. infantum* chromosome 8. The graphs are the same as figure 1. Blast HSPs longer than 100 bp and with at least 80% similarity are displayed in red scale and blue lines represent inversions.

### Regional Integrated Intrinsic Curvature: a New Tool for Curvature Analysis in *L. major* Chromosomes

Previous studies aiming at detecting conformational signals associated with gene regulation have generally restricted analyses to the occurrence of IC peaks with values above 15 degrees per helical turn for defined short segments with length ranging from 31 to 51 bp [44]. We were not able to make functional associations for regions characterized by high IC peaks in the *L. major* genome. In an effort to use a more sensitive tool, we developed an alternative approach based on the analysis of the area for a selected region in the curvature graph. This approach takes into account the fact that conformational signals likely derive from a combination of the effects of both frequency and intensity of IC in a given region. The scoring function was named RIIC for regional integrated intrinsic curvature.

In order to test the ability of RIIC scores to distinguish functional regions, we applied a statistical test comparing each of 98 selected regions (corresponding to 58 DSSRs and 40 CSSRs) to 5,000 control genomic regions of the same length (see Materials and Methods). A clear association of RIIC scores with divergent SSRs was found (Figure 2). Indeed, 93% of the DSSRs were found to present significantly high RIIC scores compared to random sequences (p<0.05), contrasting with only 33% of the CSSRs. A binomial exact test was performed to evaluate the significance of this observation, confirming that the method could clearly differentiate the DSSR as highly curved regions (p<10^−12^). Divergent SSRs have been proposed as sites for transcription initiation in kinetoplastids [16], [17]. This is consistent with some early work devoted to the global analysis of the association of intrinsic curvature with transcription initiation where *E. coli* promoter regions were shown to be more curved than coding sequences. Sequence-dependent DNA curvature is known to play an important role in the transcription initiation of many specific genes both in prokaryotes and eukaryotes [46]. It is therefore plausible to propose that *Leishmania* conformational curvature is associated with transcription initiation.

### Location of Peaks of High RIIC Score within *L. major* Chromosomes

Since we were able to detect high RIIC scores in *L. major* divergent SSR and considering that distinguishable curvatures may constitute a signal involved in transcription initiation, we investigated the genome-wide occurrence of high RIIC scores. For that purpose, chromosomes were scanned for regions of high RIIC. Regions of 600 bp with RIIC scores higher than the 85^th^ percentile for every particular chromosome were mapped. This approach enabled the detection of intra-DGC regions that scored as high as the distinctive DSSRs. Representative profiles of some chromosomes are shown in Figure 3 (See Supplementary Figure 6 for high RIIC profiles of all *L. major* chromosomes). Interestingly, high RIIC regions are associated with markers of active transcription initiation (acetylated H3 histone, TRF4 and SNAP50) described in [18] (indicated as asterisks in Figure 3). These three markers were reported to co-localize in the *L. major* genome with only a few exceptions. For each chromosome, a summary of the association of the *L. major* genome high RIIC regions with the location of transcription initiation markers reported by Thomas *et al.* [18] is shown in Figure 4. We identified a total of 200 regions in the *L. major* genome with high RIIC, 155 (78%) of which coincide with the location of transcription initiation markers. Among those, 148 regions co-localize with acetylated H3 histone, five co-localize only with the TF4 marker and the other two are upstream ncRNA (Figure 4A). Conversely, out of a total of 191 regions associated with TSS markers, 153 (80%) had a high RIIC score (Figure 4B). A Matthews correlation coefficient of 0.78 supports the specific association between regions of high RIIC and putative TSS. Further work would be necessary to assess if the regions characterized by high RIIC score and currently not associated with TSS markers are actually involved in *Leishmania* transcription initiation.

**Figure 6:**
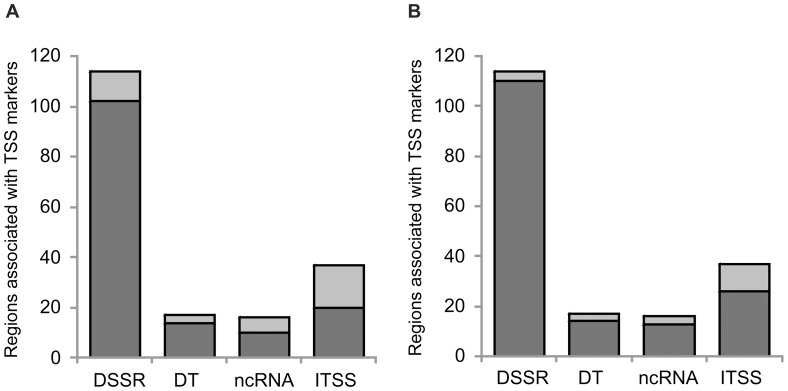
Comparative analysis of high RIIC regions *vs.* reported putative TSS in *Leishmania*. **A**. The total number of different type of regions (D SS: Divergent Strand Switch; D T: divergent telomeres; ncRNA: associated with ncRNA transcription; I TSS: internal to polycistrons TSS) associated with TSS markers obtained in Thomas, *et. al*. [18] in the *L. major* genome is represented. Dark grey indicates the number of regions associated with TSS markers that overlap with high RIIC regions. **B**. Same as in A displaying in dark grey the number of regions associated with TSS markers that overlap with high RIIC regions that are present either in *L. major* or *L. infantum*.

### Location Conservation of High RIIC Scores between *L. major* and *L. infantum*

The high association of DSSRs with curvature region is well conserved when *L. major* and *L. infantum* are compared. The Chr 8 profile is shown in Figure 5 (See Supplementary Figure 7 for representations of all chromosomes). Two regions of high RIIC score associated with conserved DSSRs are present in both *L. major* and *L. infantum* homolog chromosomes.

The existence of conserved physical co-localization of genes among *Leishmanias* [45] and also in the Tritryps [47] has been reported. However no remarkable intergenic sequence conservation has been observed. Interestingly, in many cases the location of high RIIC score regions is conserved in *L. major* and *L. infantum* in spite of the absence of sequence conservation (see for example the DSSR in Figure 5). The extension of synteny to high scoring RIIC regions, here presented, strengthens their functional significance. Globally, we found that from the 200 regions of high RIIC score identified in *L. major*, 166 are conserved in *L. infantum* and 145 of them coincide with regions associated to TSS markers (87%). These results clearly suggest that the conformational signal implication in transcription initiation observed in *L. major* can be generalized to the other *Leishmania* species. For the other 21 regions with high RIIC score, which are conserved in both species, no data about association with transcription initiation or any other functional role has yet been reported. The functional relevance of this finding is worth to be further investigated.

Considering that the analysis of the conservation of the location of high RIIC scoring regions may contribute to the understanding of the role of DNA intrinsic conformational signals in transcription initiation in *Leishmania*, a detailed comparative analysis was performed between *L. major* and *L. infantum*. The search for regions of high RIIC score in *L. major* allowed the identification of 102 out of a total of the 114 DSSRs associated to the TSS markers described by Thomas [18], representing an 89% coincidence (Figure 6A). Considering the addition of regions of high RIIC score from the *L. infantum* data, the number of regions associated to the TSS markers that match with high RIIC increases to 110 (96%)(Figure 6B). An improvement is also observed when considering regions associated with TSS markers inside DGCs (20 for *L. major* and 26 for *L. major* plus *L. infantum* out of 37 associated to TSS markers in [18]) and of non coding RNAs (10 for *L. major* and 13 for *L. major* plus *L. infantum* out of 16 associated to TSS markers in [18]).

## Discussion

Following the completion of the genome sequencing of the Tritryps (the three related trypanosomatid parasites: *T. brucei, T. cruzi*, and *L. major*) [47], [48], [49], genome wide approaches in *L. major* have been mainly aimed at the characterization of global gene and protein expression profiles during development and in response to drugs, as well as protein subcellular localization, and host-parasite interactions [18], [50], [51], [52], [53], [54], [55], [56], [57], [58], [59]. While nucleic acid signals acting on different steps of the gene expression process have been described, search efforts have focused on primary sequence signals and no elements involved in transcription initiation of protein coding genes have been found [2]. Nonetheless, the role of the nucleic acid conformation in molecular signaling has been largely recognized in prokaryotic and eukaryotic organisms.

The report of high IC at SSRs of some chromosomes in *L. major* over a decade ago, led to the speculation of their potential involvement at transcription or replication boundaries [37]. Using a similar approach and the bend.it algorithm, we extended the IC analysis to the entire genome. We found that *Leishmania* genomes characteristically present a high density of regions of low IC and relatively few regions of high IC. This characteristic markedly distinguishes the genome of these parasites from both the human chromosome 1 and the *E. coli* genome examined using the same tool. This distinctiveness is also evident even considering the two other Tritryp organisms. The segregation of *Leishmania* canonical behavior is not restricted to the IC density profile. *L. major* base skew pattern also differ from bacteria and even from those of *T. brucei* and *T. cruzi* [60], [61]. Though the absence of relation between G+C content and mean IC genome values has been reported [44], we found an inverse correlation within Leishmania chromosomes. Higher A+T content could increase the occurrence of A tracts that may induce DNA bends [62]. However, different sequential base orders, with variable G+C content, can also provoke DNA curvature. It has been recognized that SSRs in *L. major* are A+T rich [37], however we observe a clear difference of IC between convergent and divergent SSRs, showing that A+T content is not the only determinant of the presence of highly curved regions in DSSRs.

Taking into account the peculiar high abundance of low IC regions, we hypothesized the existence of conformational signals derived from the net effect of IC accumulation. In order to test our hypothesis, we developed a new approach using an integrative analysis of IC within a defined length region, allowing us to examine, for the first time, regions presenting a high integrated intrinsic curvature value. This approach revealed that high RIIC score regions are associated with DSSRs as well as to genome positions associated to TSS markers in *L. major*. These results support the existence of conformational signals involved in the definition of the transcription start points in *Leishmania.* It is worthwhile mentioning that a few exceptions were observed. Conversely, we also detected few regions characterized by high RIIC scores that have not been associated to TSS markers. Such regions may play a role in transcription initiation and therefore constitute interesting candidates for further investigation. Indeed, the recruitment of TSS markers to different regions may vary during cell cycle, following nutrient or heat shock and other challenges to the cell, establishing different DGCs and messenger abundance [18].

The locations of the regions with high RIIC scores appeared to be as well conserved as the gene synteny observed between *L. major* and *L. infantum.* The relevance of the conformational signal location is further outlined by the fact that this phenomenon is observed independently of sequence conservation.

It would be interesting to investigate the molecular mechanism triggered by the curvature conformation signals. Steps such as facilitated access to transcription initiation machinery directly or through accessory binding of proteins or enhanced melting of the DNA strands to assist active complex formation, may be involved. Bents, as well as other alternative DNA structures, have been involved in the regulation of transcription initiation both in prokaryotes and eukaryotes. Intrinsically bent DNA may specifically influence either chromatin folding, the binding of factors involved in basal transcription initiation and/or regulatory factors that interact with the transcription machinery. In eukaryotes, DNA curvature has been proposed as a primary nucleosome positioning signal [63], [64], and low nucleosome occupancy is considered a significant feature for the binding of transcription factors [65]. In addition, it has been reported that the transcription initiation of RNA polymerase II, whose localization depends on primary sequence signals, may be facilitated by the presence and orientation of curved DNA relative to the promoter [66]. Furthermore, for transcription initiation of RNA polymerase I, that is characterized by the absence of primary sequence signals, conserved conformational signals surrounding the transcription start point have been described in eukaryotes [67].

In addition, it would be interesting to investigate whether the association of high RIIC scores with TSS markers is a peculiarity of *Leishmania* genome or eventually and/or partially conserved in related organisms. The characterization of IC genome location for the two other TriTryps, *T. cruzi* and *T. brucei*, is in progress. Nevertheless, this first genome-wide analysis allowed us to clearly identify distinct regions of intrinsic DNA curvature and to associate them with a biological function in *Leishmania*, strongly linking DNA conformational signals with transcription initiation.

## Supporting Information

Figure S1 **Regional Integrated Intrinsic Curvature analysis in** ***L. major.*** The Riemann sum for the curvature plot was calculated for a SSR spanning 6176 bp in *L. major* Chr22 (from 605990 to 612166) is shown as a vertical dotted line. This score is compared to the density function representing the population of RIIC scores for equal-length regions in the genome) (solid line). In this case, the difference is statistically significant (p<0.05).
(PDF)

Figure S2 **Genome wide curvature distribution for the Tritryps.** The DNA intrinsic curvature for the Tritryps genomes *L. major* (*−*), *T. brucei* (–) and *T. cruzi* (…), an external reference fragment of approx. 7 Mb from human chromosome 1 spanning from 137 Mb to 144 Mb (–) and *E. coli* BW2952 strain (–), were analyzed with the bend.it algorithm, using a 31 bp window and DNaseI+nucleosome positioning data parameters.
(PDF)

Figure S3 **Box plots of the intrinsic curvature distribution across** ***Leishmania*** **chromosomes.** Chromosomes are depicted in ascending order from left to right. Upper panel: ***L. major*** chromosomes 1 to 36. Lower panel: ***L. infantum*** chromosomes 0 to 36; ***L. braziliensis*** chromosomes 0 to 35; ***L. mexicana*** chromosomes 0 to 34 from *L. mexicana.* For lower panels axes are as in the upper panel.
(PDF)

Figure S4 **Relationship between peaks of high predicted intrinsic curvature and chromosome length. A.** The number of IC peaks greater than 9 degrees per helical turn in each chromosome was plotted against the chromosome length. **B.** The average frequency of IC peaks greater than 9 degrees per helical turn (calculated as the chromosome length divided by the absolute number of peaks) is plotted against chromosome number.
(PDF)

Figure S5 **Graphical representation of IC peaks on all** ***L. major*** **chromosomes.** Bar plots of IC positions with an IC value greater than 9 degrees per helical turn. Both DNA strands are depicted in grey below bar plots, overlaid with CDS features shown in blue. Features labeled as ncRNA, snRNA or snoRNAs are shown in green. tRNAs are shown in red. rRNAs are shown in brown.
(PDF)

Figure S6 **Graphical representation of IC for regions with high RIIC-score for all** ***L. major*** **chromosomes.** The graphs are the same as figure 1. IC for regions with high RIIC score are indicated at the top. Sites associated with acetylated H3 histone [18] are indicated as small vertical lines.
(PDF)

Figure S7 **Location conservation of high RIIC scoring regions in** ***L. major*** **and** ***L. infantum*** **chromosomes.** The graphs are the same as figure 1. Blast HSPs longer than 100 bp and with at least 80% similarity are displayed in red scale and blue lines represent inversions.
(PDF)

Table S1 **Genome intrinsic curvature in Tritryps.**
(PDF)

Table S2 **Chromosome intrinsic curvature in** ***L. major.***
(PDF)
