# Supplementary material for: Genomic Analysis of Sequence-Dependent DNA Curvature in Leishmania
Source: PLoS One. 2013 Apr 30;8(4):e63068. doi: 10.1371/journal.pone.0063068 (PMC3639952; doi:10.1371/journal.pone.0063068)
Supplement: Figure S1 — Regional Integrated Intrinsic Curvature analysis in L. major. The Riemann sum for the curvature plot was calculated for a SSR spanning 6176 bp in L. major Chr22 (from 605990 to 612166) is shown as a vertical dotted line. This score is compared to the density function representing the population of RIIC scores for equal-length regions in the genome) (solid line). In this case, the difference is statistically significant (p<0.05). (PDF) [file pone.0063068.s001.pdf]

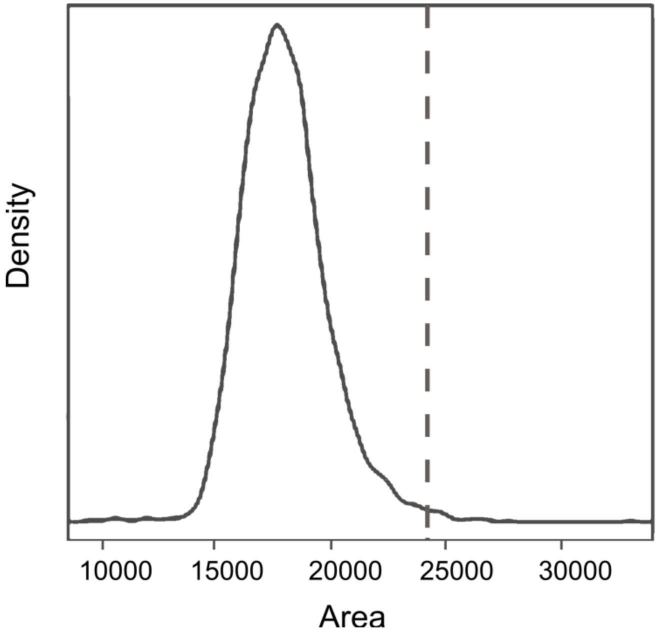

S Figure 1. Regional Integrated Intrinsic Curvature analysis in *L. major*

The Riemann sum for the curvature plot was calculated for a SSR spanning 6176bp in *L. major* Chr22 (from 605990 to 612166) is shown as a vertical dotted line. This score is compared to the density function representing the population of RIIC scores for equal-length regions in the genome) (solid line). In this case, the difference is statistically significant ($p < 0.05$).
