# Supplementary material for: Genomic Analysis of Sequence-Dependent DNA Curvature in Leishmania
Source: PLoS One. 2013 Apr 30;8(4):e63068. doi: 10.1371/journal.pone.0063068 (PMC3639952; doi:10.1371/journal.pone.0063068)
Supplement: Figure S2 — Genome wide curvature distribution for the Tritryps. The DNA intrinsic curvature for the Tritryps genomes L. major (−), T. brucei (–) and T. cruzi (…), an external reference fragment of approx. 7 Mb from human chromosome 1 spanning from 137 Mb to 144 Mb (–) and E. coli BW2952 strain (–), were analyzed with the bend.it algorithm, using a 31 bp window and DNaseI+nucleosome positioning data parameters. (PDF) [file pone.0063068.s002.pdf]

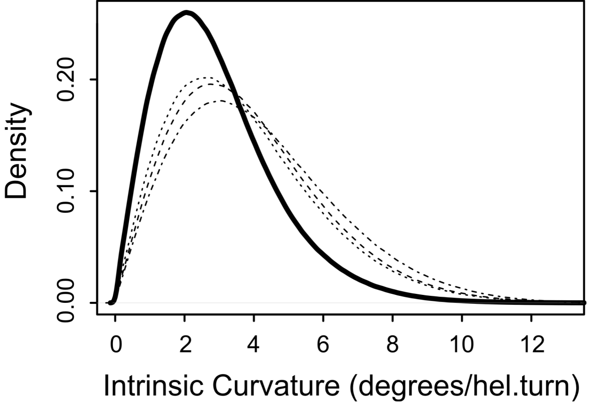

S Figure 2. Genome wide curvature distribution for the Tritryps.

The DNA intrinsic curvature for the Tritryps genomes *L. major* (–), *T. brucei* (- -)and *T. cruzi* (…), an external reference fragment of approx. 7Mb from human chromosome 1 spanning from 137Mb to 144Mb (·-) and *E. coli* BW2952 strain (- -), were analyzed with the bend.it algorithm, using a 31bp window and DNaseI + nucleosome positioning data parameters.
