# Supplementary material for: Genomic Analysis of Sequence-Dependent DNA Curvature in Leishmania
Source: PLoS One. 2013 Apr 30;8(4):e63068. doi: 10.1371/journal.pone.0063068 (PMC3639952; doi:10.1371/journal.pone.0063068)
Supplement: Figure S3 — Box plots of the intrinsic curvature distribution across Leishmania chromosomes. Chromosomes are depicted in ascending order from left to right. Upper panel: L. major chromosomes 1 to 36. Lower panel: L. infantum chromosomes 0 to 36; L. braziliensis chromosomes 0 to 35; L. mexicana chromosomes 0 to 34 from L. mexicana. For lower panels axes are as in the upper panel. (PDF) [file pone.0063068.s003.pdf]

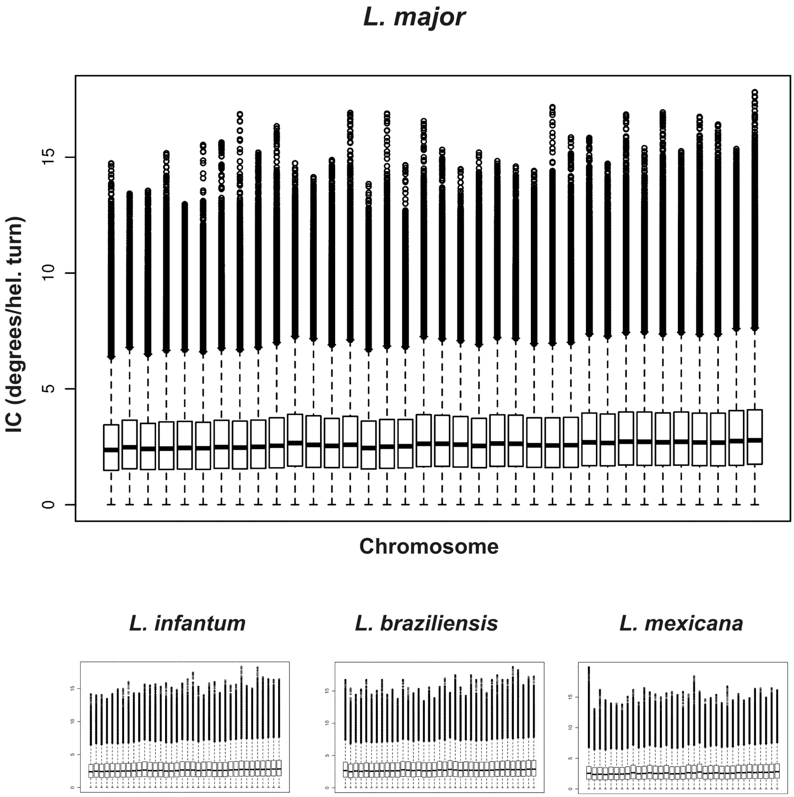

S Figure 3. Box plots of the intrinsic curvature distribution across *Leishmania* chromosomes.

Chromosomes are depicted in ascending order from left to right. Upper panel: ***L. major*** chromosomes 1 to 36. Lower panel: ***L. infantum*** chromosomes 0 to 36; ***L. braziliensis*** chromosomes 0 to 35; ***L. mexicana*** chromosomes 0 to 34 from *L. mexicana.* For lower panels axes are as in the upper panel.
