# Supplementary material for: Genomic Analysis of Sequence-Dependent DNA Curvature in Leishmania
Source: PLoS One. 2013 Apr 30;8(4):e63068. doi: 10.1371/journal.pone.0063068 (PMC3639952; doi:10.1371/journal.pone.0063068)
Supplement: Figure S4 — Relationship between peaks of high predicted intrinsic curvature and chromosome length. A. The number of IC peaks greater than 9 degrees per helical turn in each chromosome was plotted against the chromosome length. B. The average frequency of IC peaks greater than 9 degrees per helical turn (calculated as the chromosome length divided by the absolute number of peaks) is plotted against chromosome number. (PDF) [file pone.0063068.s004.pdf]

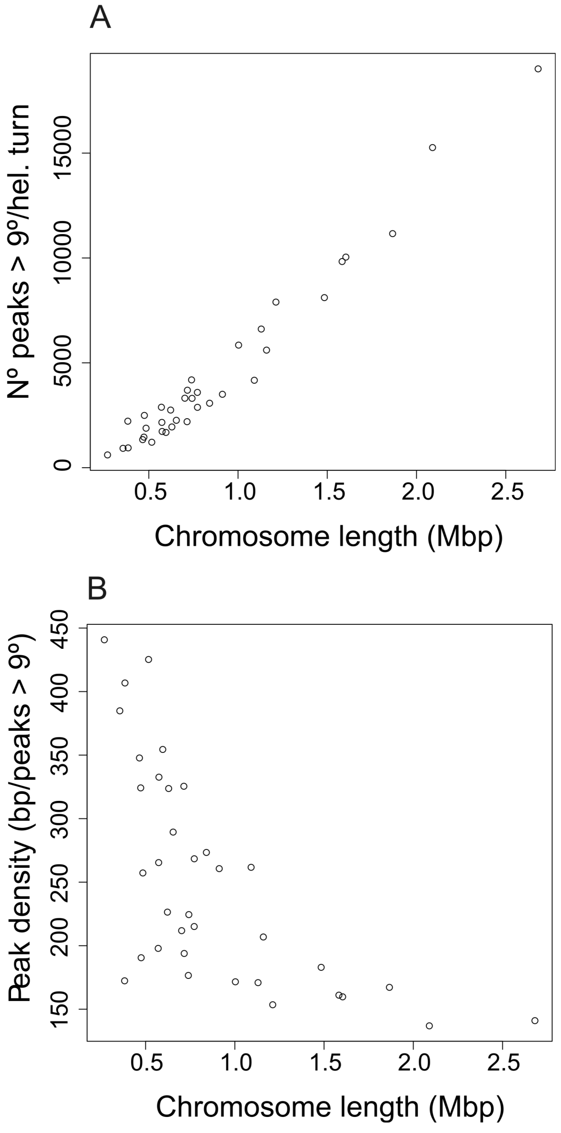

S Figure 4. Relationship between peaks of high predicted intrinsic curvature and chromosome length.

A. The number of IC peaks greater than 9 degrees per helical turn in each chromosome was plotted against the chromosome length. **B.** The average frequency of IC peaks greater than 9 degrees per helical turn (calculated as the chromosome length divided by the absolute number of peaks) is plotted against chromosome number.
