# Supplementary material for: Genomic Analysis of Sequence-Dependent DNA Curvature in Leishmania
Source: PLoS One. 2013 Apr 30;8(4):e63068. doi: 10.1371/journal.pone.0063068 (PMC3639952; doi:10.1371/journal.pone.0063068)
Supplement: Figure S5 — Graphical representation of IC peaks on all L. major chromosomes. Bar plots of IC positions with an IC value greater than 9 degrees per helical turn. Both DNA strands are depicted in grey below bar plots, overlaid with CDS features shown in blue. Features labeled as ncRNA, snRNA or snoRNAs are shown in green. tRNAs are shown in red. rRNAs are shown in brown. (PDF) [file pone.0063068.s005.pdf]

S Figure 5. Graphical representation of IC peaks on all *L. major* chromosomes

Bar plots of IC positions with an IC value greater than 9 degrees per helical turn. Both DNA strands are depicted in grey below bar plots, overlayed with CDS features shown in blue. Features labeled as ncRNA, snRNA or snoRNAs are shown in green. tRNAs are shown in red. rRNAs are shown in brown.

Chromosome 1

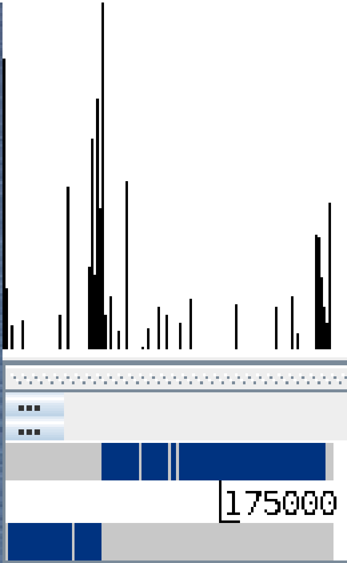

Chromosome 2

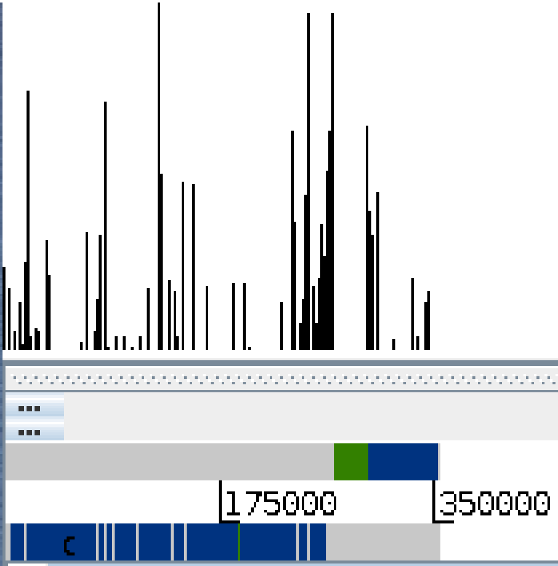

Chromosome 3

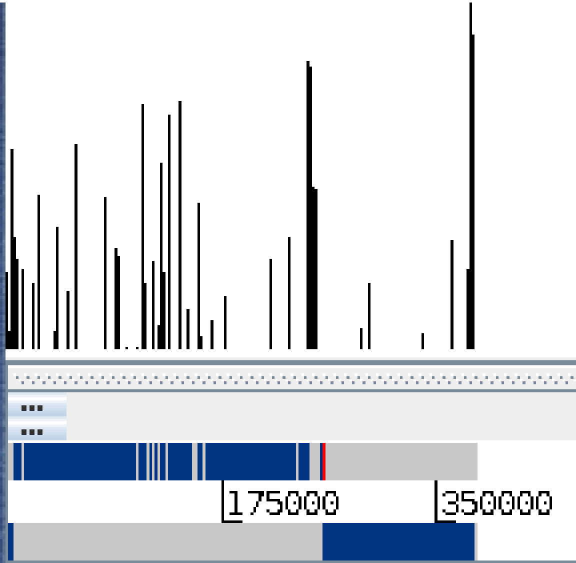

Chromosome 4

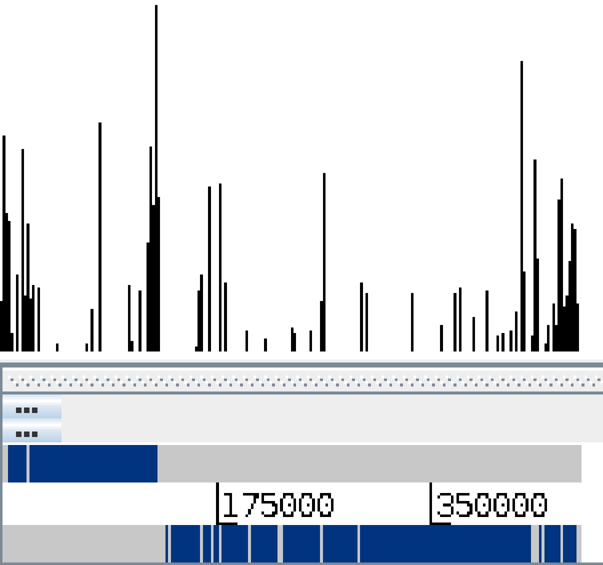

Chromosome 5

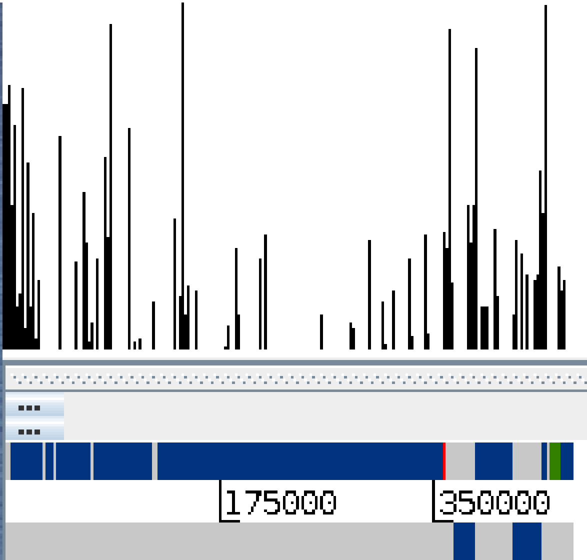

Chromosome 6

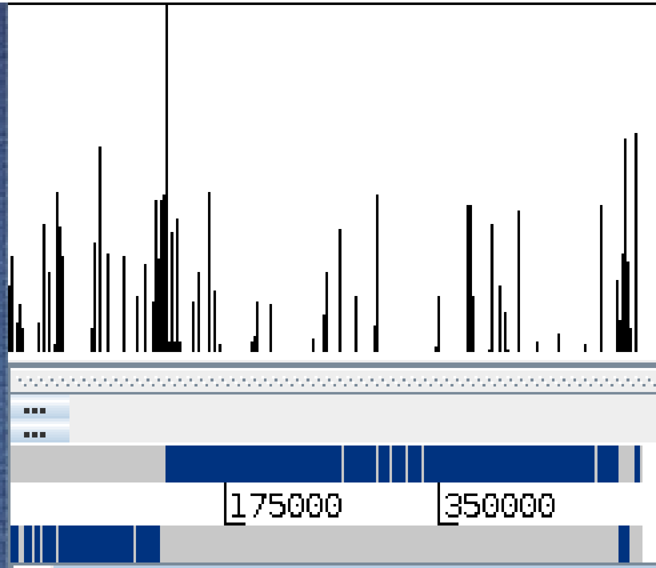

Chromosome 7

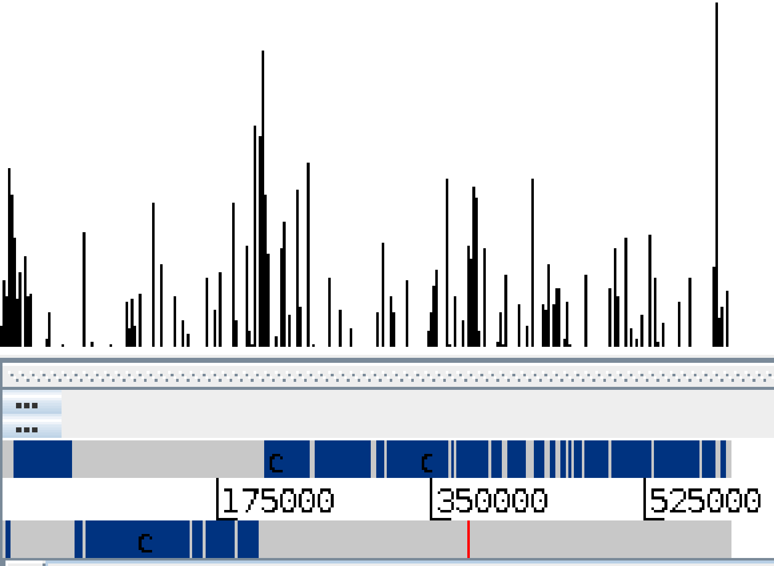

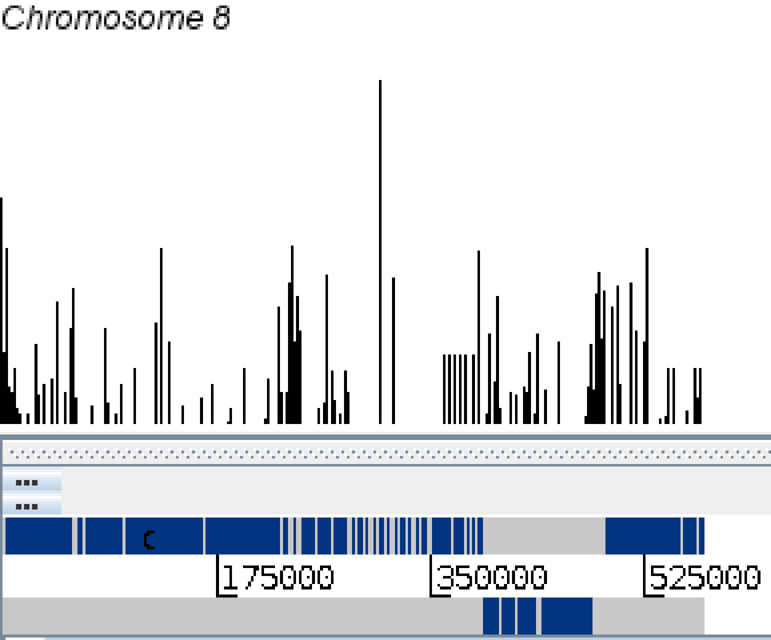
Chromosome 8
C
175000
350000
525000

Chromosome 9

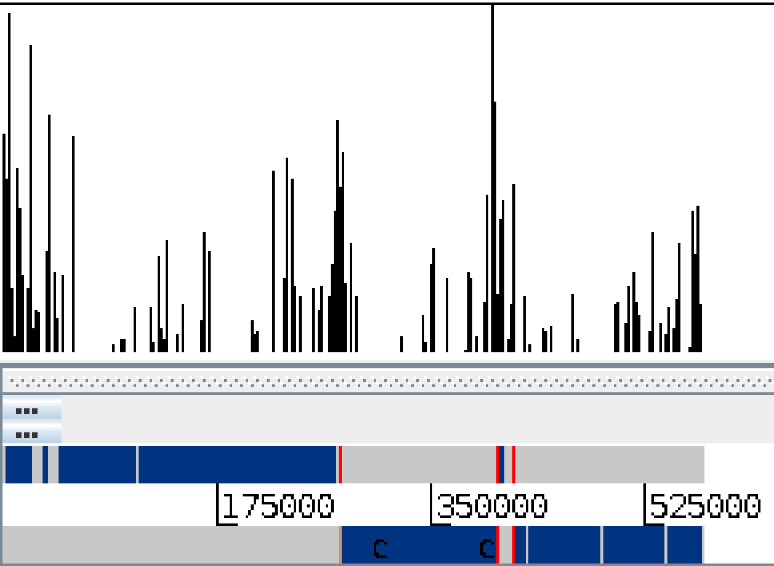

Chromosome 10

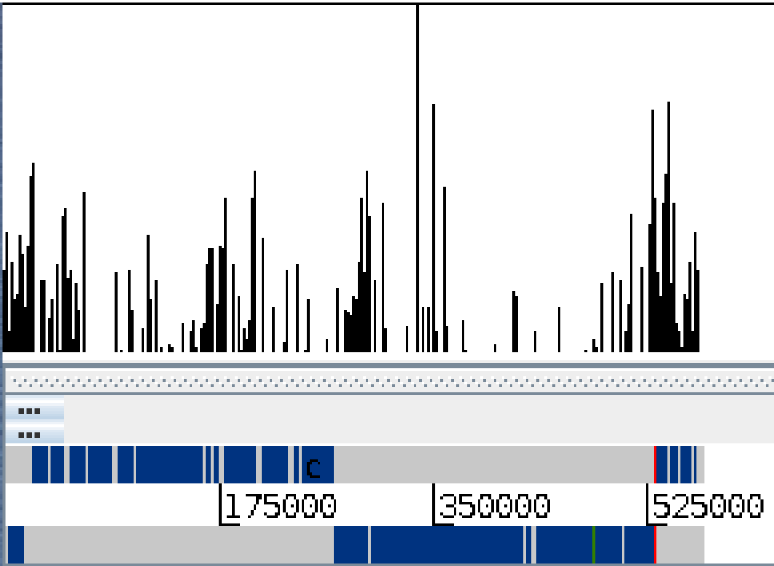

Chromosome 11

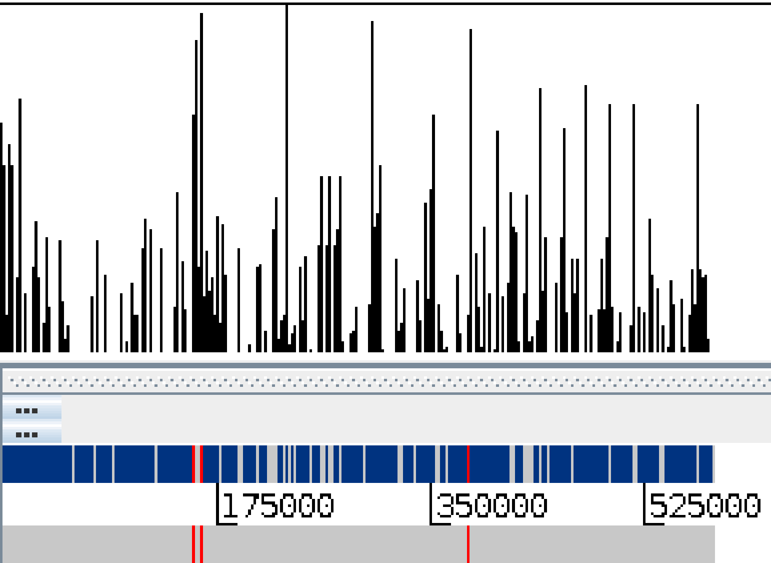

Chromosome 12

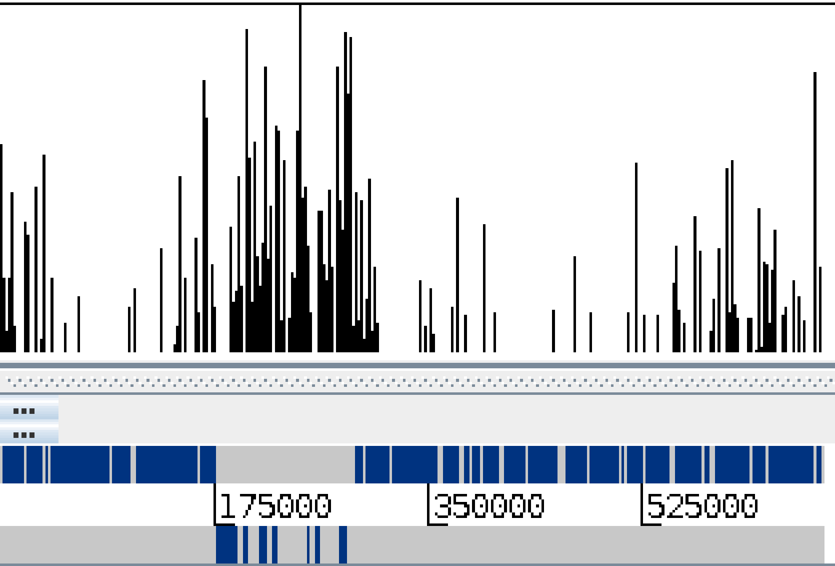

Chromosome 13

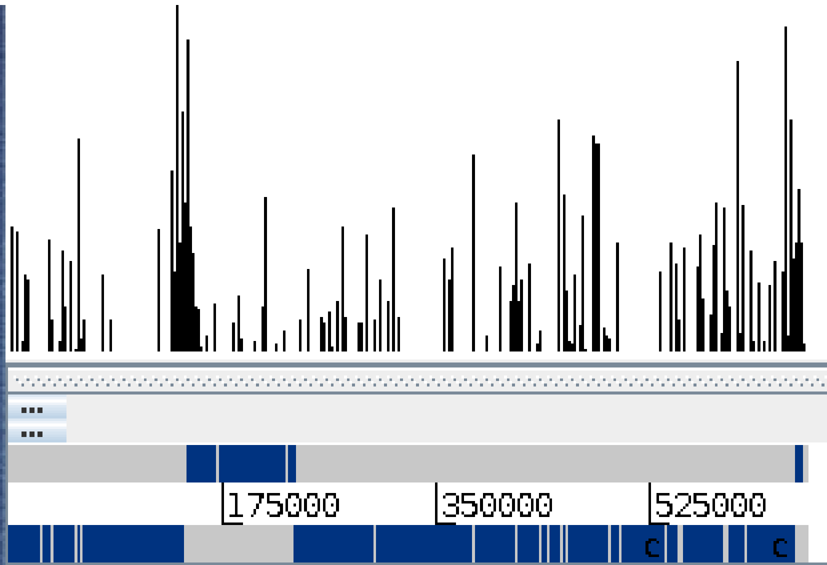

Chromosome 14

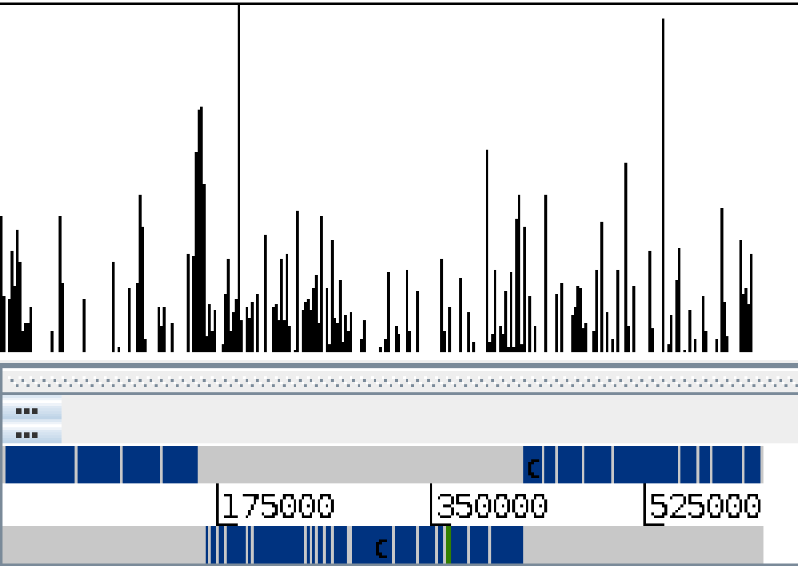

Chromosome 15

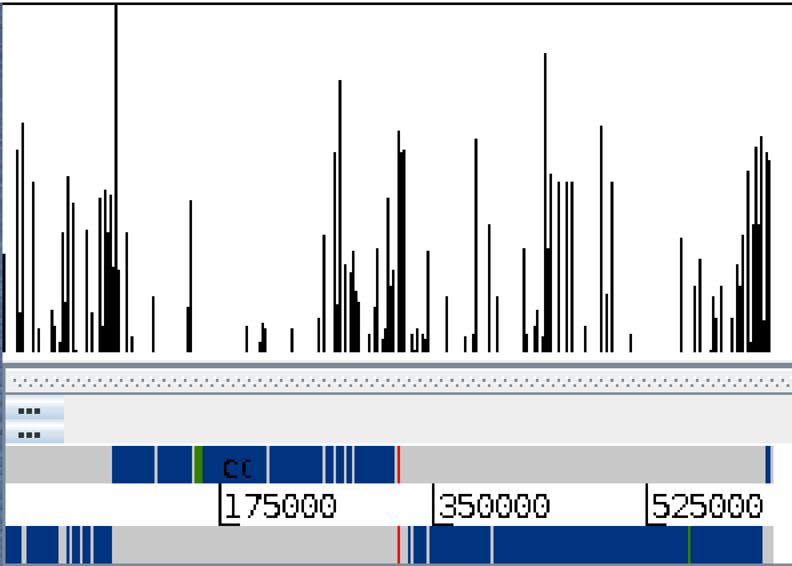

Chromosome 16

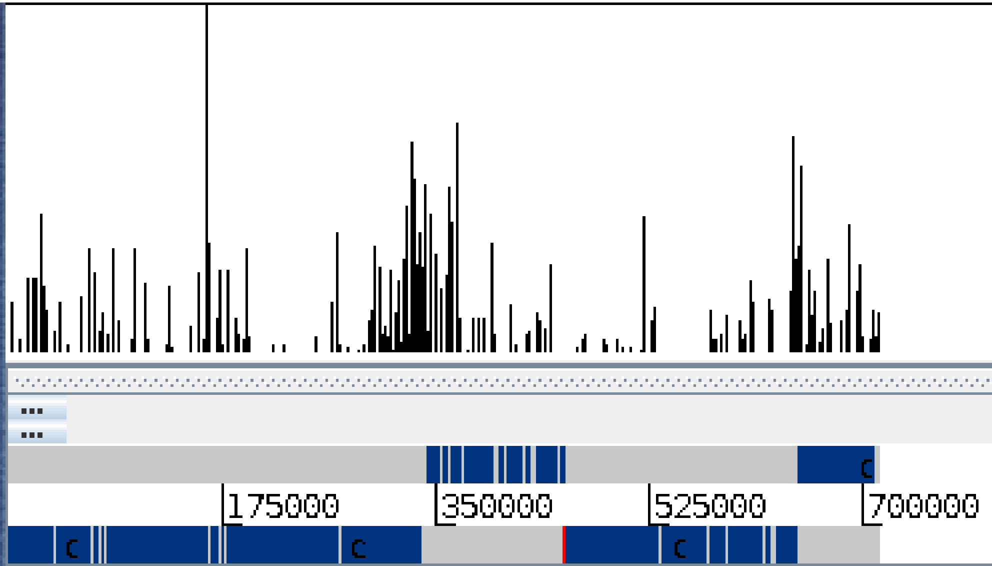

Chromosome 17

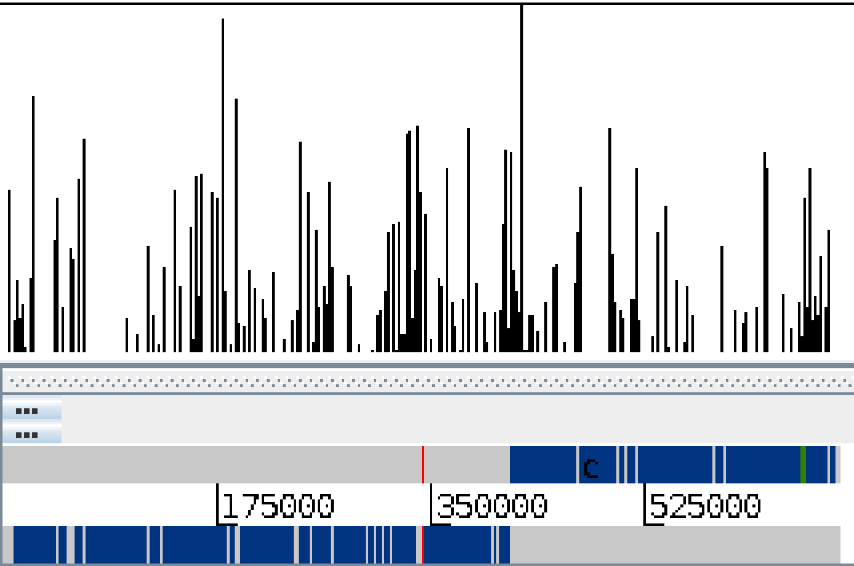

Chromosome 18

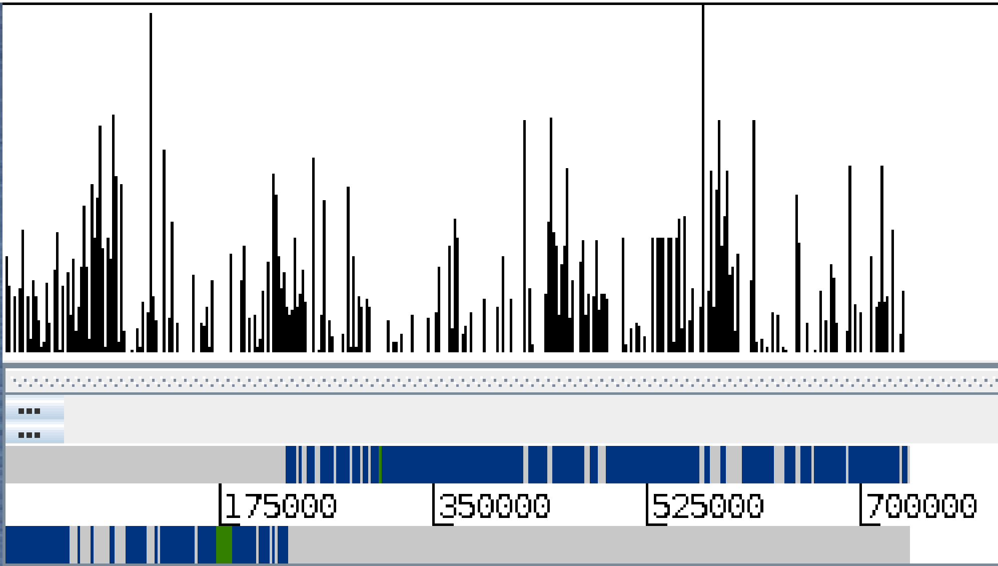

Chromosome 19

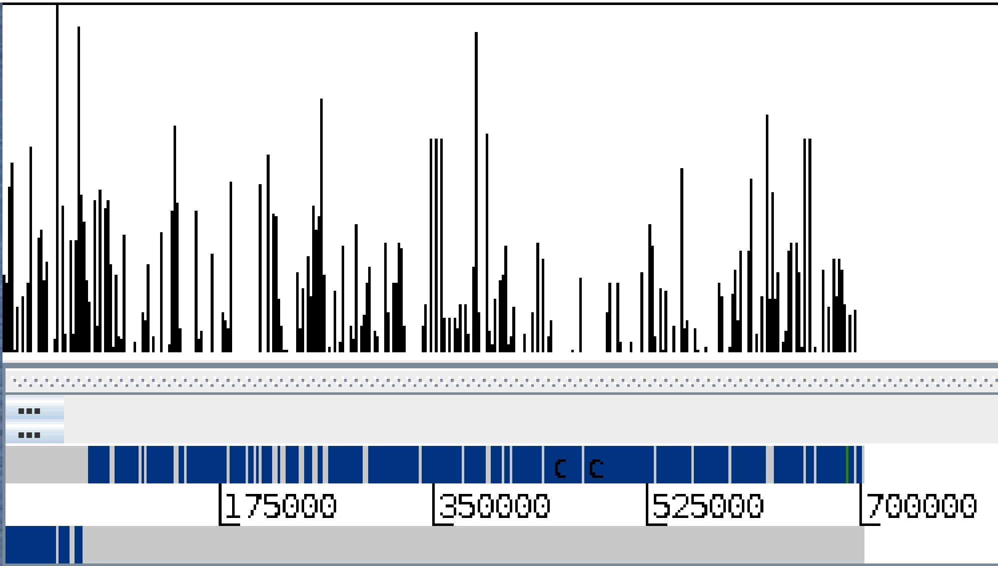

Chromosome 20

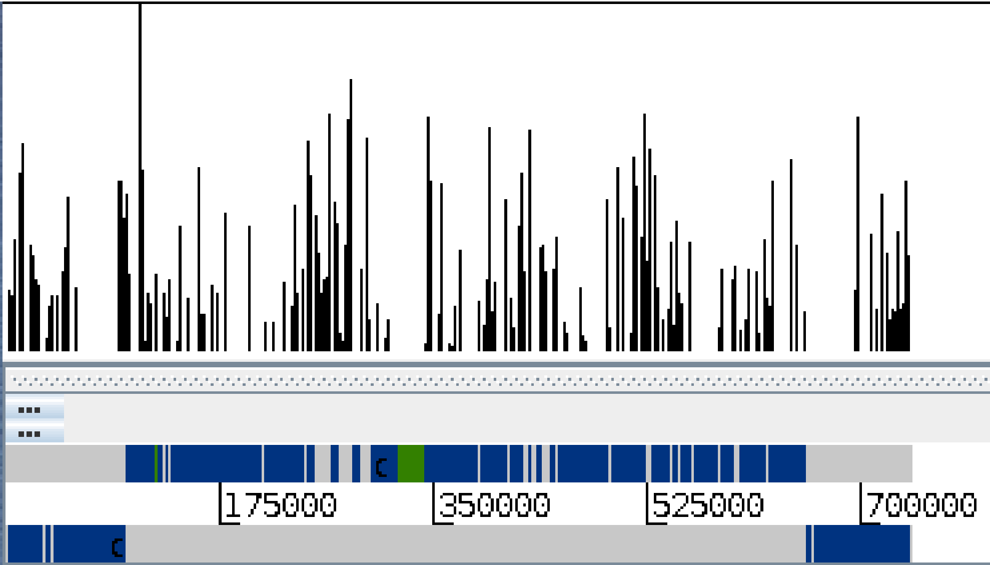

Chromosome 21

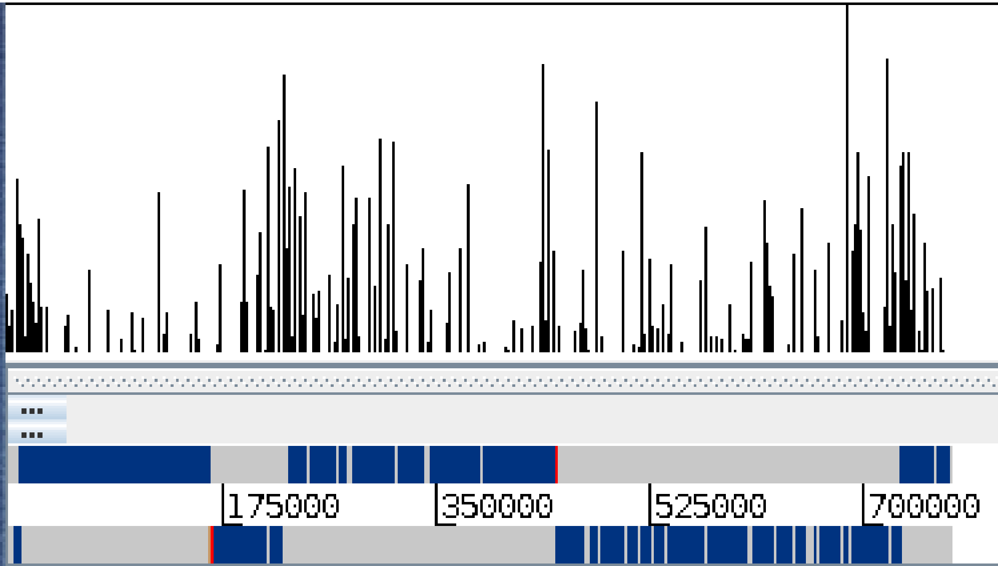

Chromosome 22

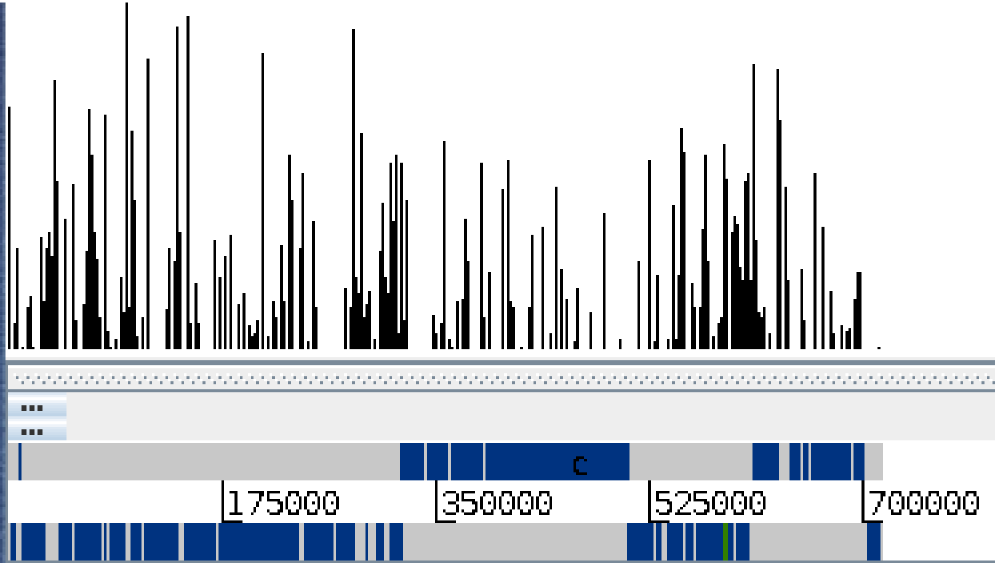

Chromosome 23

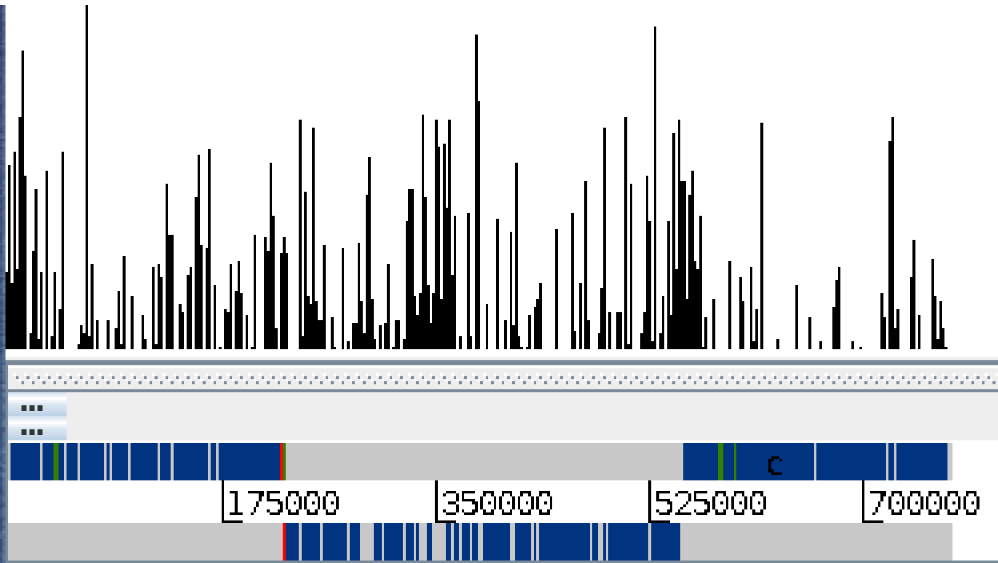

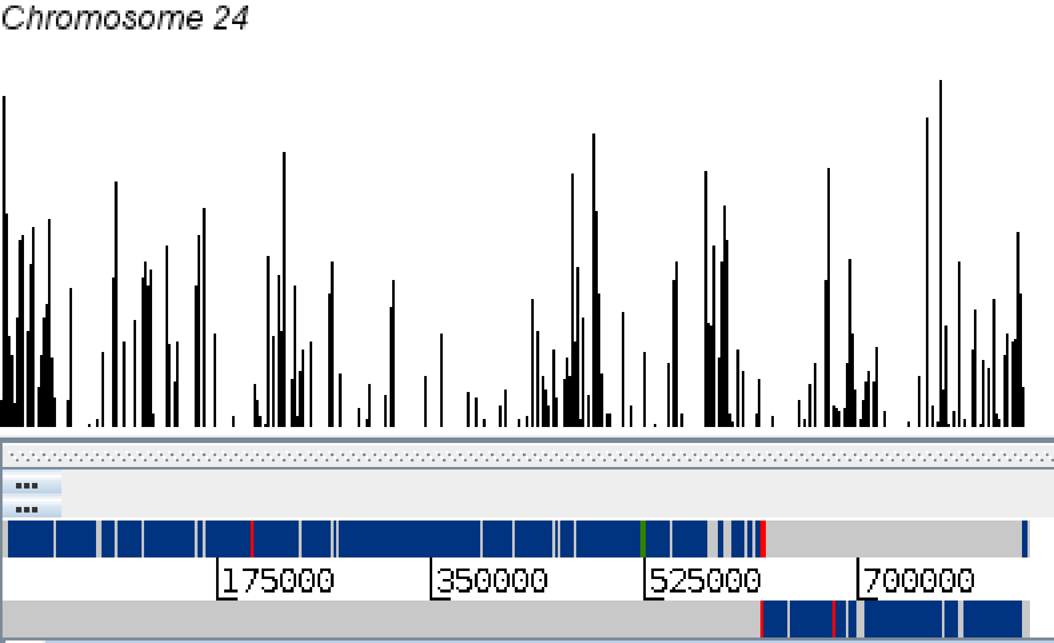
Chromosome 24
175000
350000
525000
700000

Chromosome 25

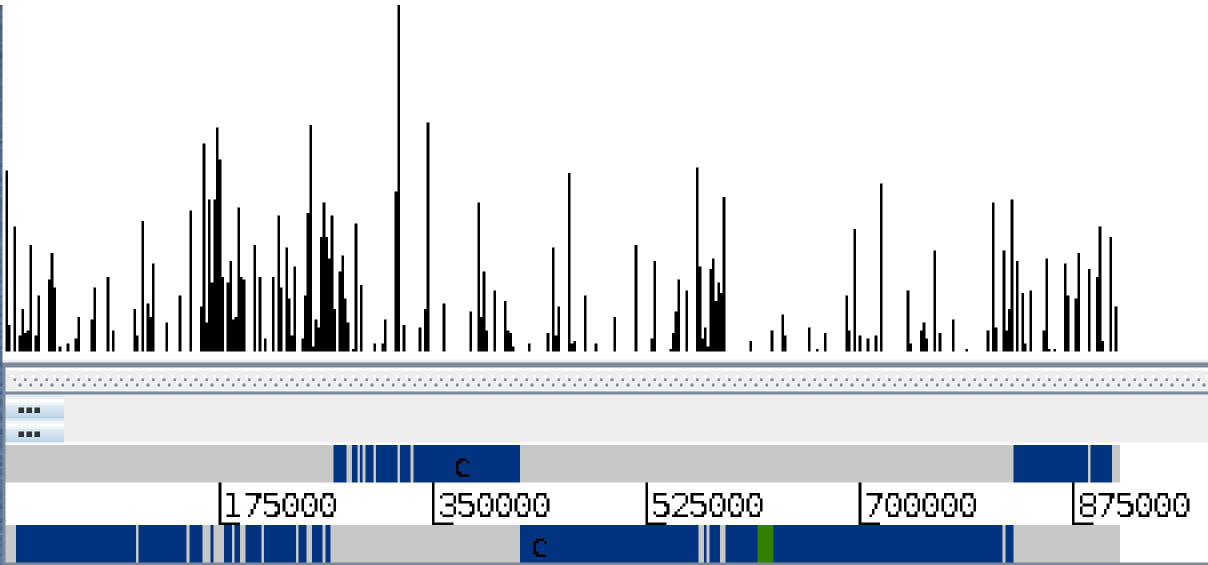

Chromosome 26

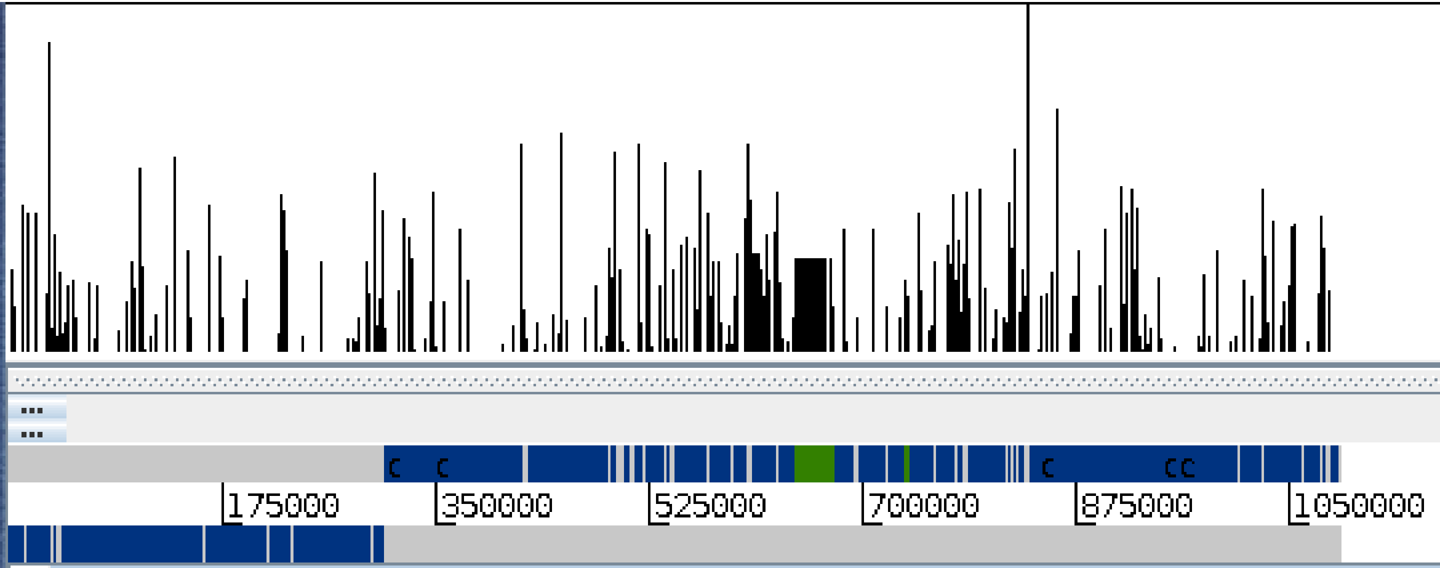

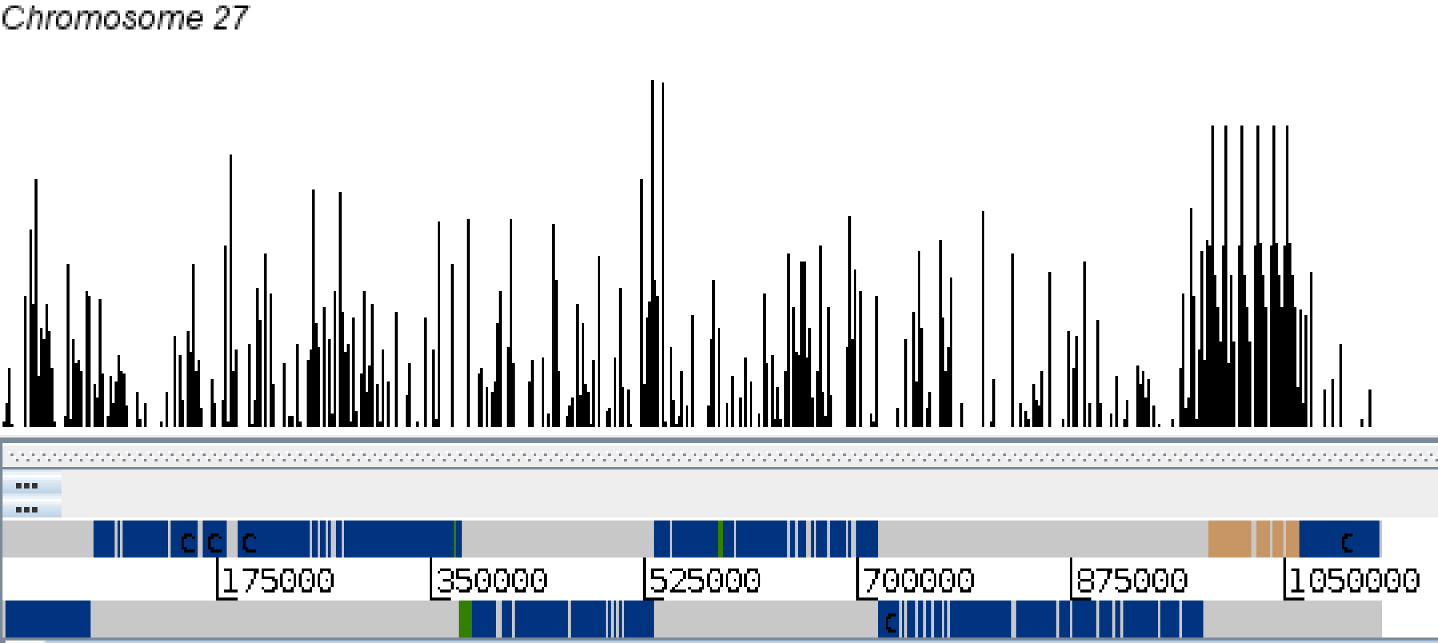
Chromosome 27
175000
350000
525000
700000
875000
1050000

Chromosome 28

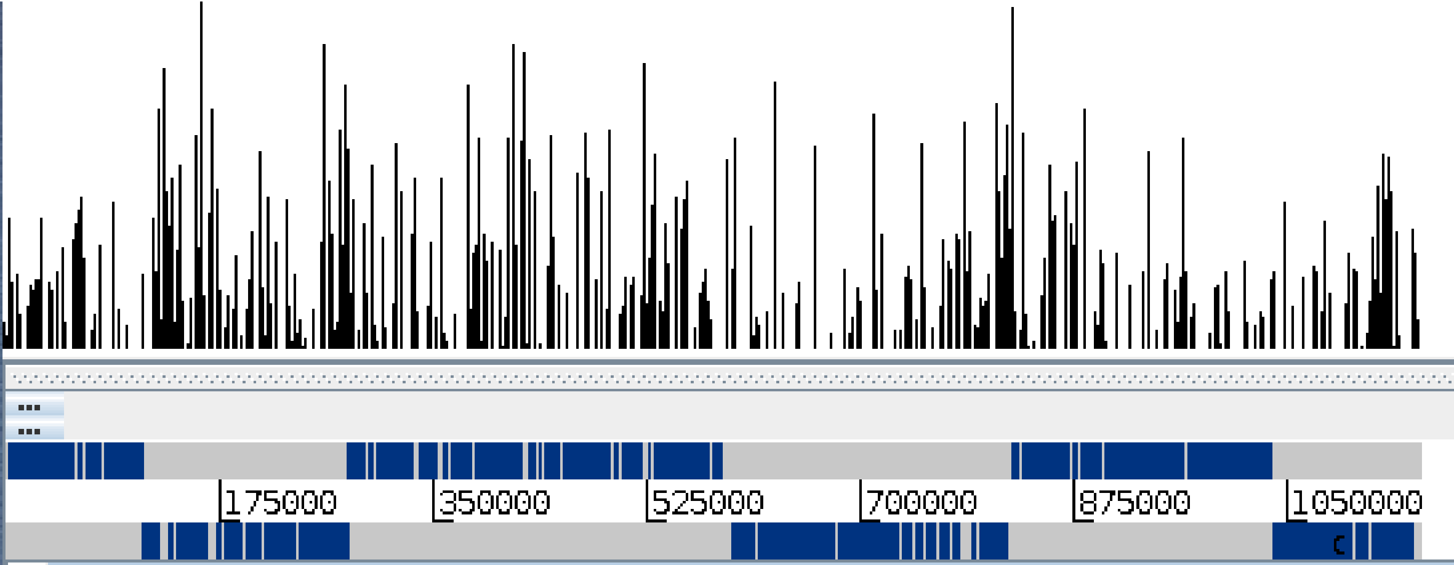

Chromosome 29

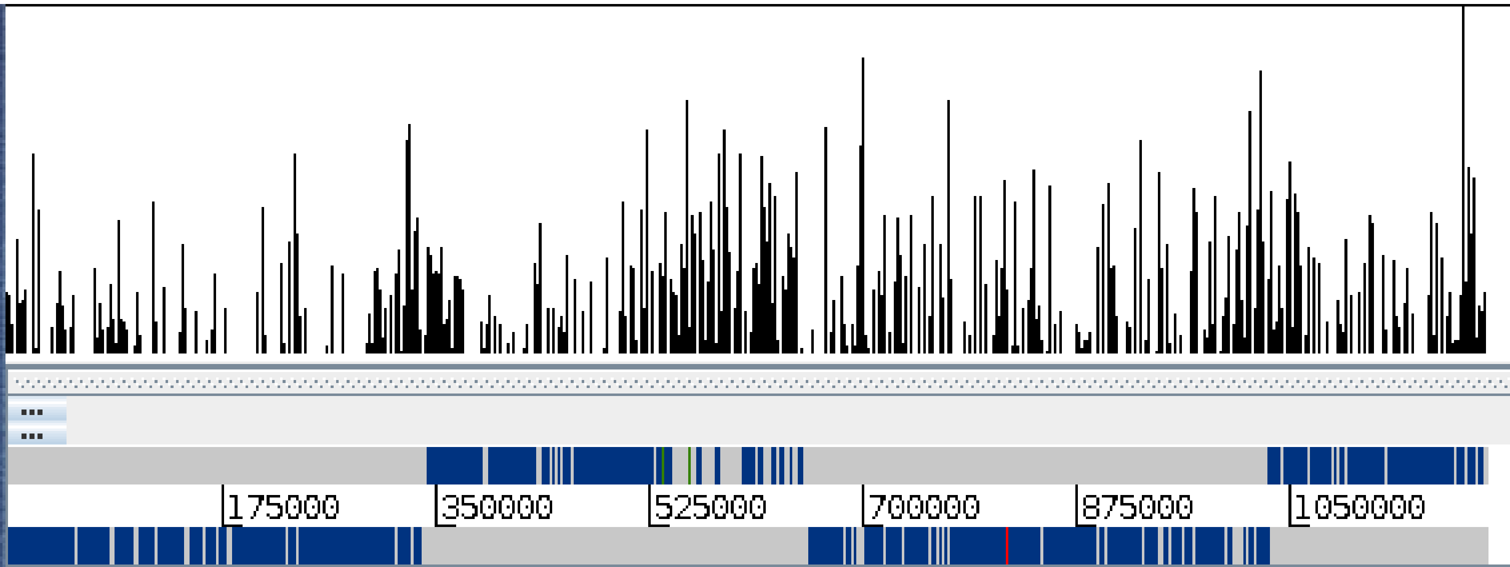

Chromosome 30

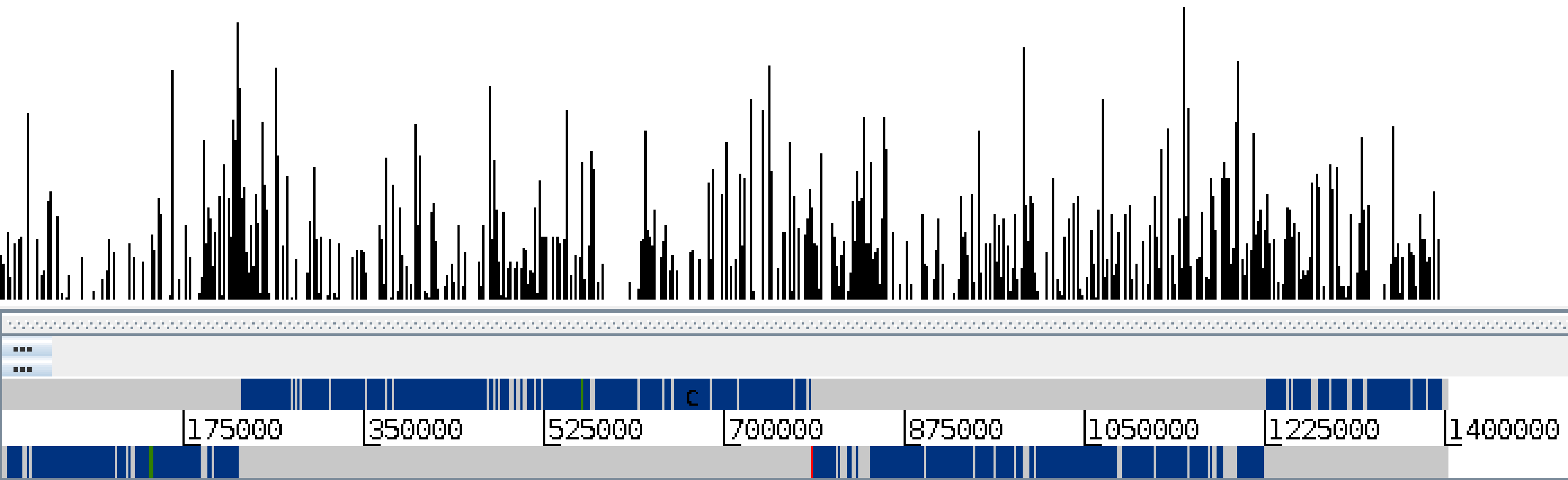

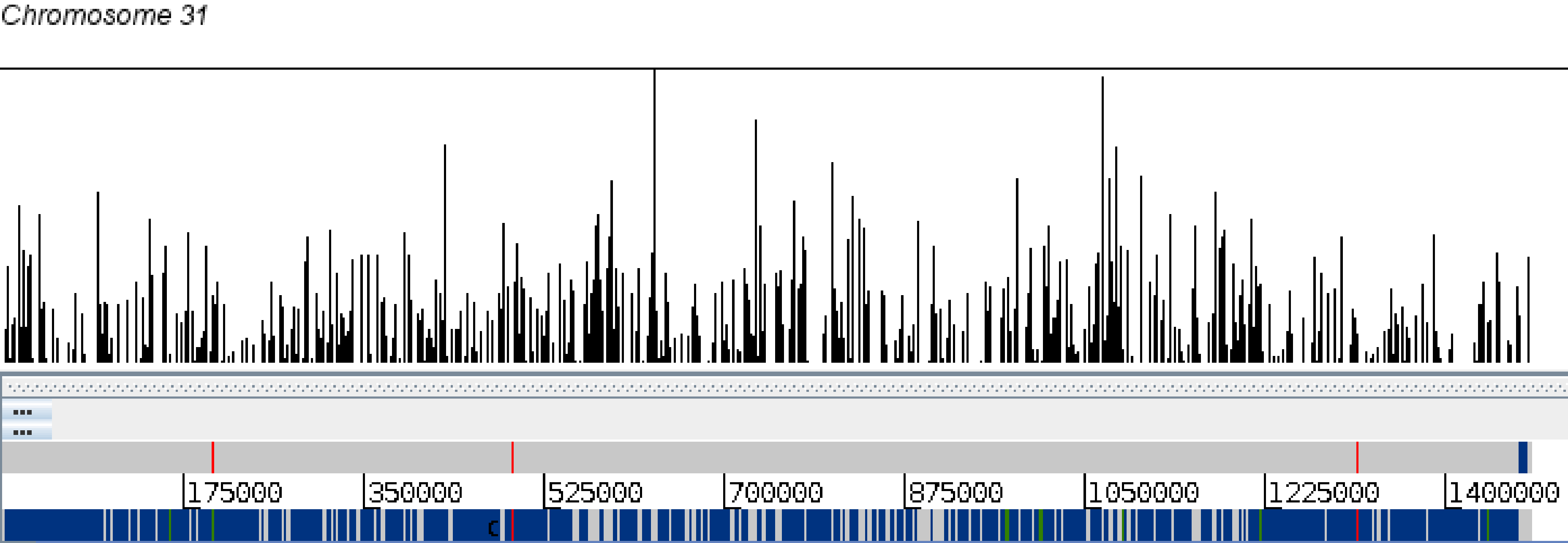
Chromosome 31
175000
350000
525000
700000
875000
1050000
1225000
1400000

Chromosome 32

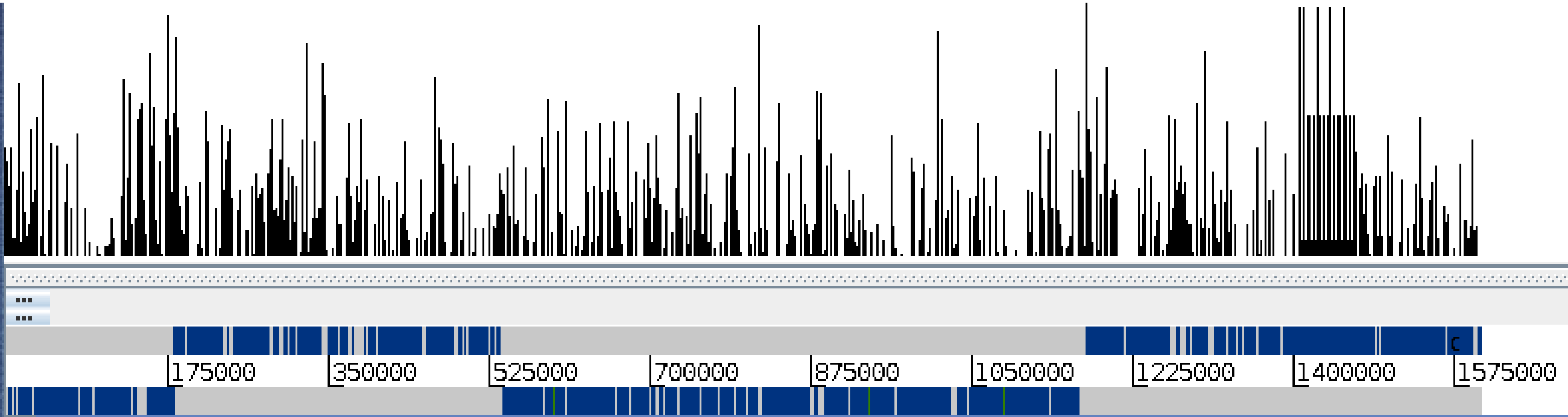

Chromosome 33

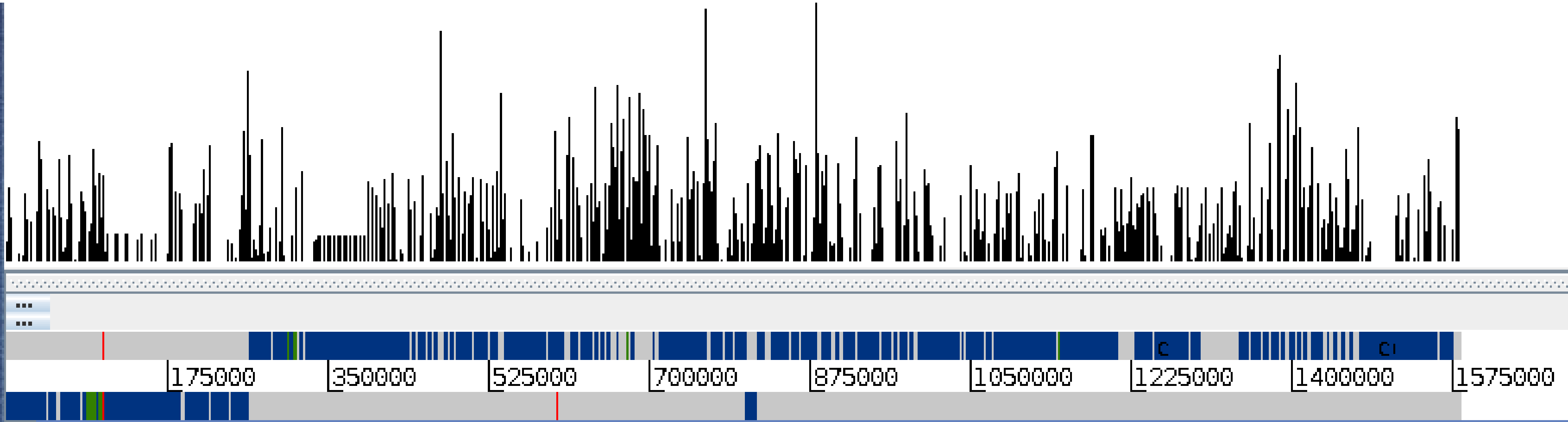

Chromosome 34

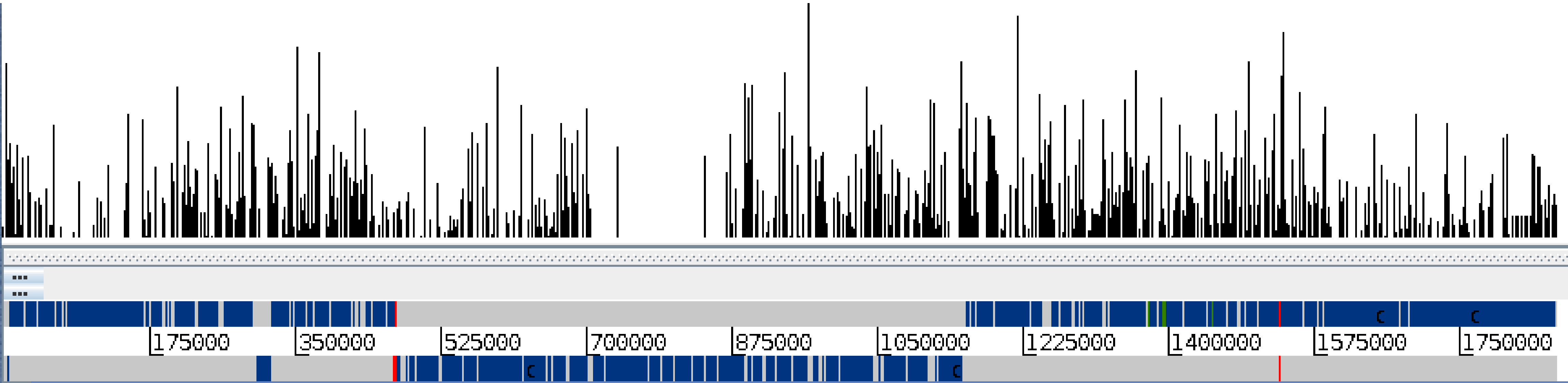

Chromosome 35

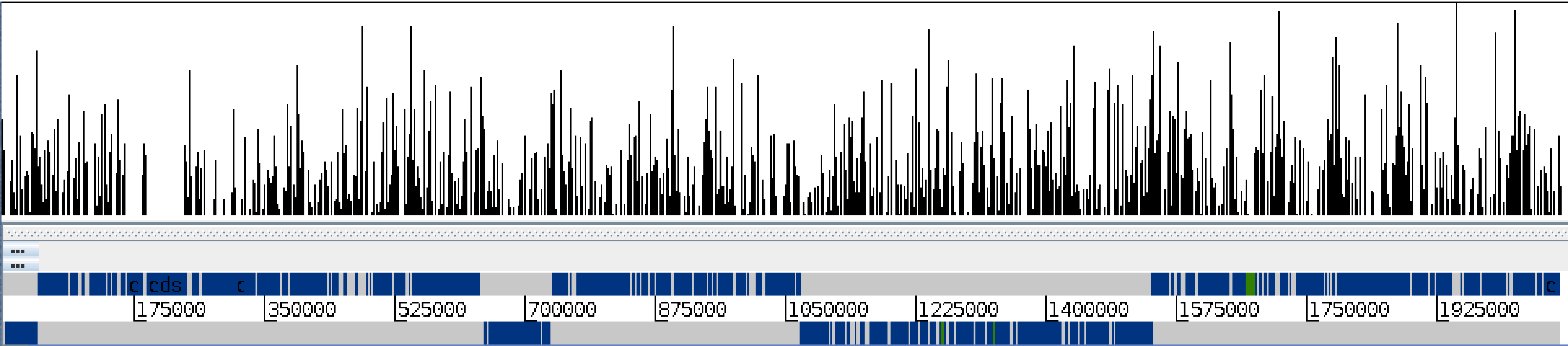

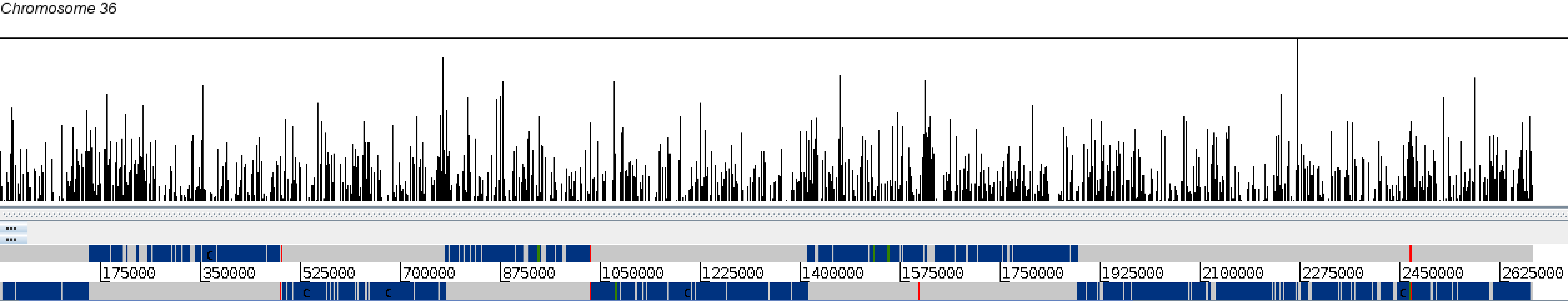

Chromosome 36
c
175000
350000
525000
700000
875000
1050000
1225000
1400000
1575000
1750000
1925000
2100000
2275000
2450000
2625000
