# Supplementary material for: Genomic Analysis of Sequence-Dependent DNA Curvature in Leishmania
Source: PLoS One. 2013 Apr 30;8(4):e63068. doi: 10.1371/journal.pone.0063068 (PMC3639952; doi:10.1371/journal.pone.0063068)
Supplement: Figure S6 — Graphical representation of IC for regions with high RIIC-score for all L. major chromosomes. The graphs are the same as figure 1. IC for regions with high RIIC score are indicated at the top. Sites associated with acetylated H3 histone [18] are indicated as small vertical lines. (PDF) [file pone.0063068.s006.pdf]

S Figure 6. Graphical representation of IC for regions with high RIIC-score for all *L. major* chromosomes.

The graphs are the same as figure 1. IC for regions with high RIIC score are indicated at the top. Sites associated with acetylated H3 histone (19) are indicated as small vertical lines.

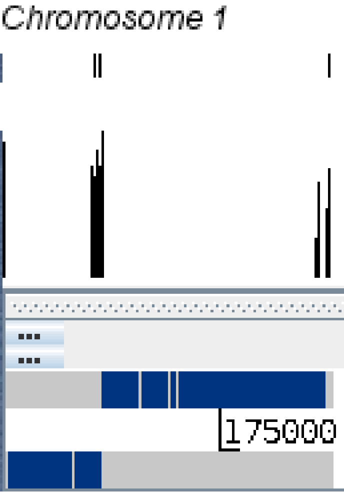
Chromosome 1
175000

Chromosome 2

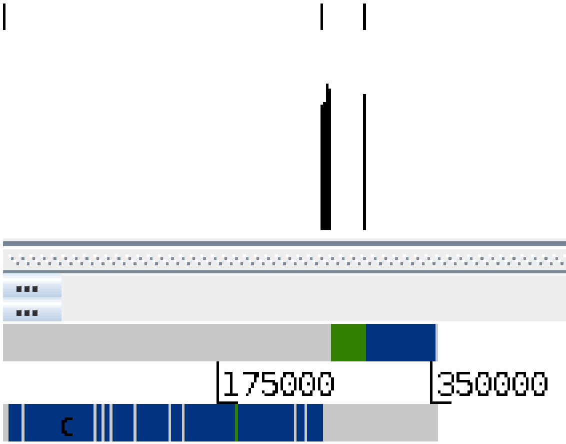

Chromosome 3

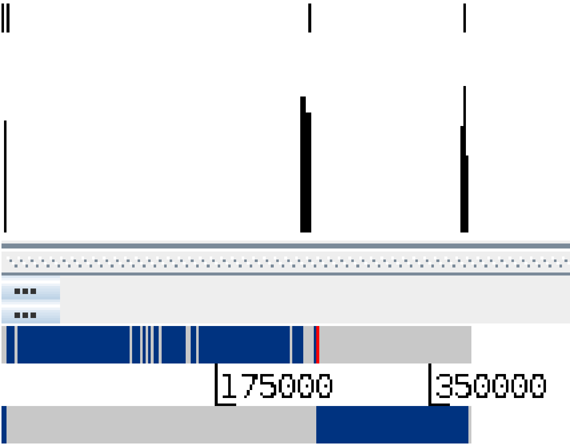

Chromosome 4

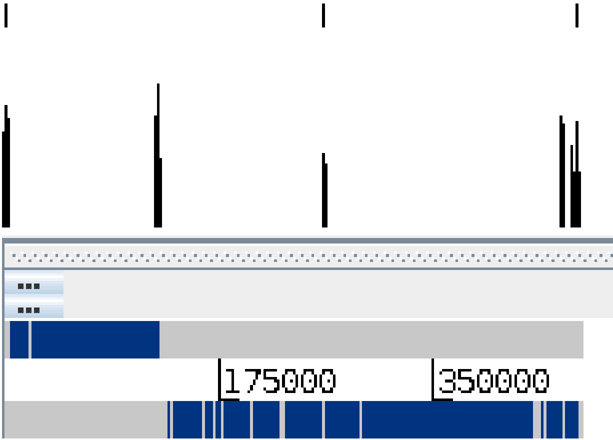

Chromosome 5

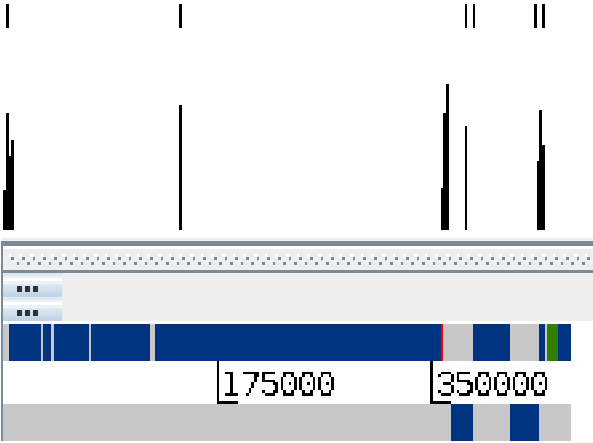

Chromosome 6

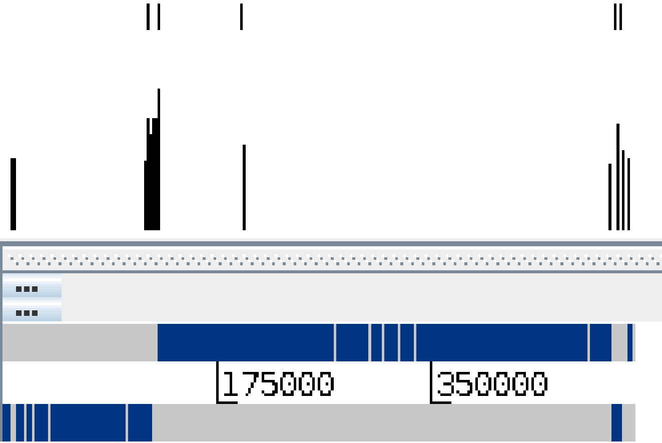

Chromosome 7

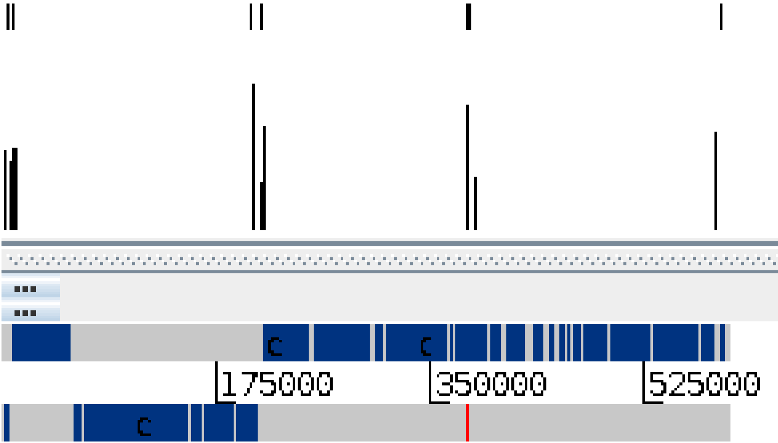

Chromosome 8

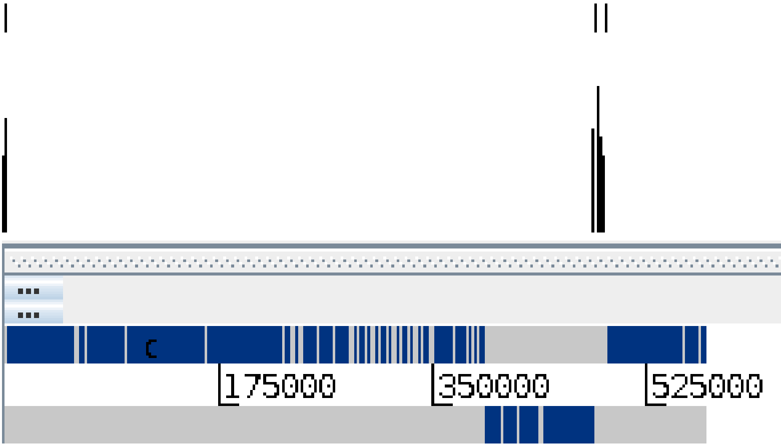

Chromosome 9

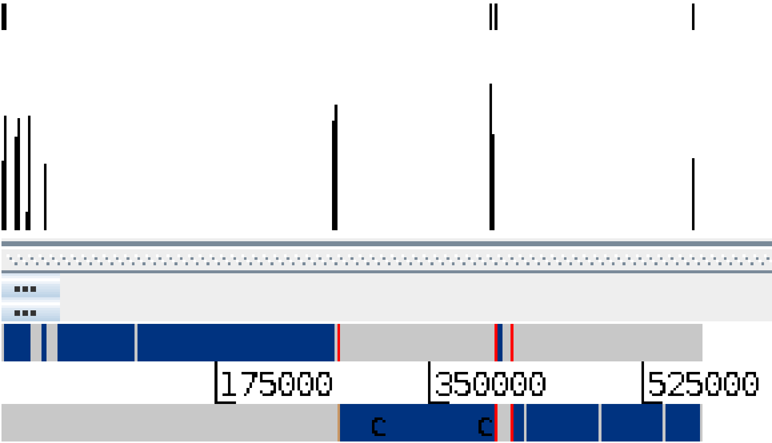

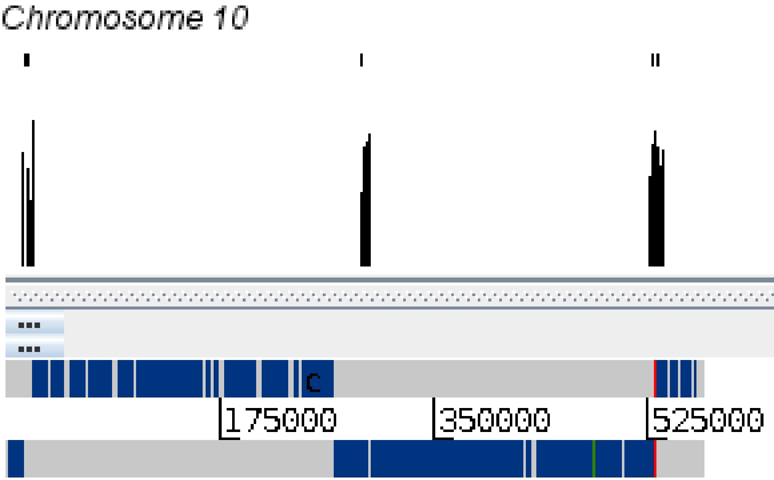

Chromosome 10
C
175000
350000
525000

Chromosome 11

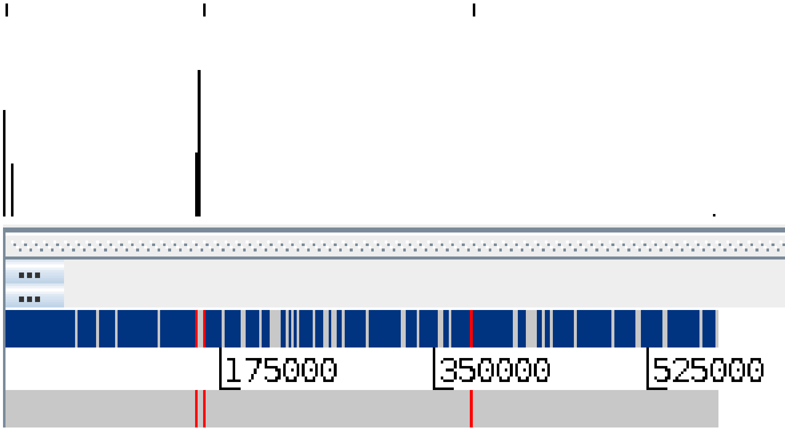

Chromosome 12

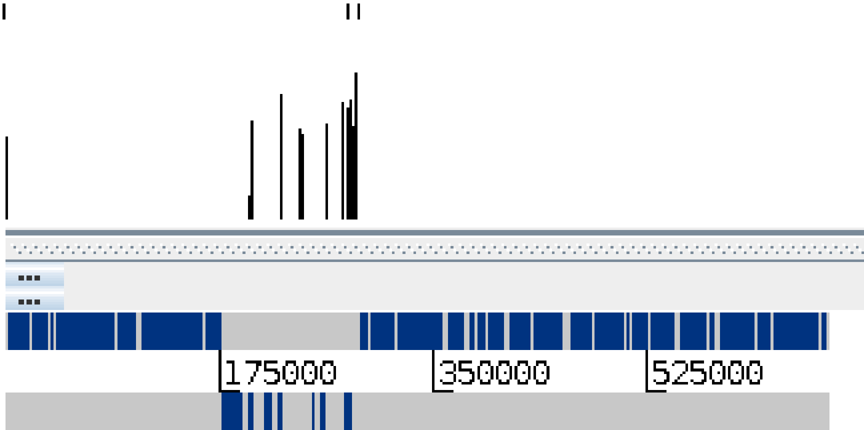

Chromosome 13

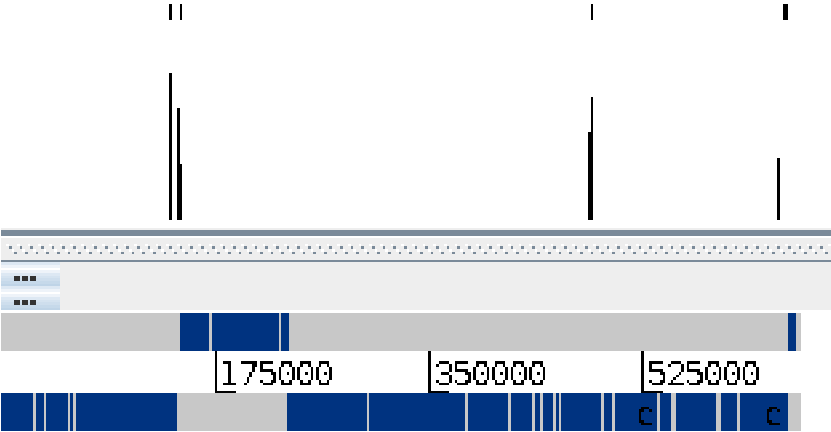

Chromosome 14

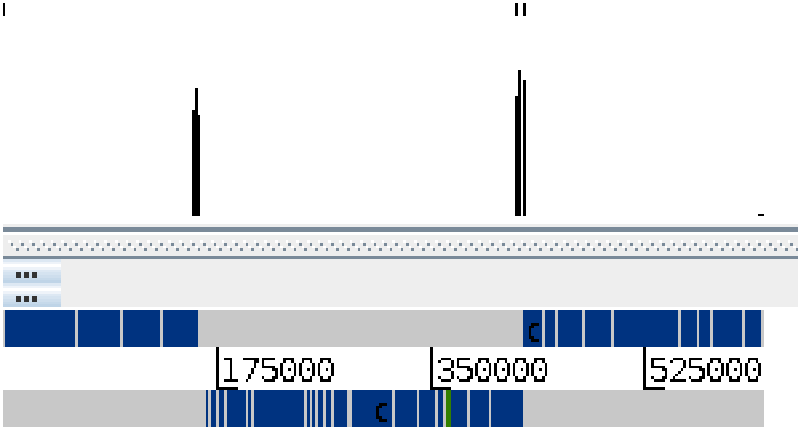

Chromosome 15

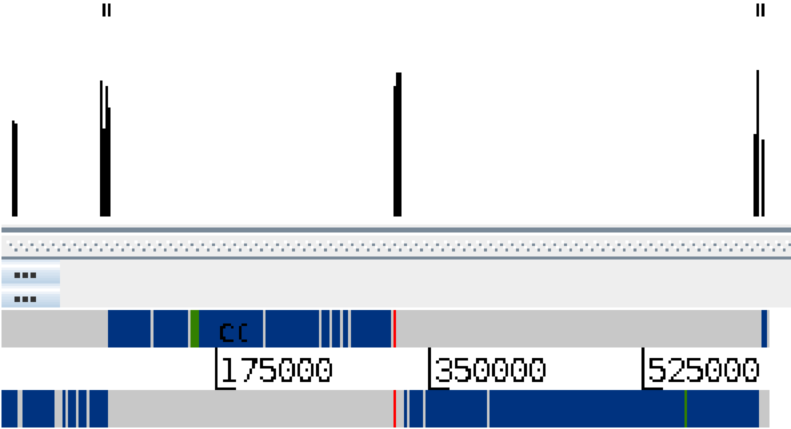

Chromosome 16

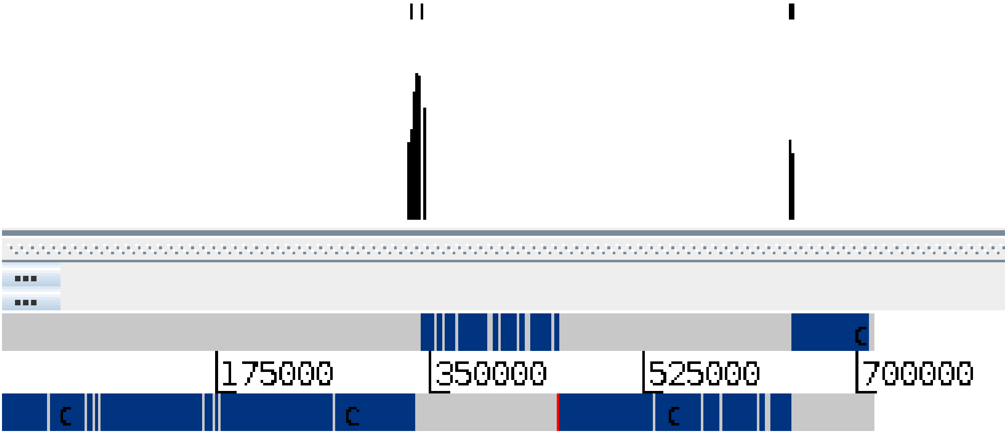

Chromosome 17

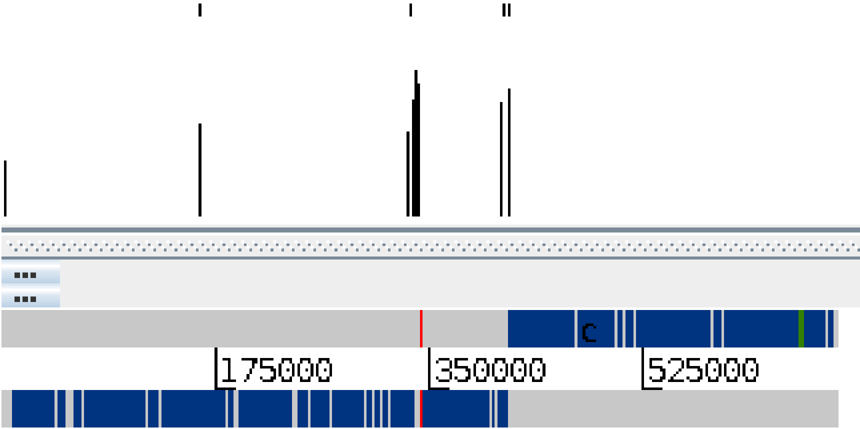

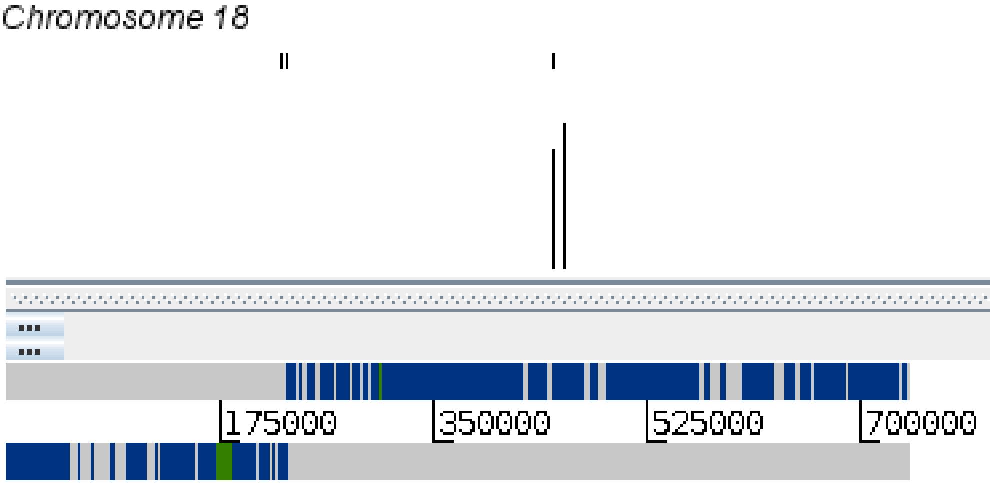
Chromosome 18
175000
350000
525000
700000

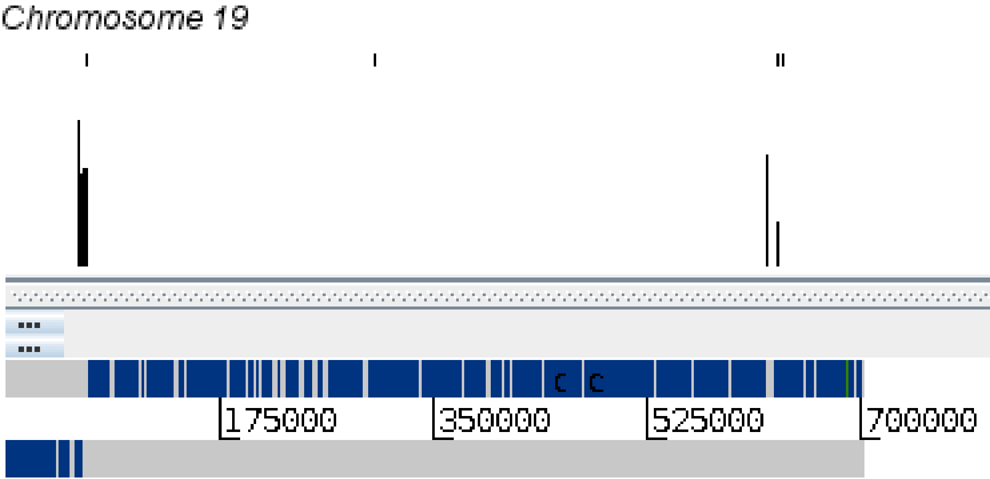
Chromosome 19
175000
350000
525000
700000

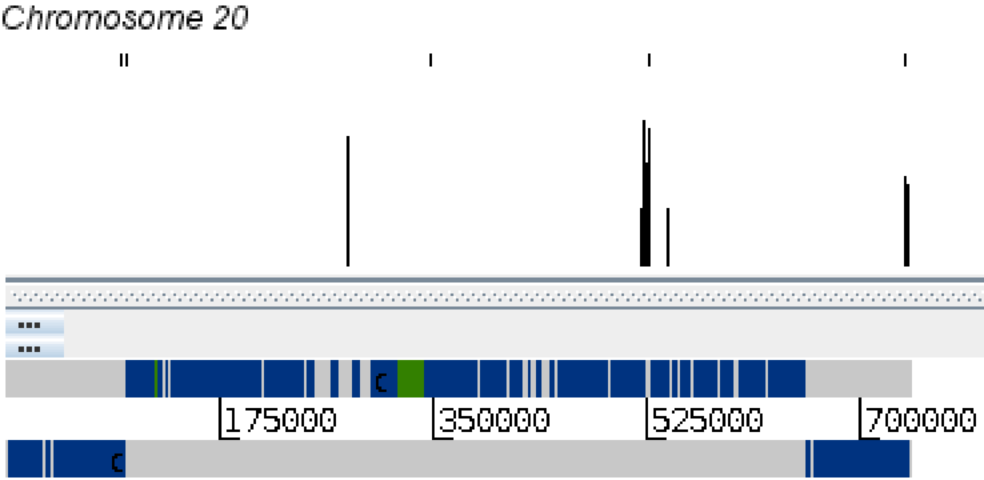
Chromosome 20
175000
350000
525000
700000

Chromosome 21

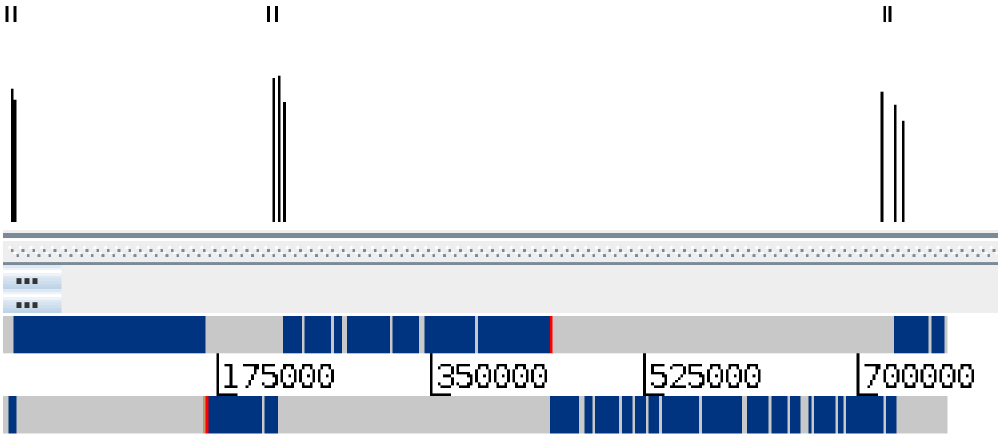

Chromosome 22

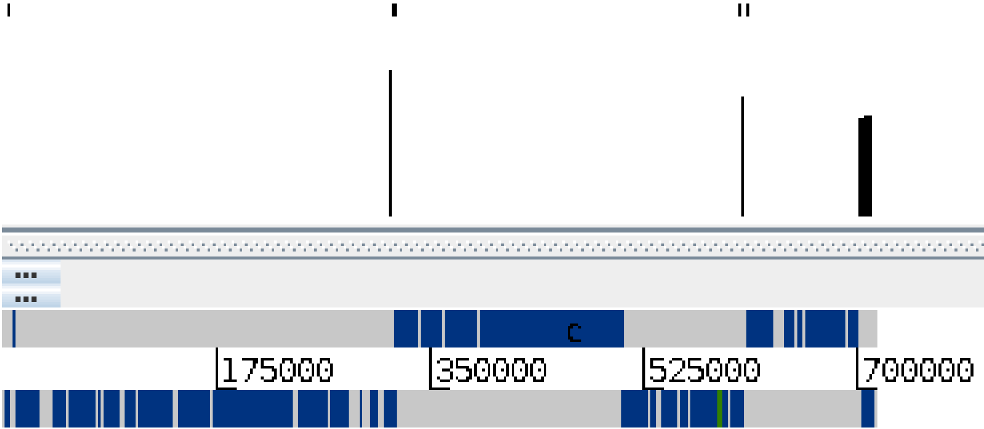

Chromosome 23

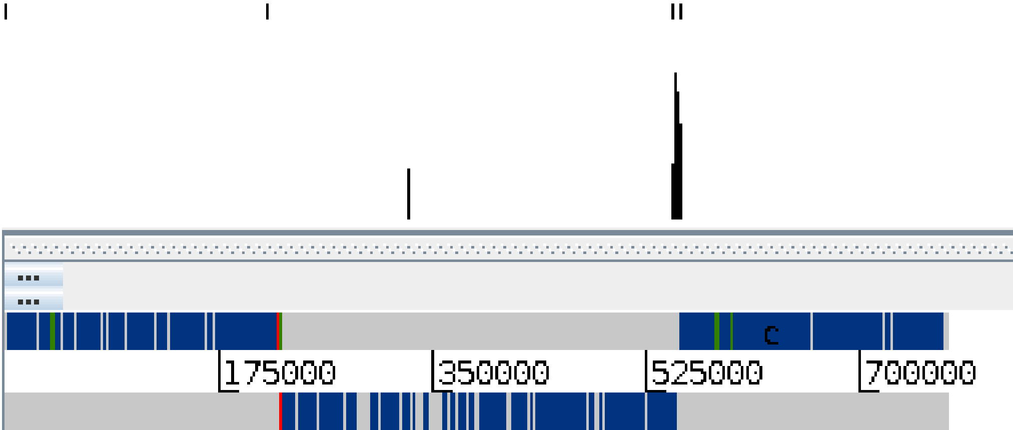

Chromosome 24

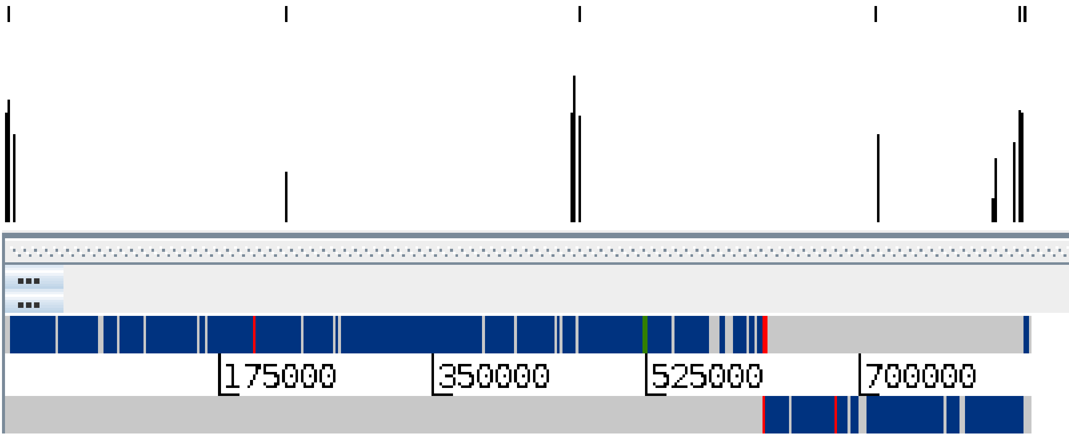

Chromosome 25

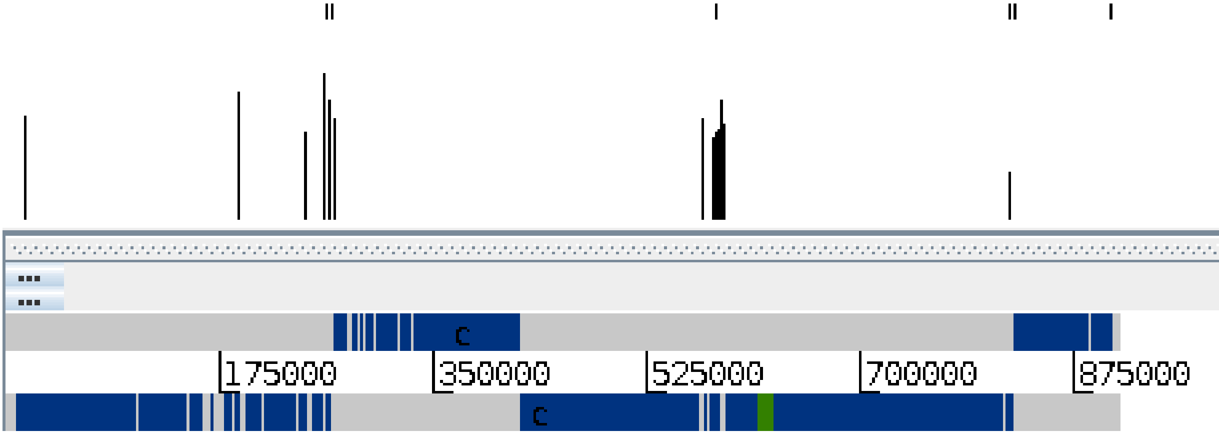

Chromosome 26

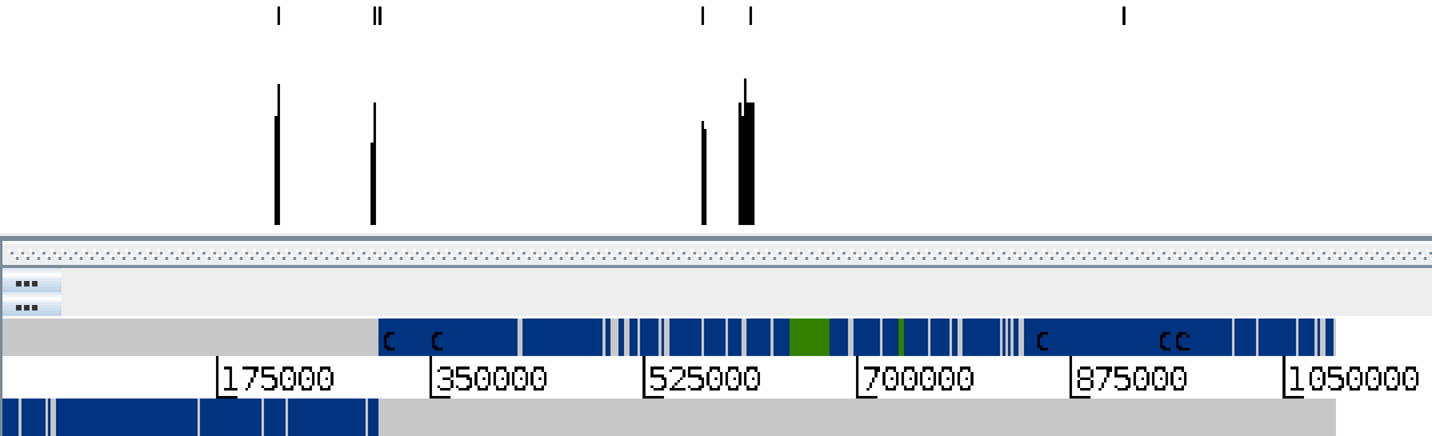

Chromosome 27

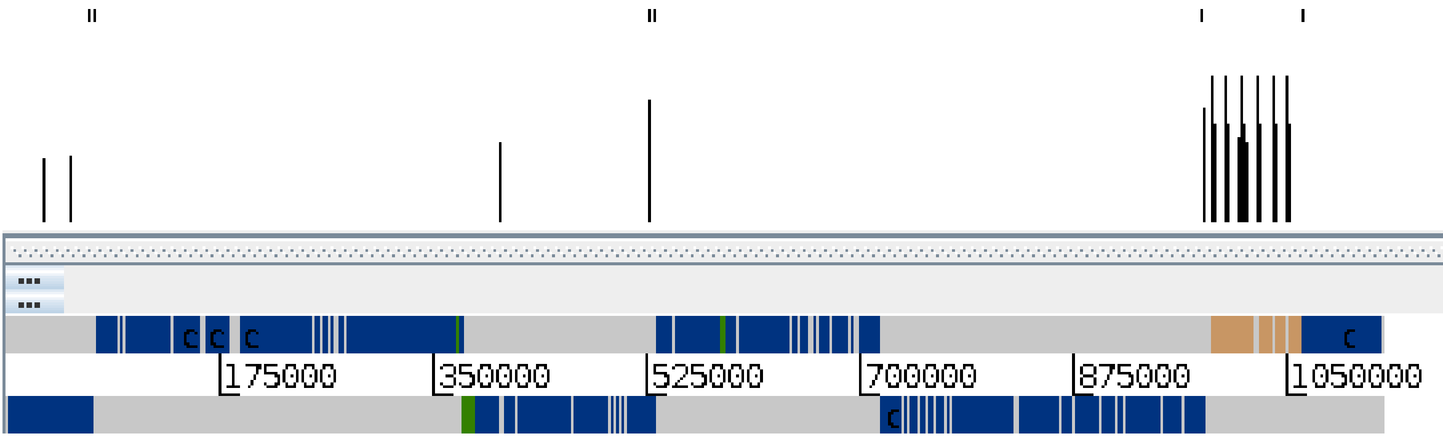

Chromosome 28

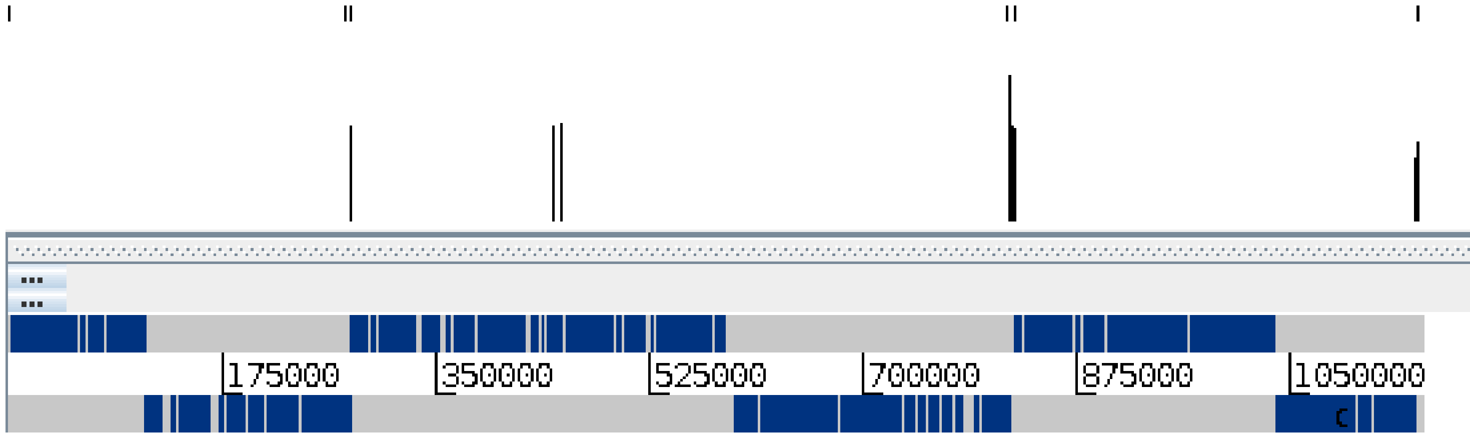

Chromosome 29

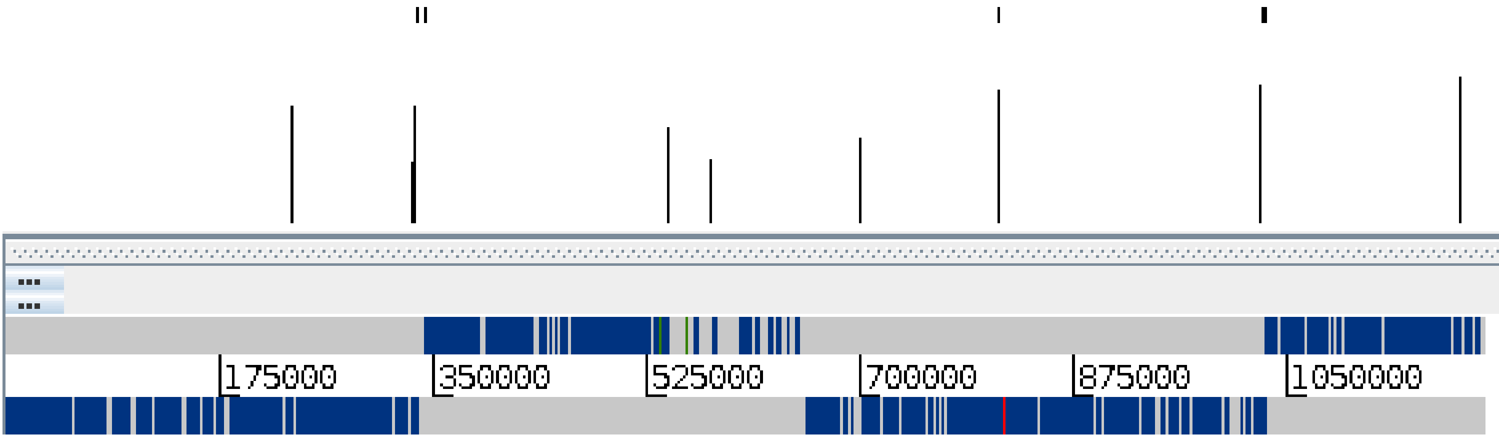

Chromosome 30

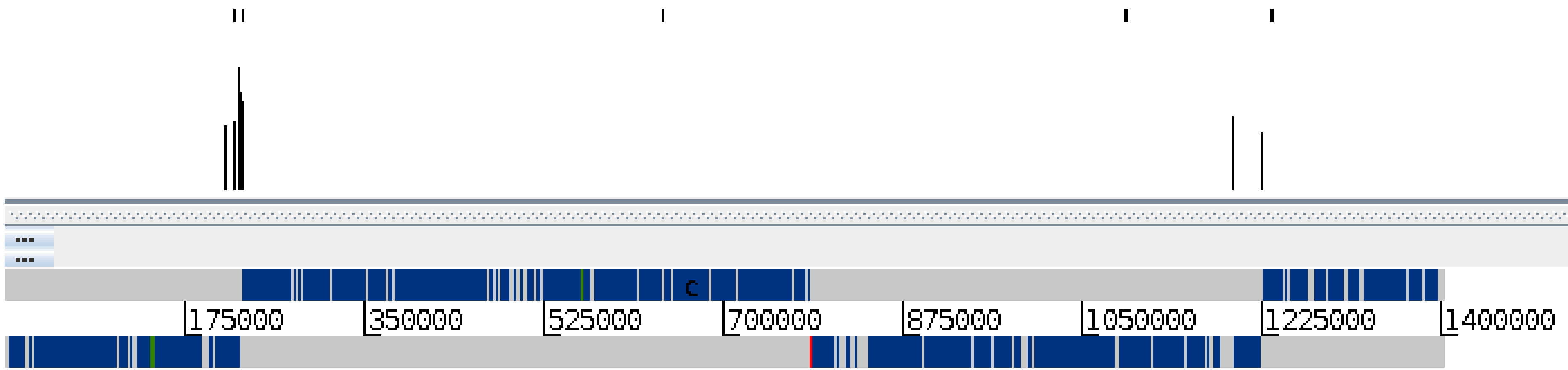

Chromosome 31

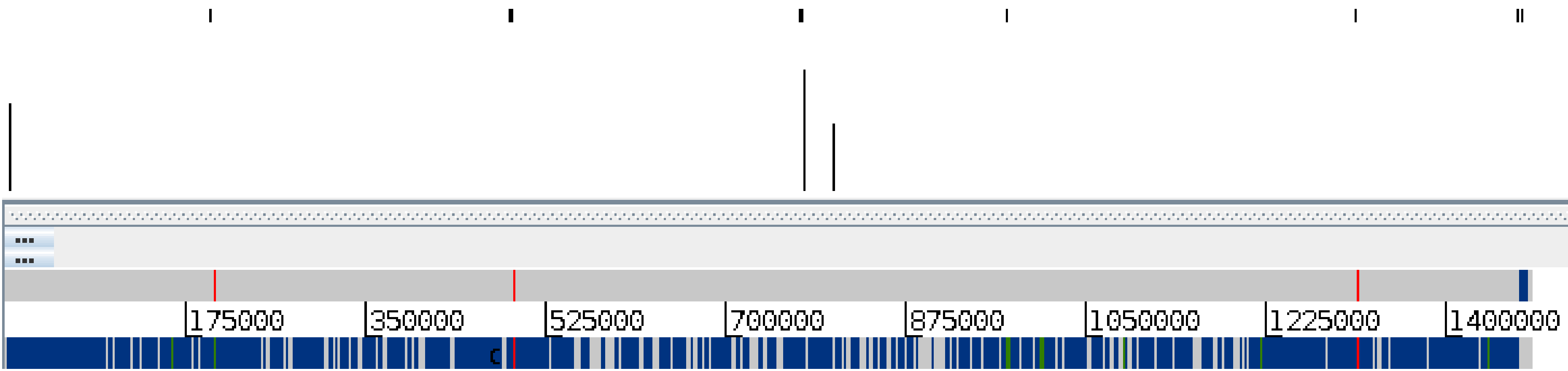

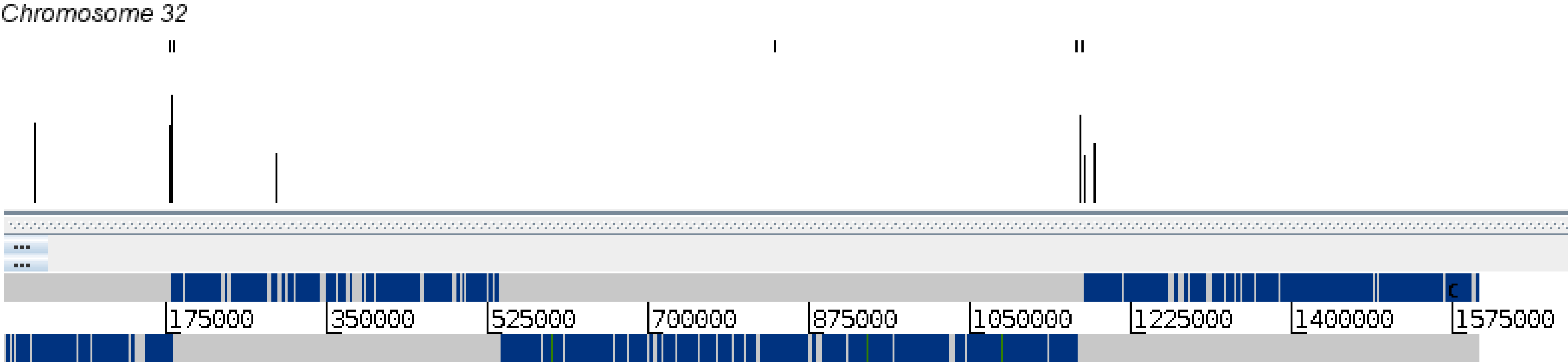
Chromosome 32
175000
350000
525000
700000
875000
1050000
1225000
1400000
1575000

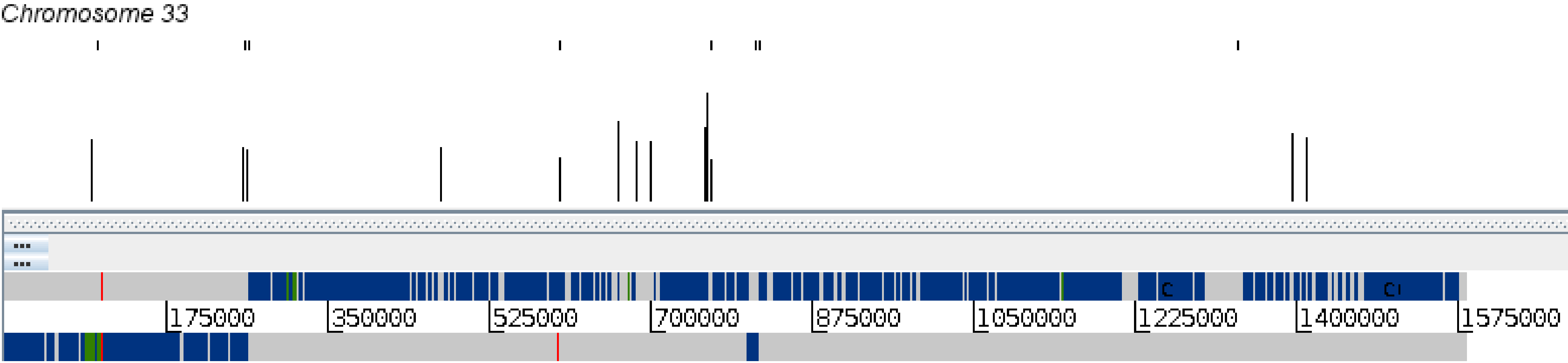

Chromosome 33
175000
350000
525000
700000
875000
1050000
1225000
1400000
1575000

Chromosome 34

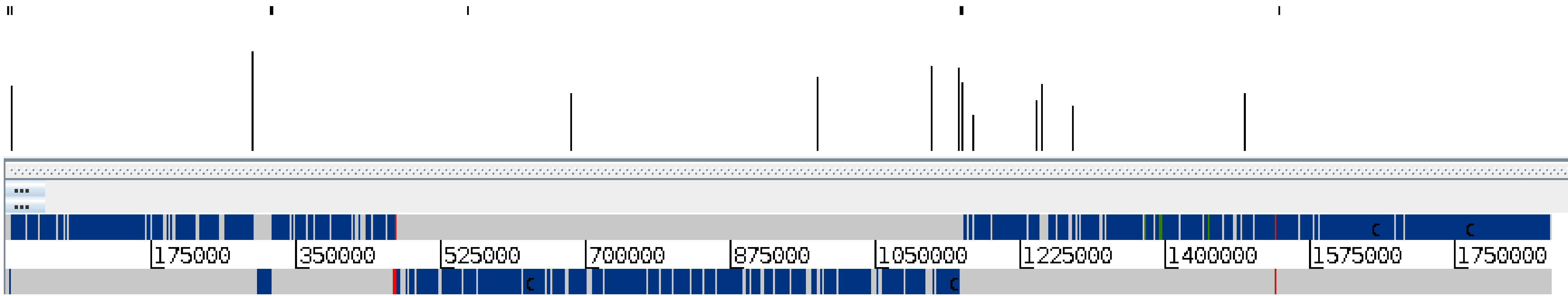

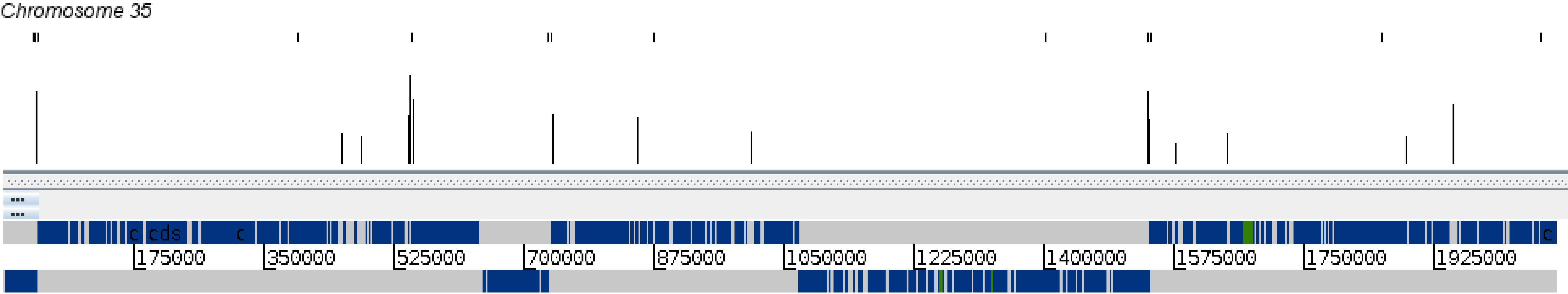
Chromosome 35
c cds
c
175000
350000
525000
700000
875000
1050000
1225000
1400000
1575000
1750000
1925000
c

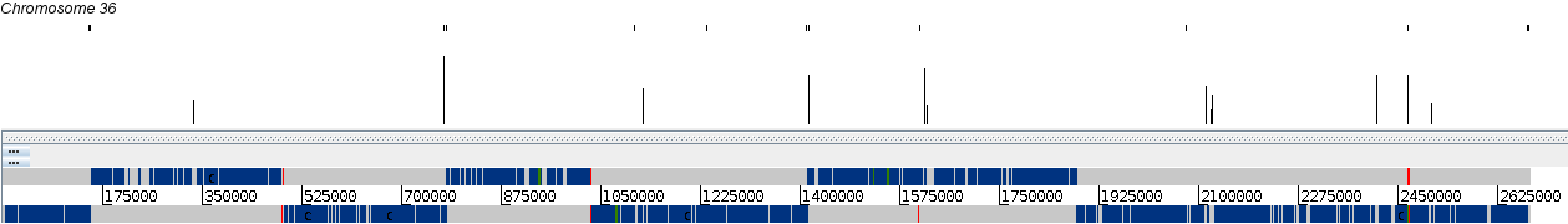
Chromosome 36
175000
350000
525000
700000
875000
1050000
1225000
1400000
1575000
1750000
1925000
2100000
2275000
2450000
2625000
