# Supplementary material for: Genomic Analysis of Sequence-Dependent DNA Curvature in Leishmania
Source: PLoS One. 2013 Apr 30;8(4):e63068. doi: 10.1371/journal.pone.0063068 (PMC3639952; doi:10.1371/journal.pone.0063068)
Supplement: Figure S7 — Location conservation of high RIIC scoring regions in L. major and L. infantum chromosomes. The graphs are the same as figure 1. Blast HSPs longer than 100 bp and with at least 80% similarity are displayed in red scale and blue lines represent inversions. (PDF) [file pone.0063068.s007.pdf]

S Figure 7. Location conservation of high RIIC scoring regions in *L. major* and *L. infantum* chromosomes

The graphs are the same as figure 1. Blast HSPs longer than 100bp and with at least 80% similarity are displayed in red scale and blue lines represent inversions.

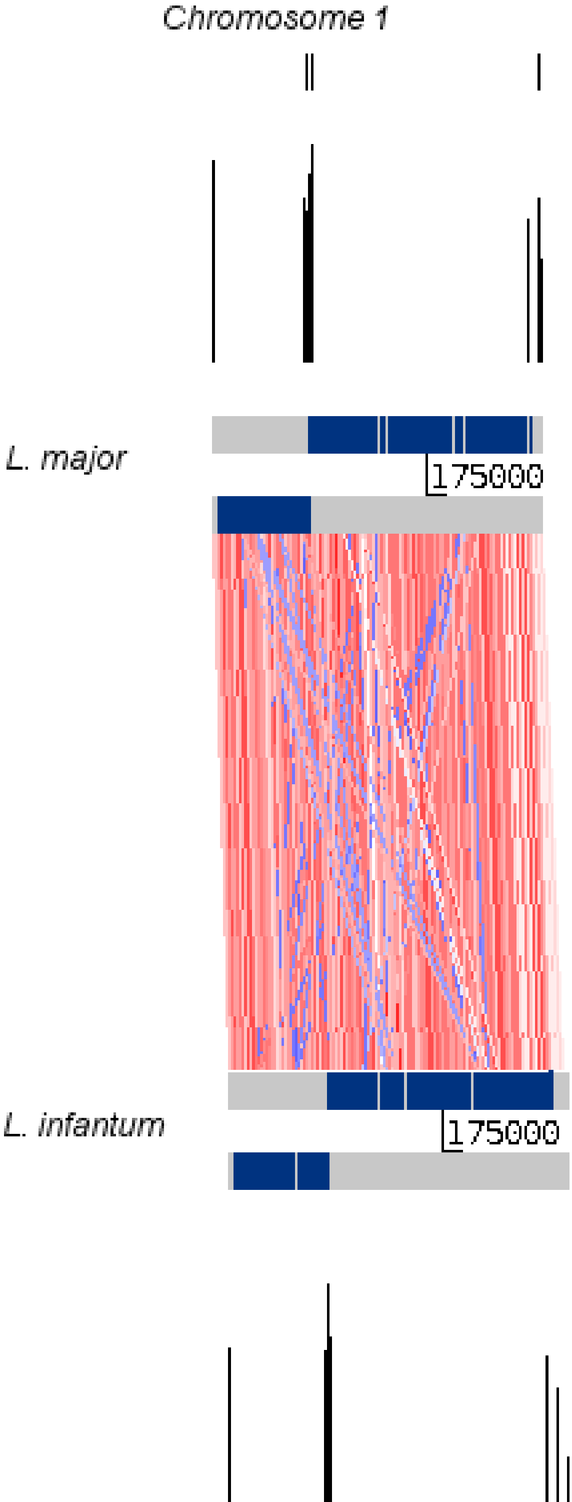

Chromosome 1
L. major
175000
L. infantum
175000

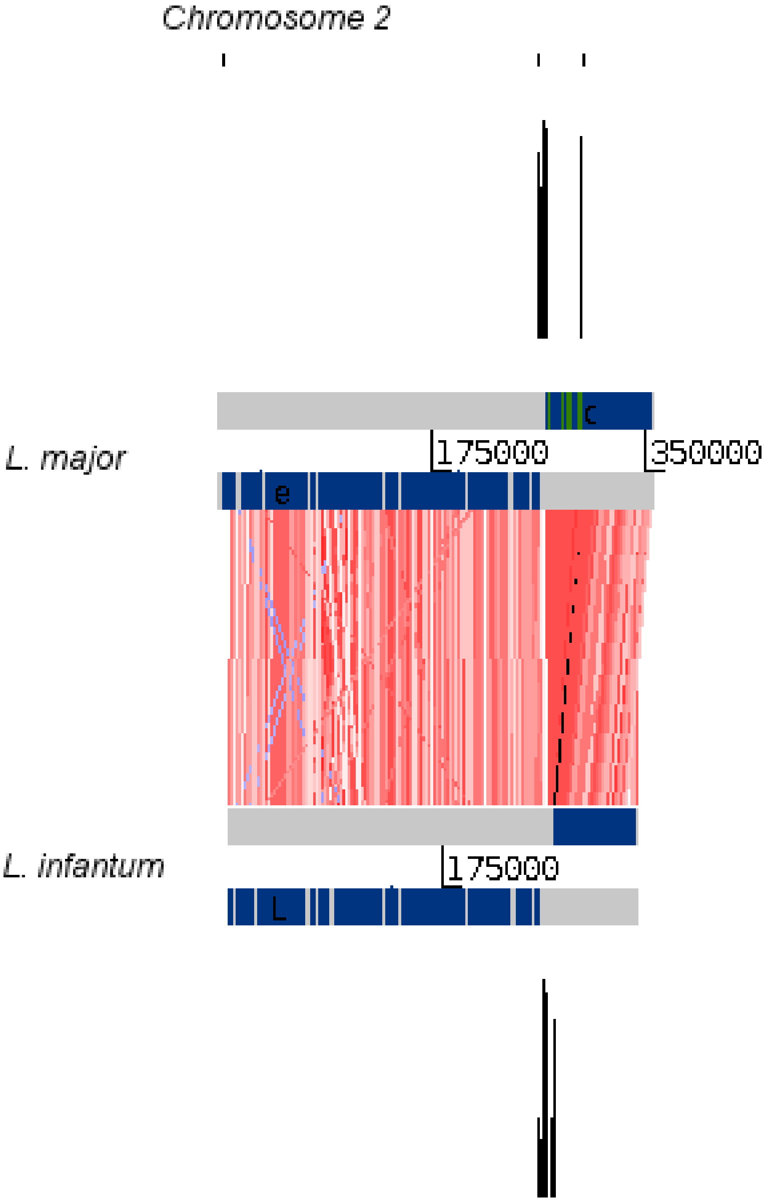

Chromosome 2
L. major
c
175000
350000
e
L. infantum
175000
L

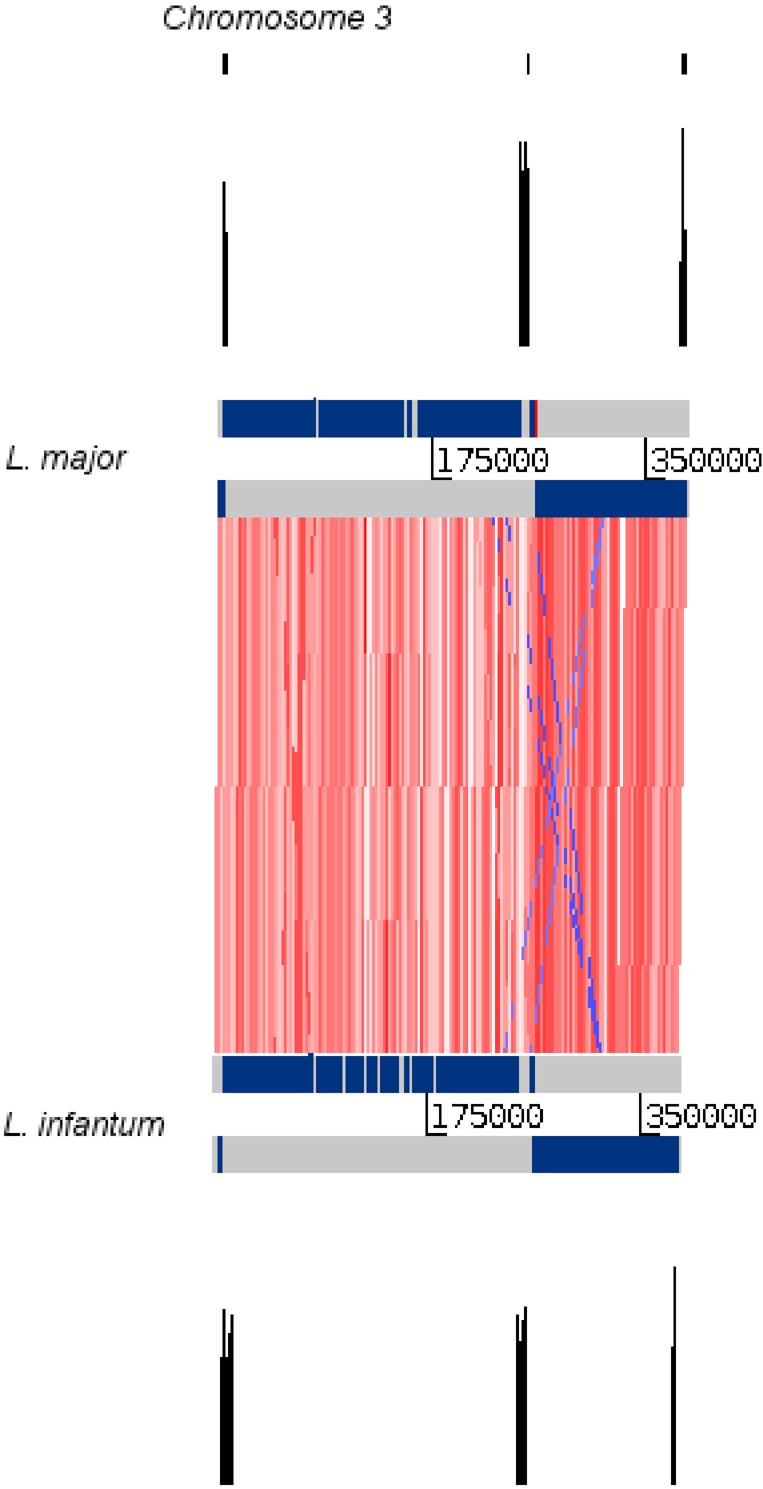
Chromosome 3
L. major
175000
350000
L. infantum
175000
350000

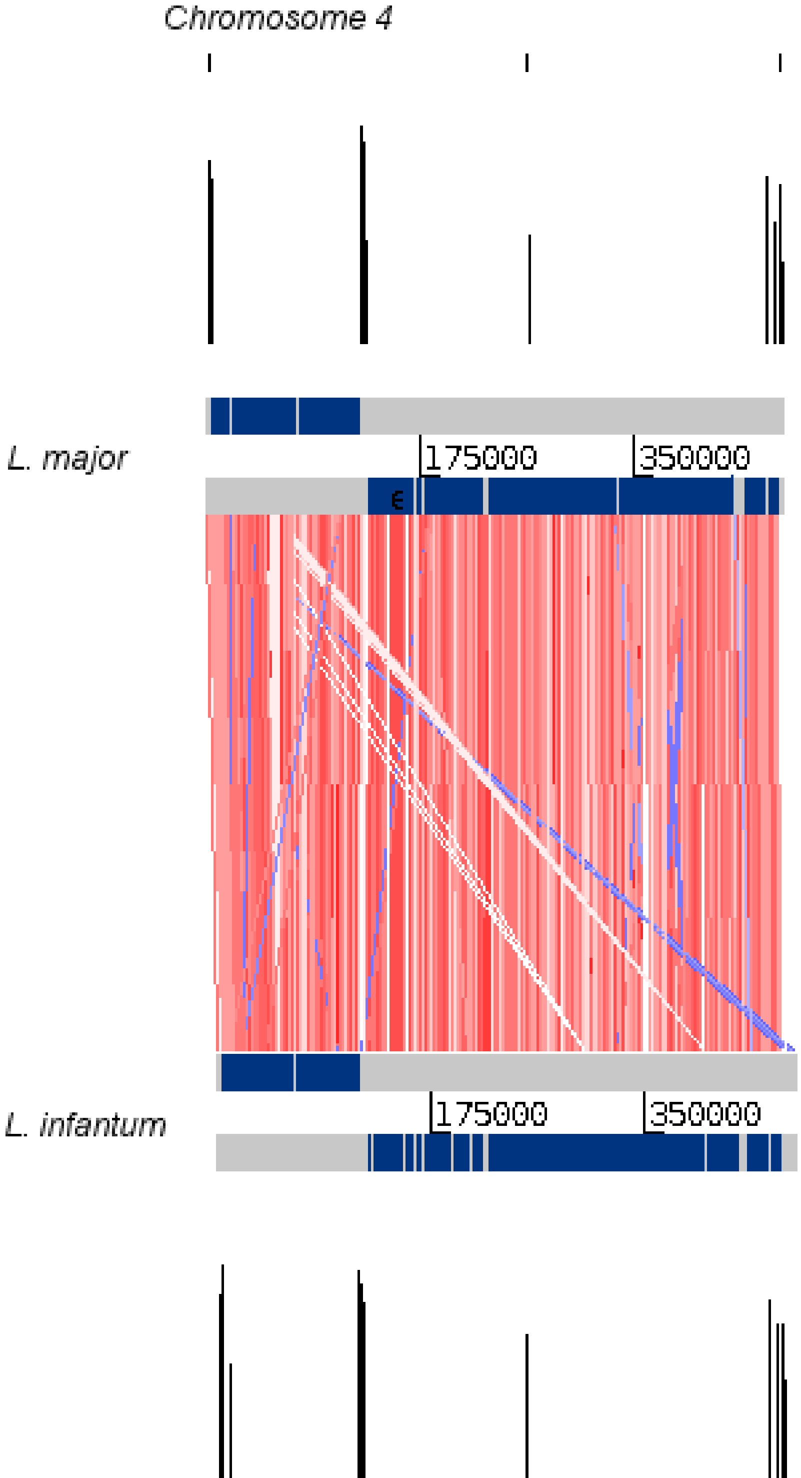

Chromosome 4
L. major
175000
350000
ε
L. infantum
175000
350000

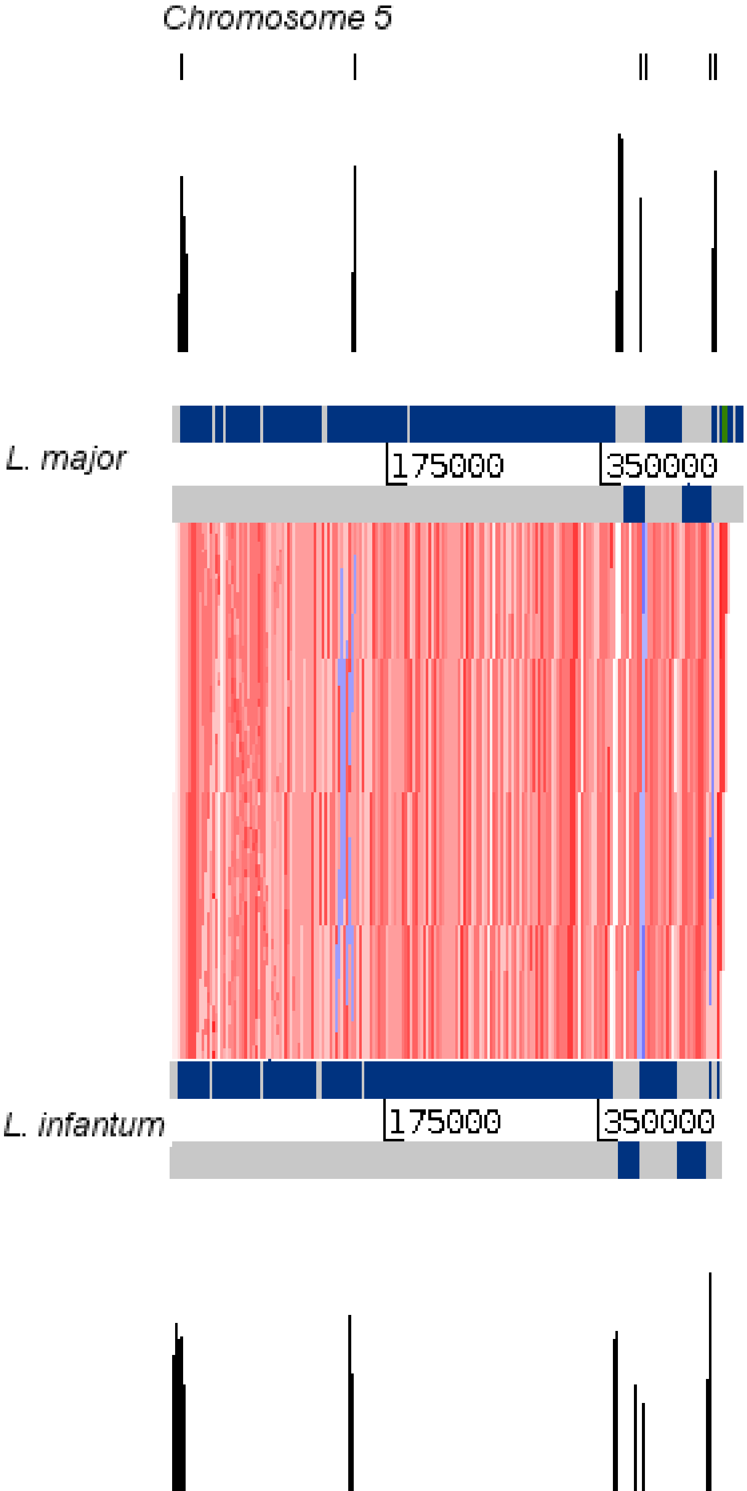

Chromosome 5
L. major
175000
350000
L. infantum
175000
350000

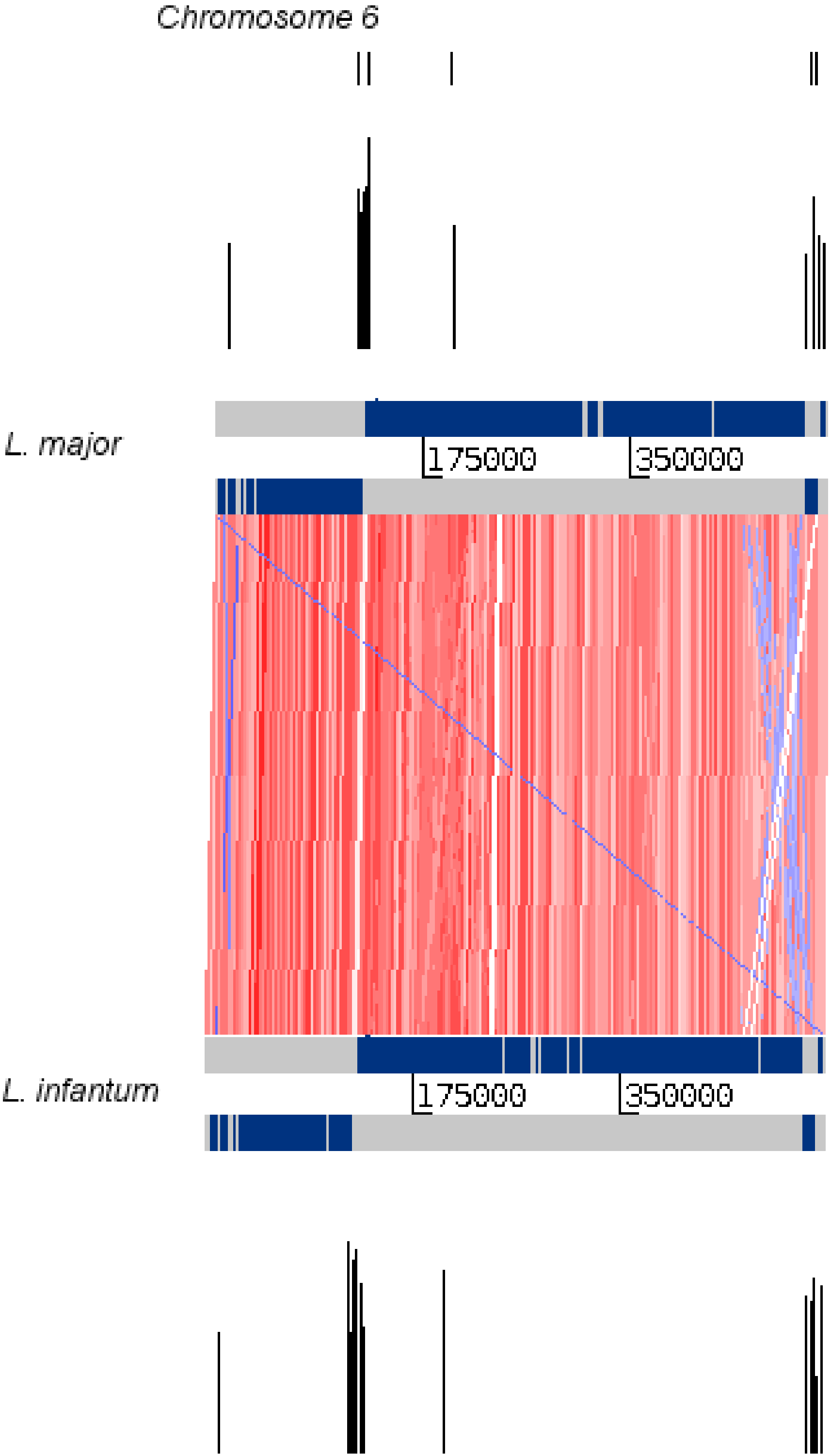
Chromosome 6
L. major
175000
350000
L. infantum
175000
350000

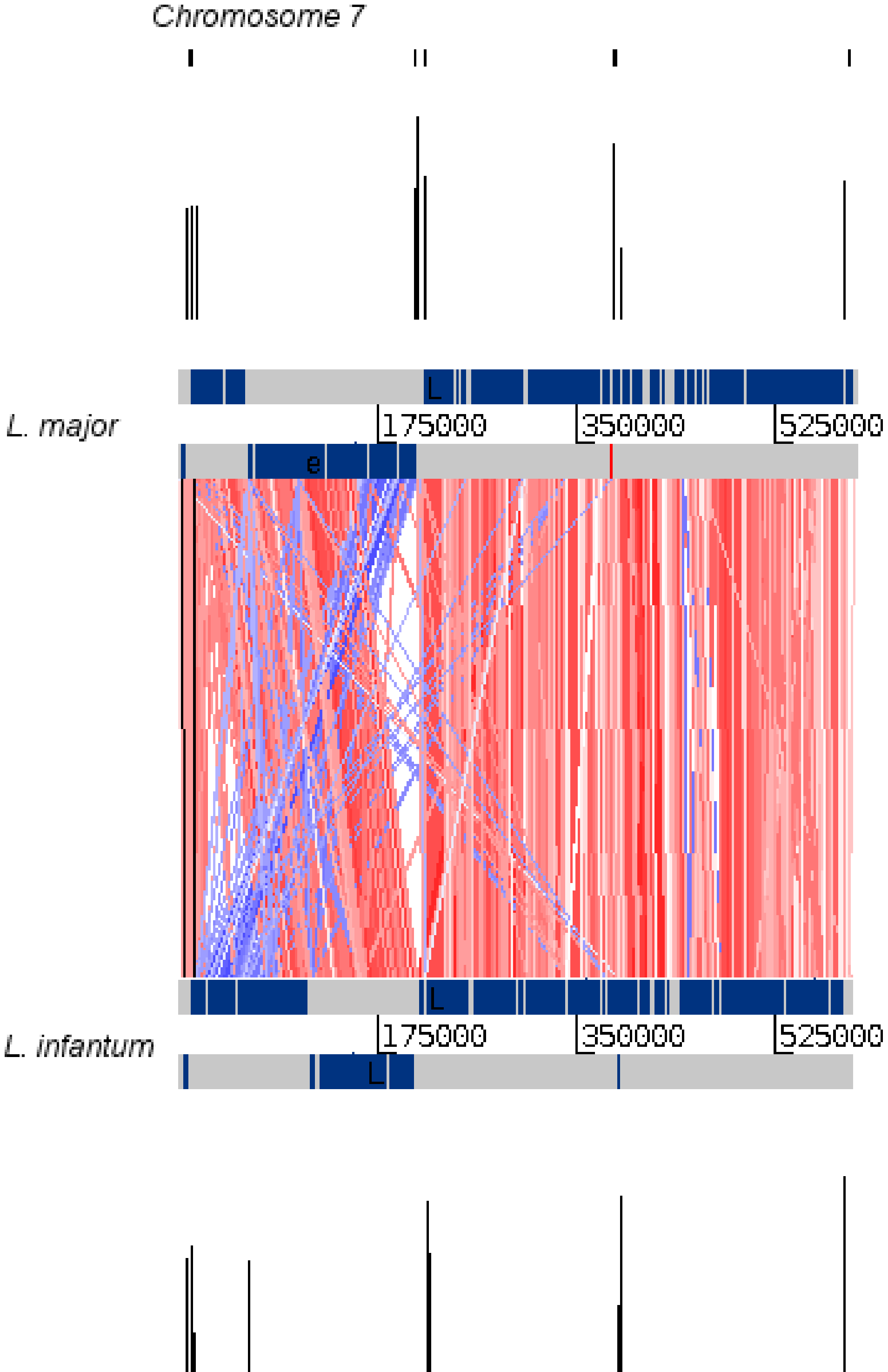

Chromosome 7
L. major
175000
350000
525000
L. infantum
175000
350000
525000

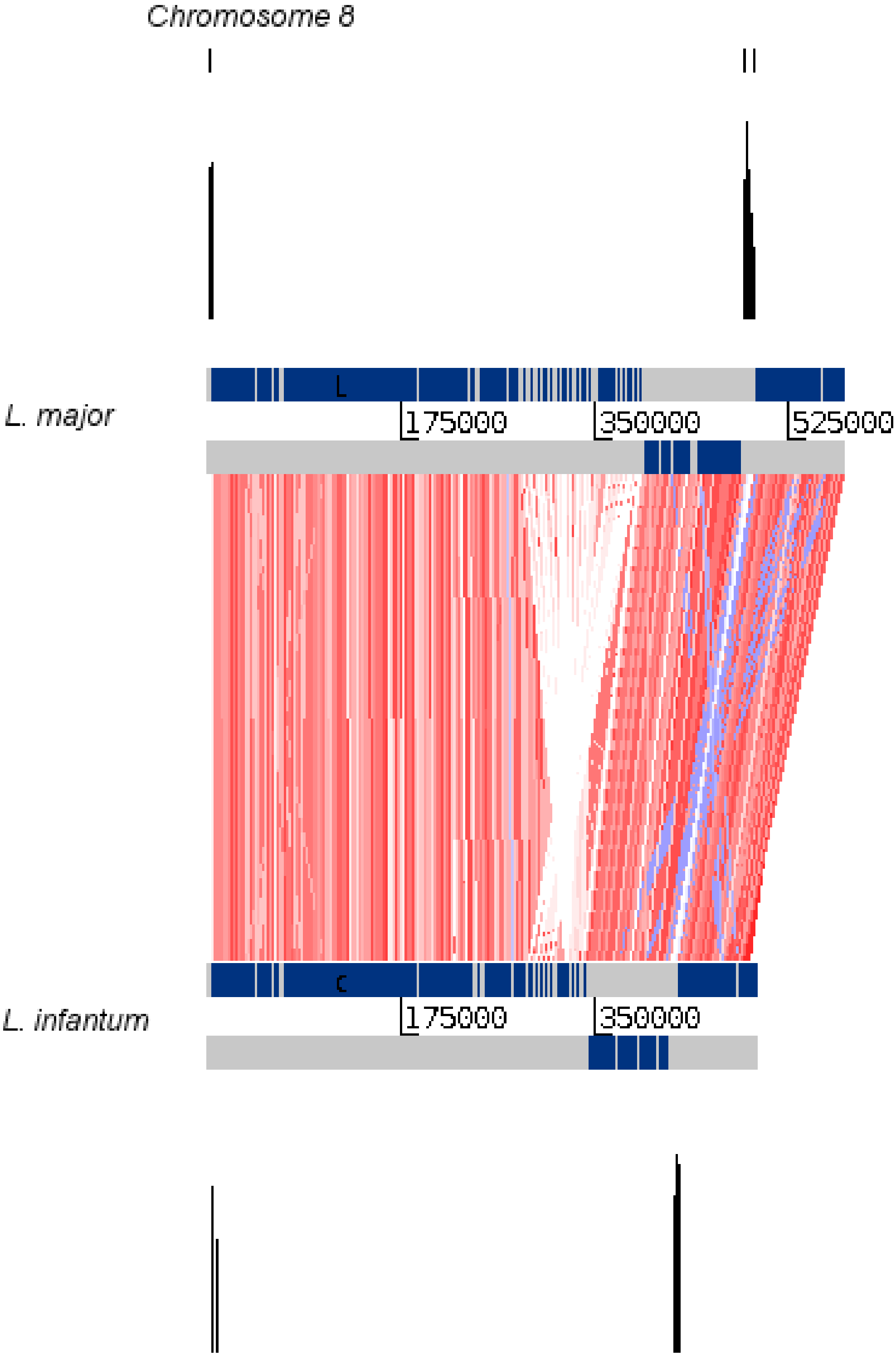

Chromosome 8
I
II
L
L. major
175000
350000
525000
L. infantum
175000
350000

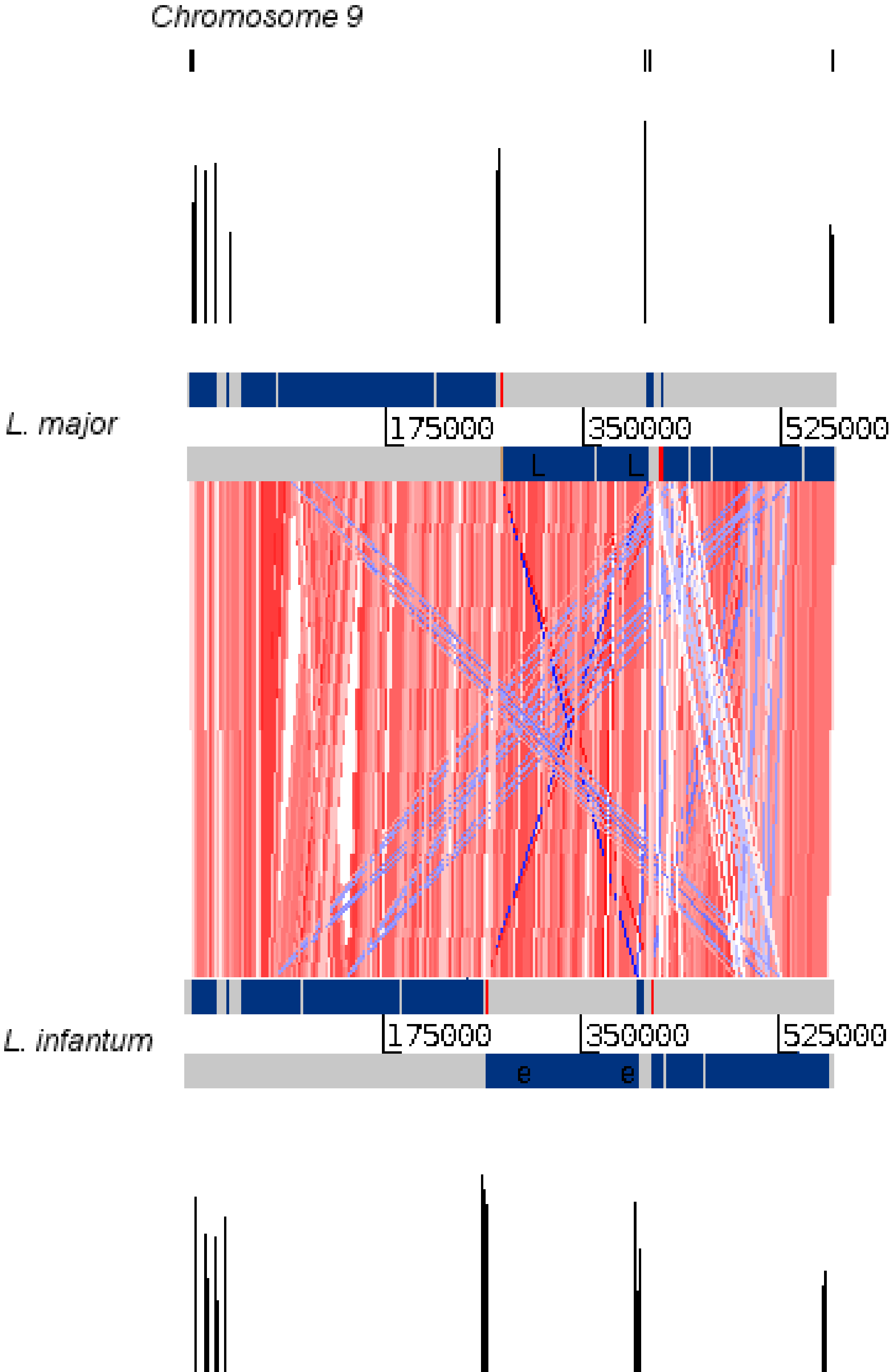

Chromosome 9
L. major
175000
350000
525000
L
L
L. infantum
175000
350000
525000
e
e

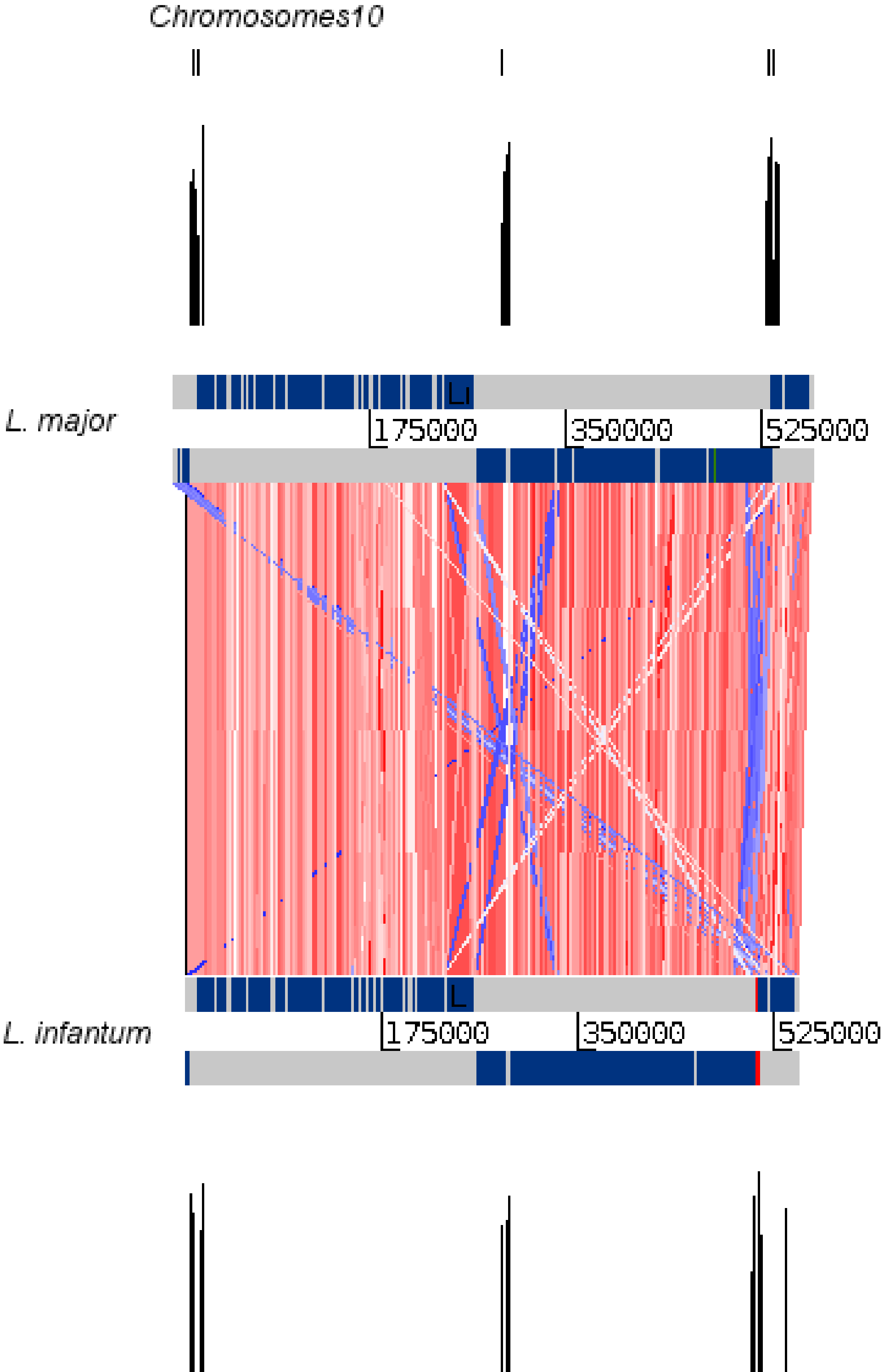

Chromosomes10
L. major
175000
350000
525000
L. infantum
175000
350000
525000

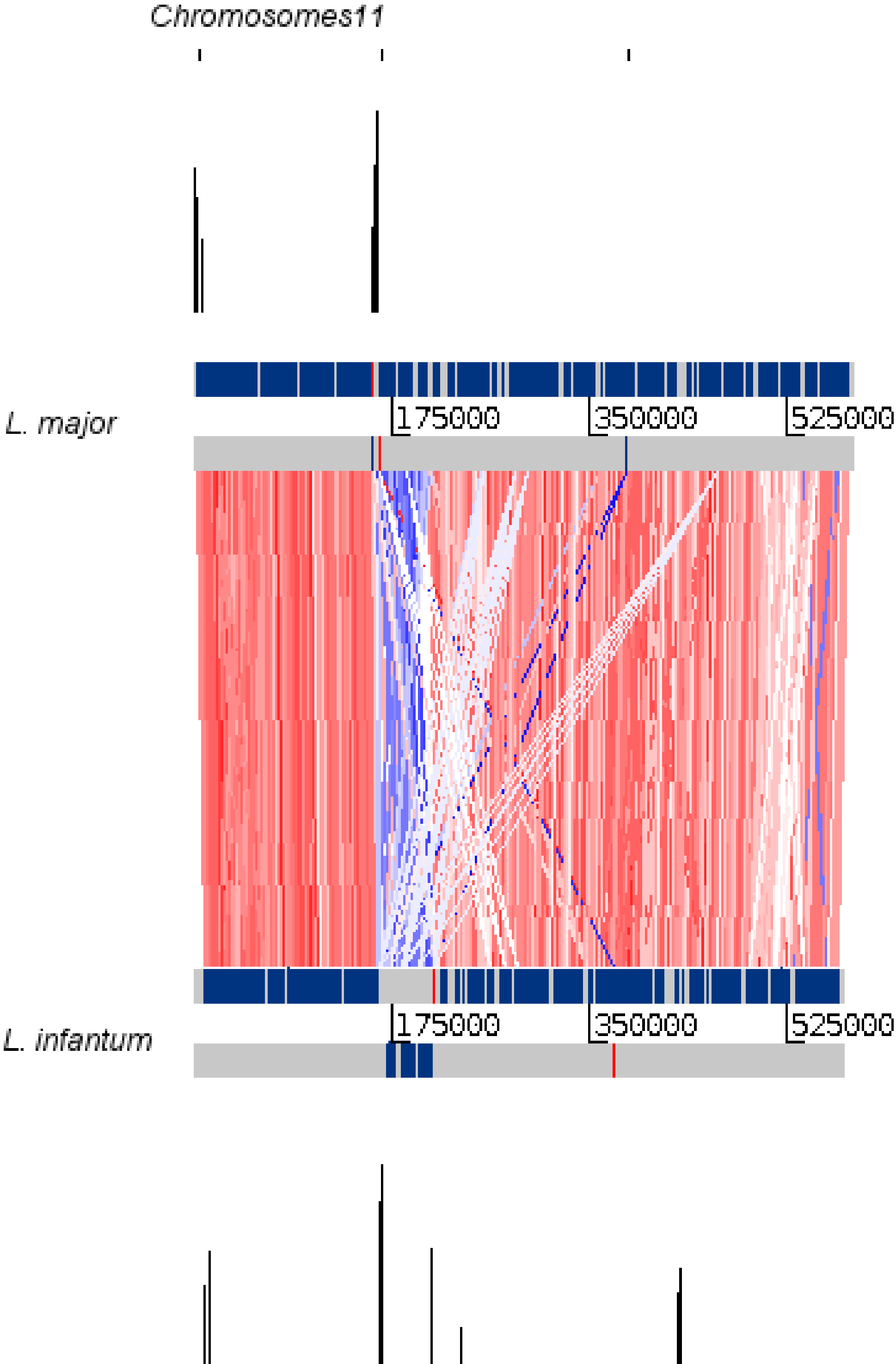
Chromosomes11
L. major
175000
350000
525000
L. infantum
175000
350000
525000

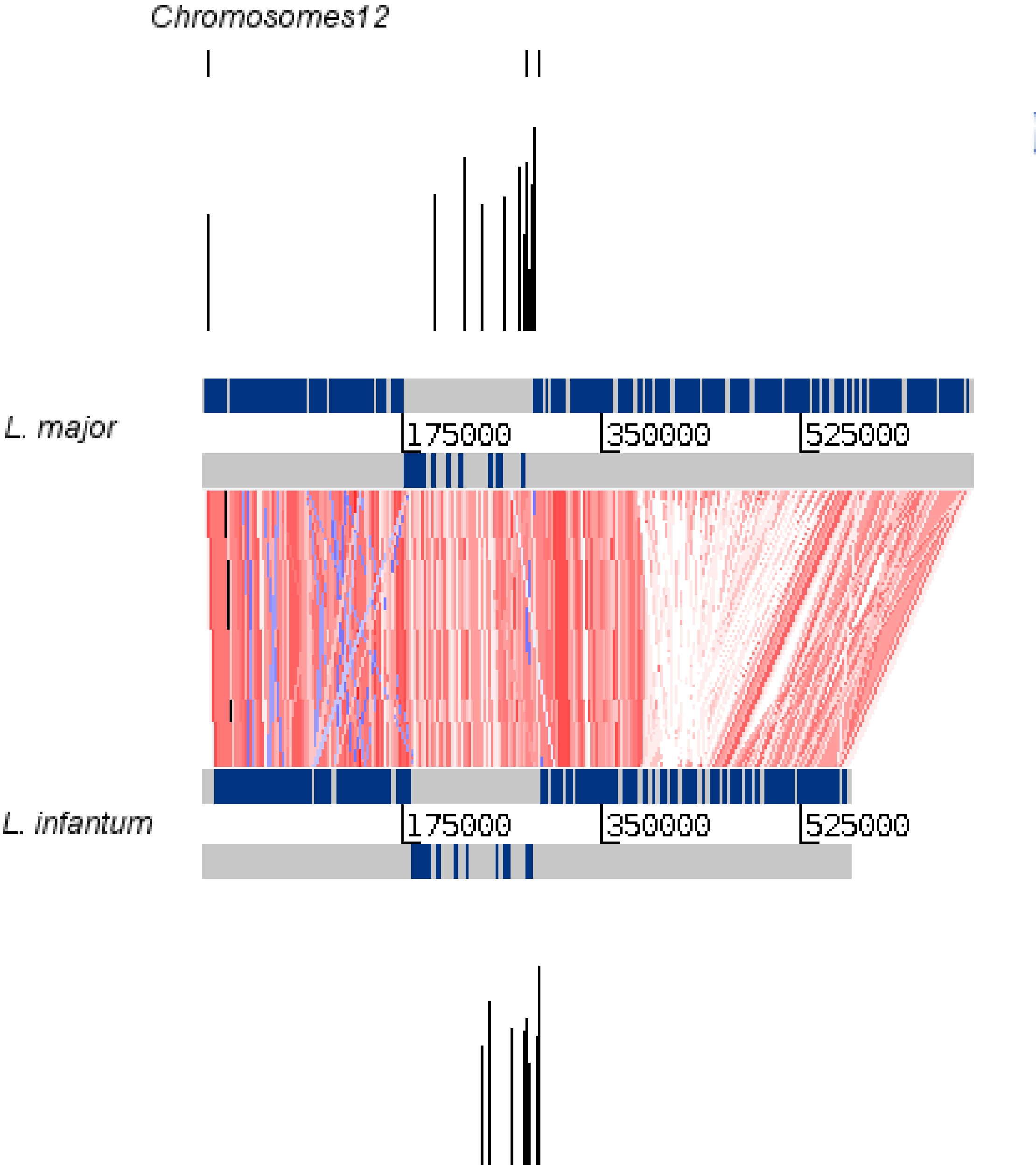

Chromosomes12
L. major
175000
350000
525000
L. infantum
175000
350000
525000

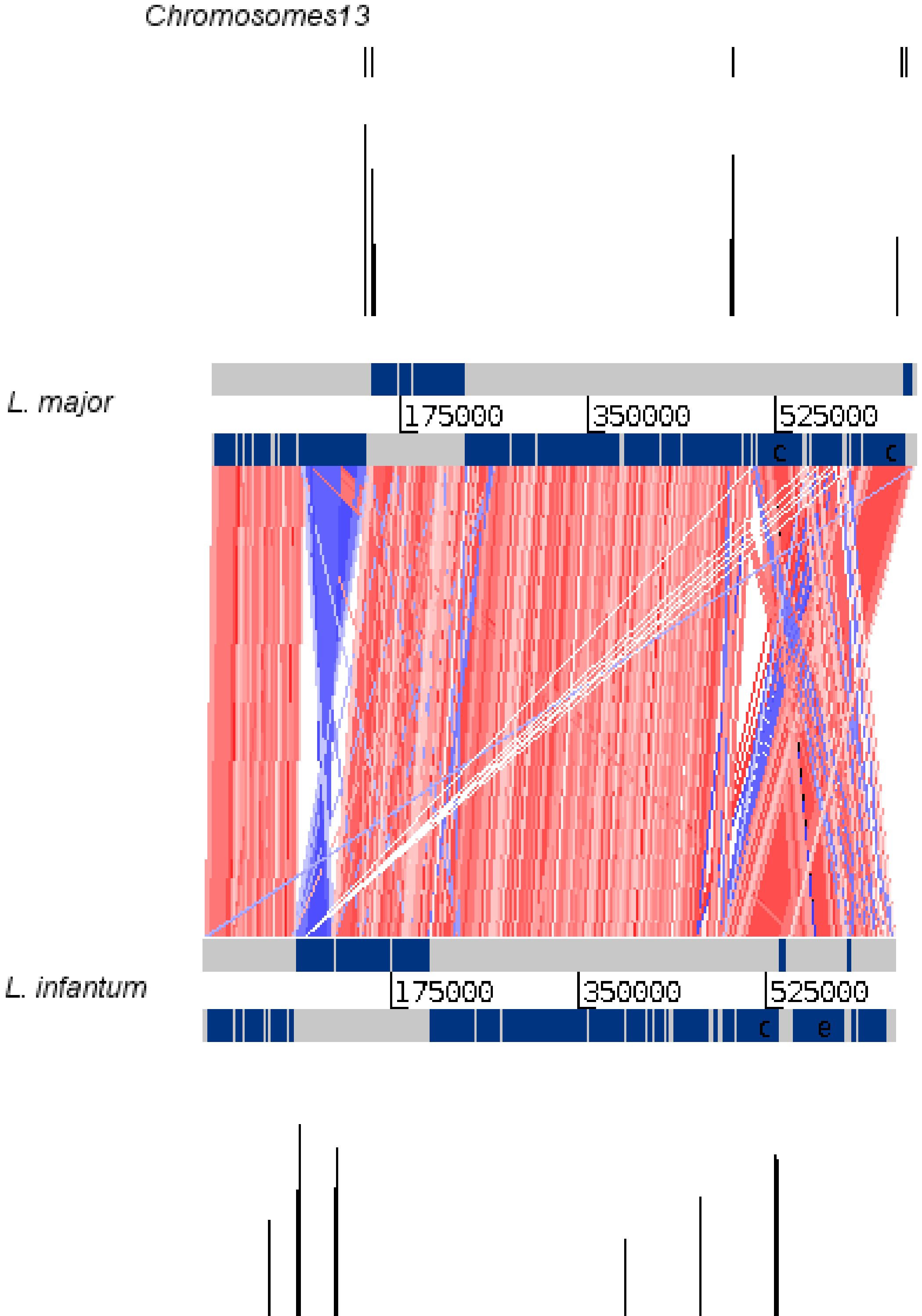

Chromosomes13
L. major
175000
350000
525000
c
c
L. infantum
175000
350000
525000
c
e

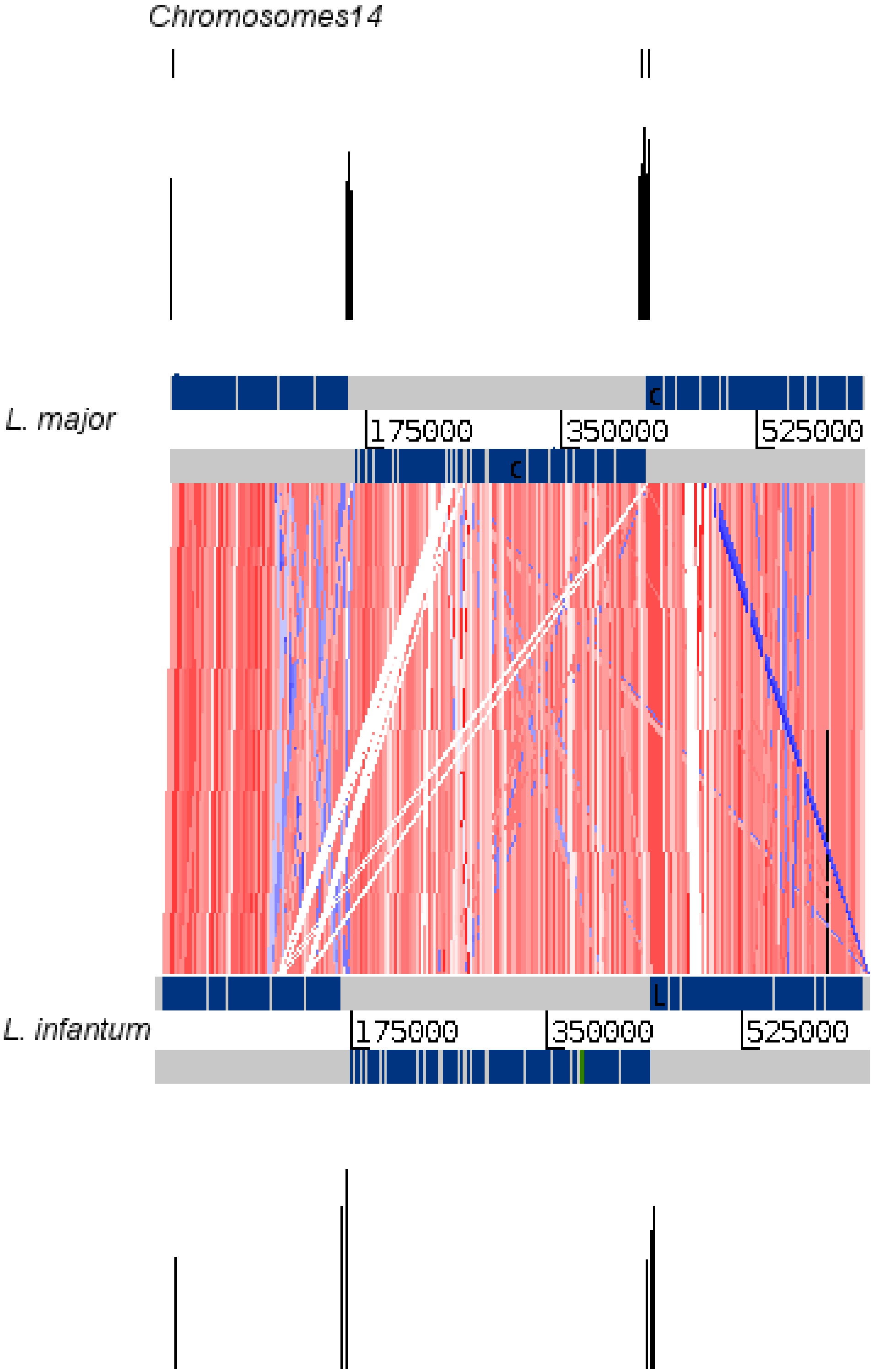
Chromosomes14
L. major
175000
350000
525000
L. infantum
175000
350000
525000

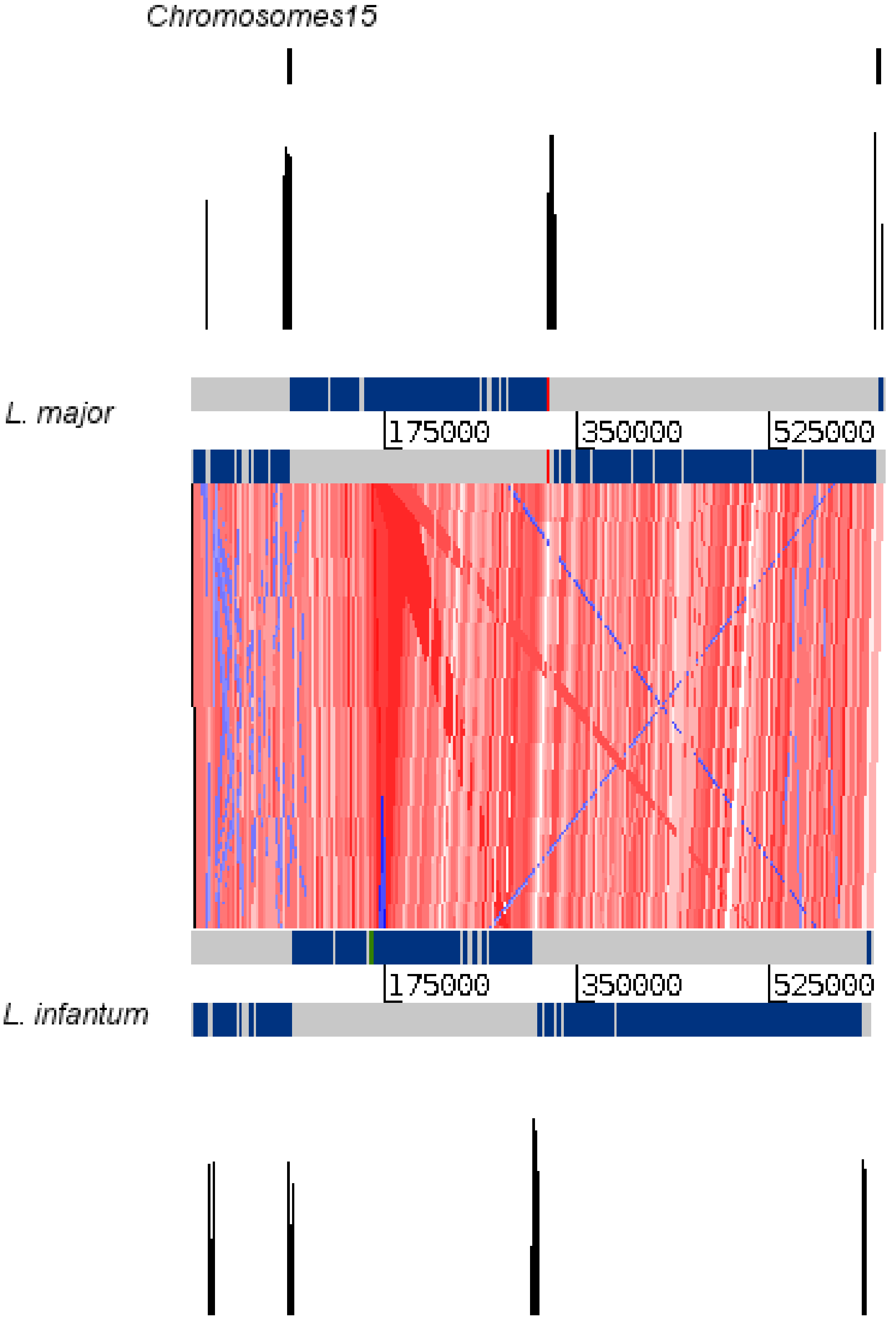
Chromosomes15
L. major
175000
350000
525000
175000
350000
525000
L. infantum

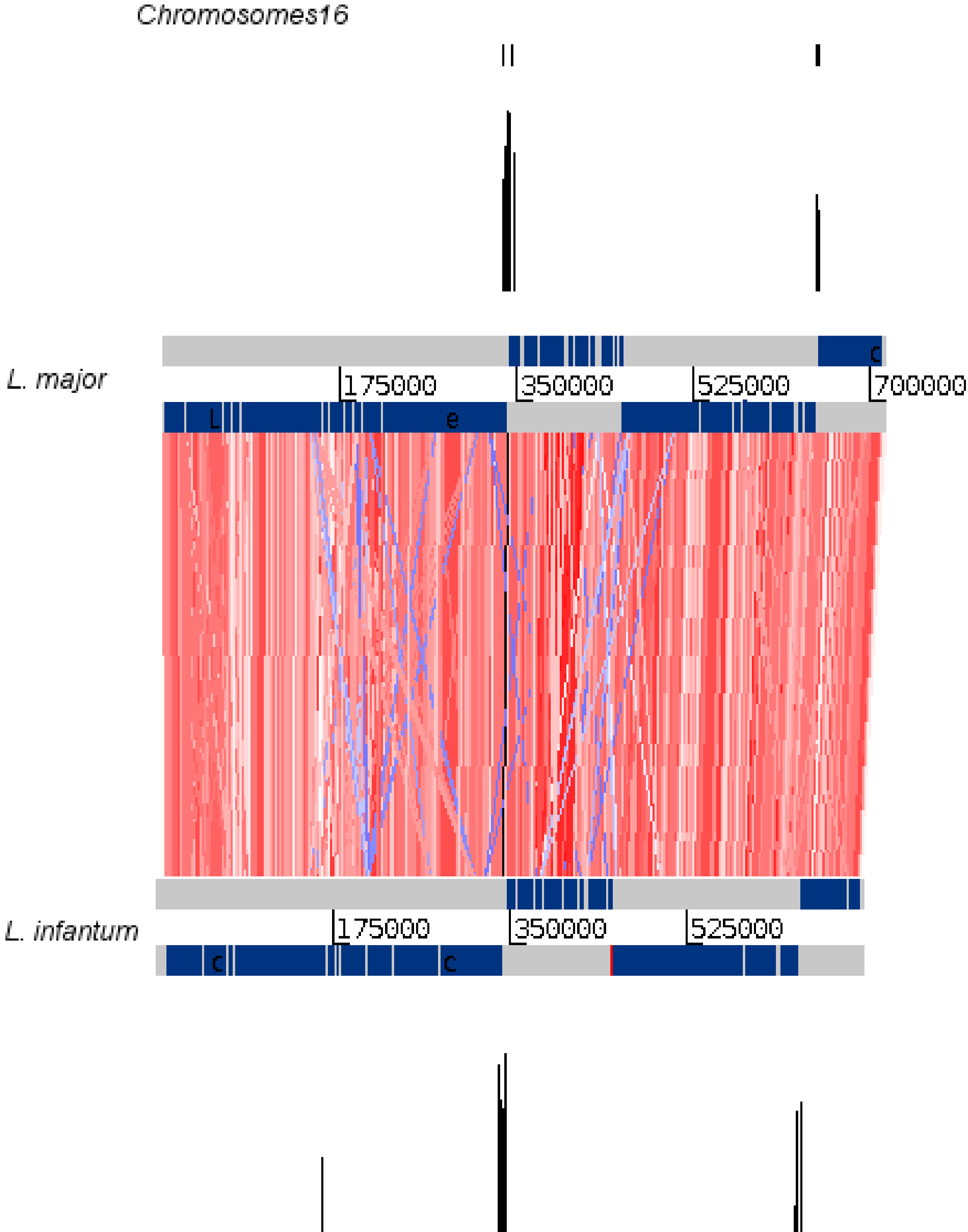
Chromosomes16
L. major
175000
350000
525000
700000
L. infantum
175000
350000
525000

Chromosomes17

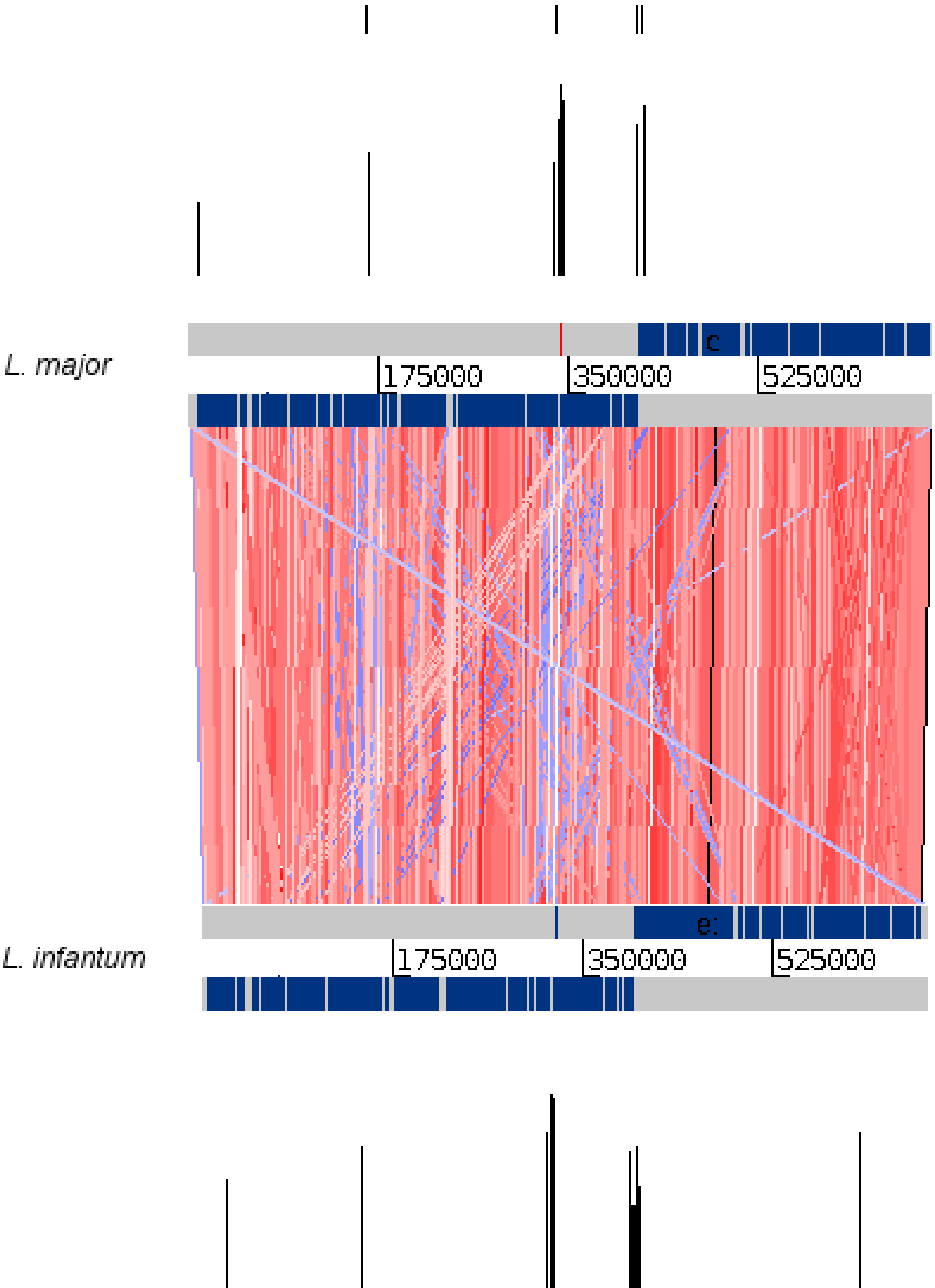

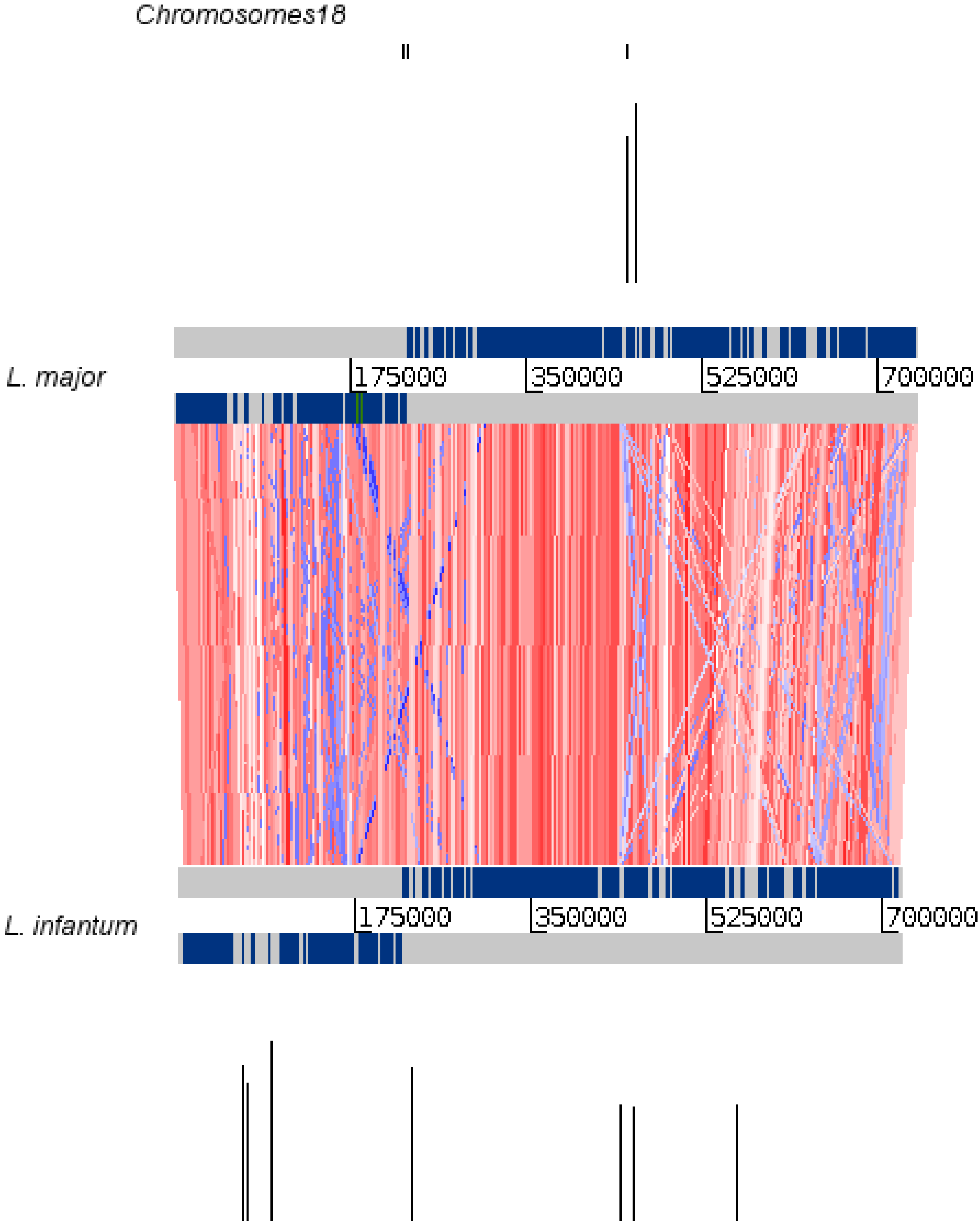

Chromosomes18
II
I
L. major
175000
350000
525000
700000
L. infantum
175000
350000
525000
700000

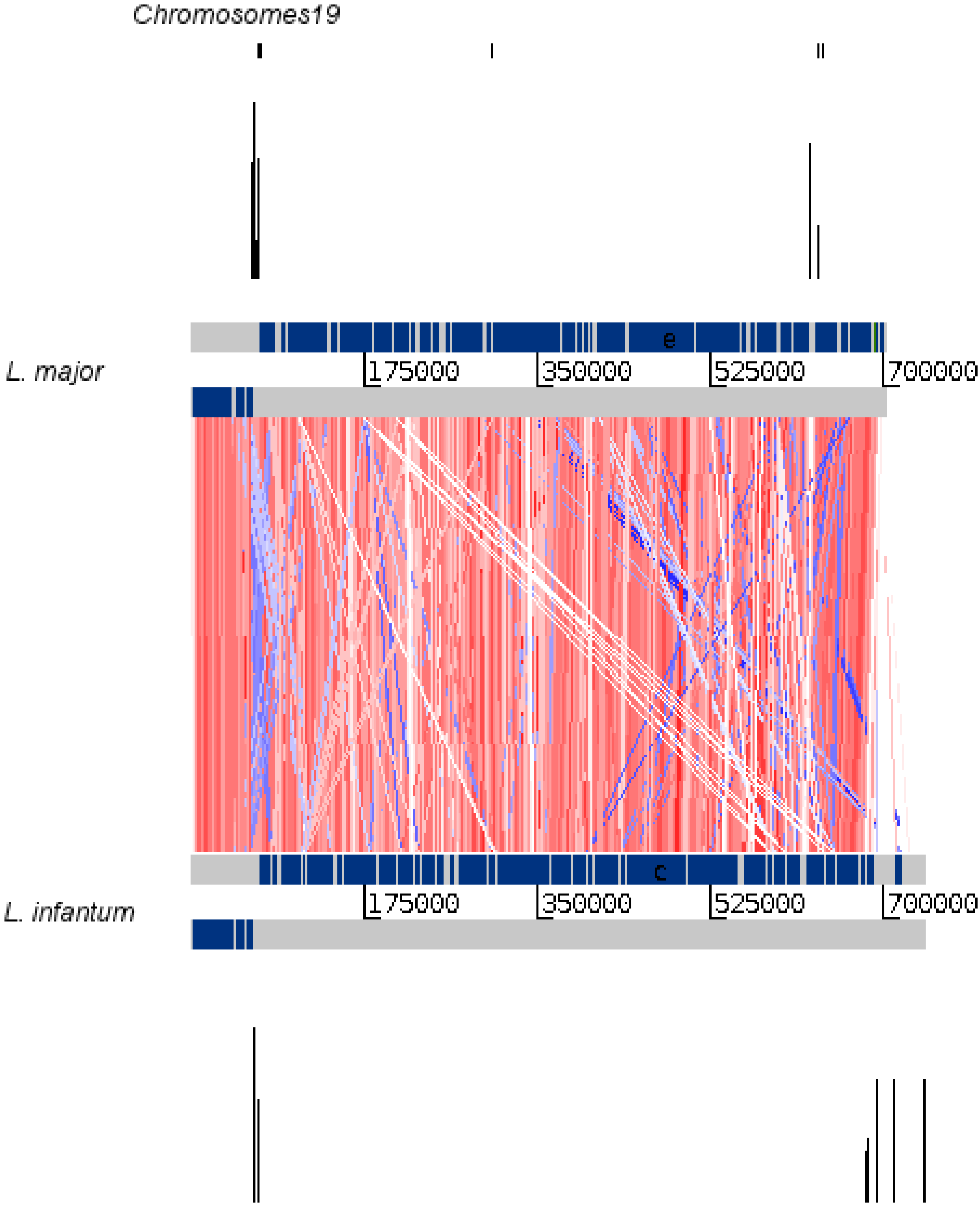
Chromosomes19
e
L. major
175000
350000
525000
700000
c
L. infantum
175000
350000
525000
700000

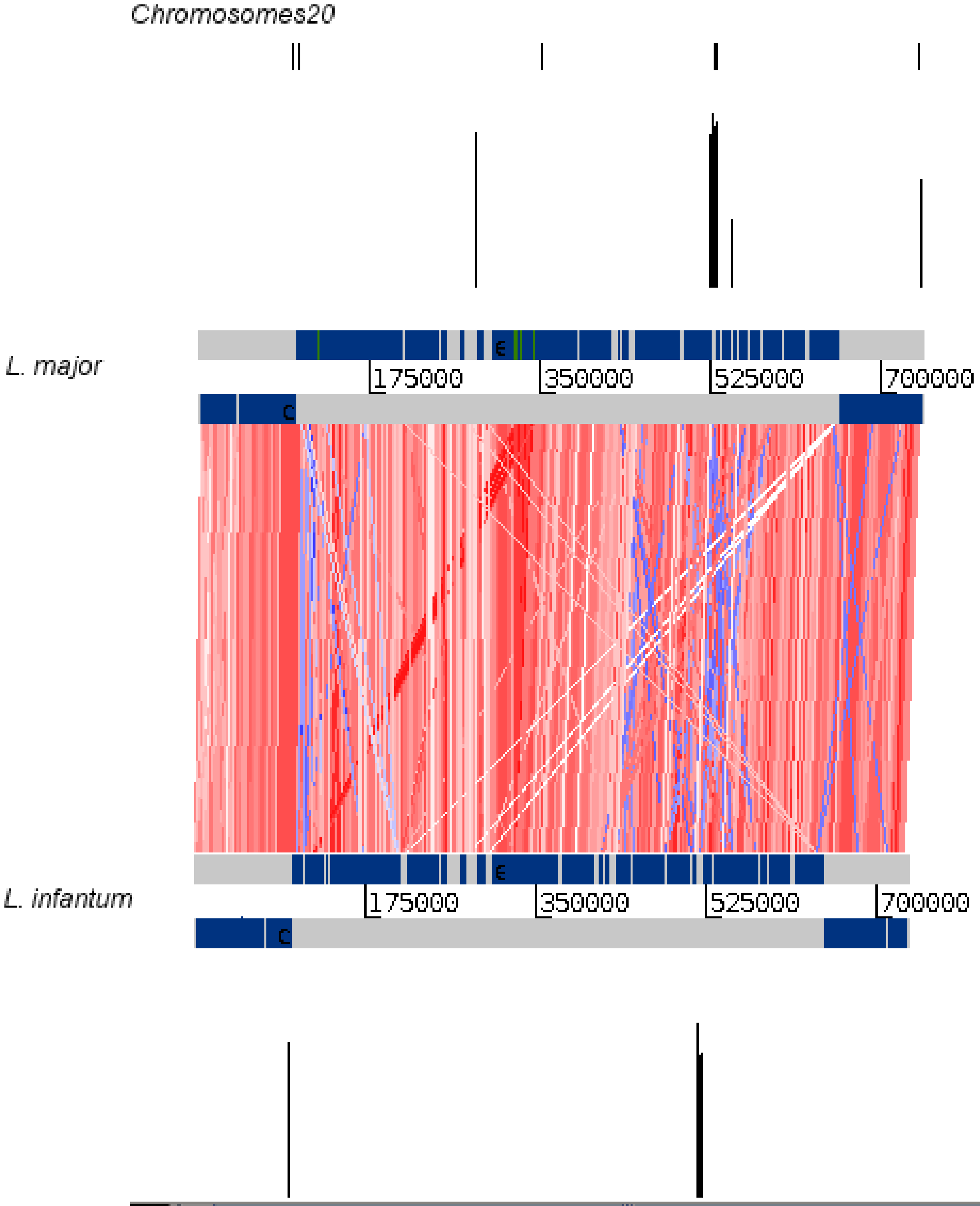
Chromosomes20
L. major
175000
350000
525000
700000
L. infantum
175000
350000
525000
700000

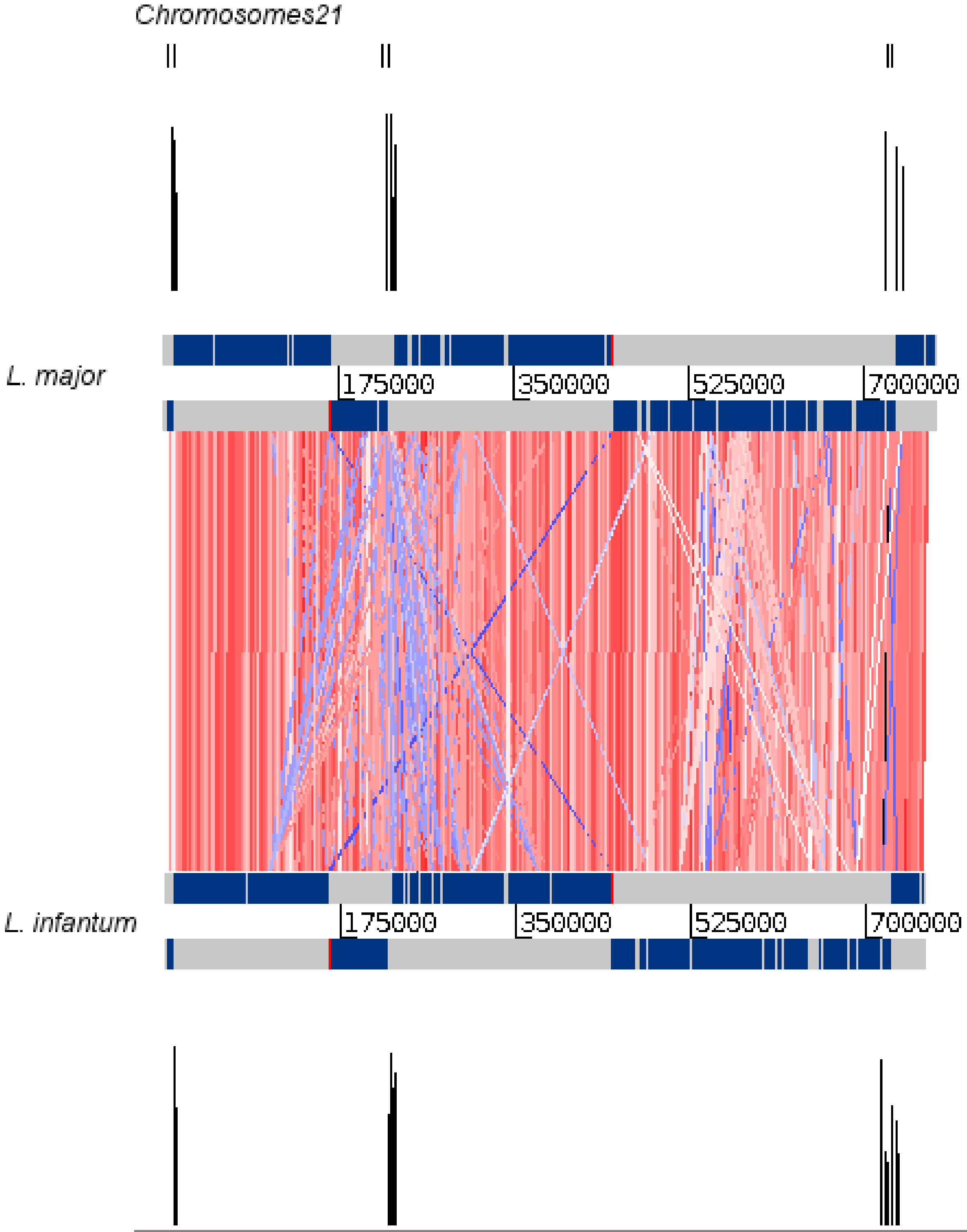

Chromosomes21
L. major
175000
350000
525000
700000
L. infantum
175000
350000
525000
700000

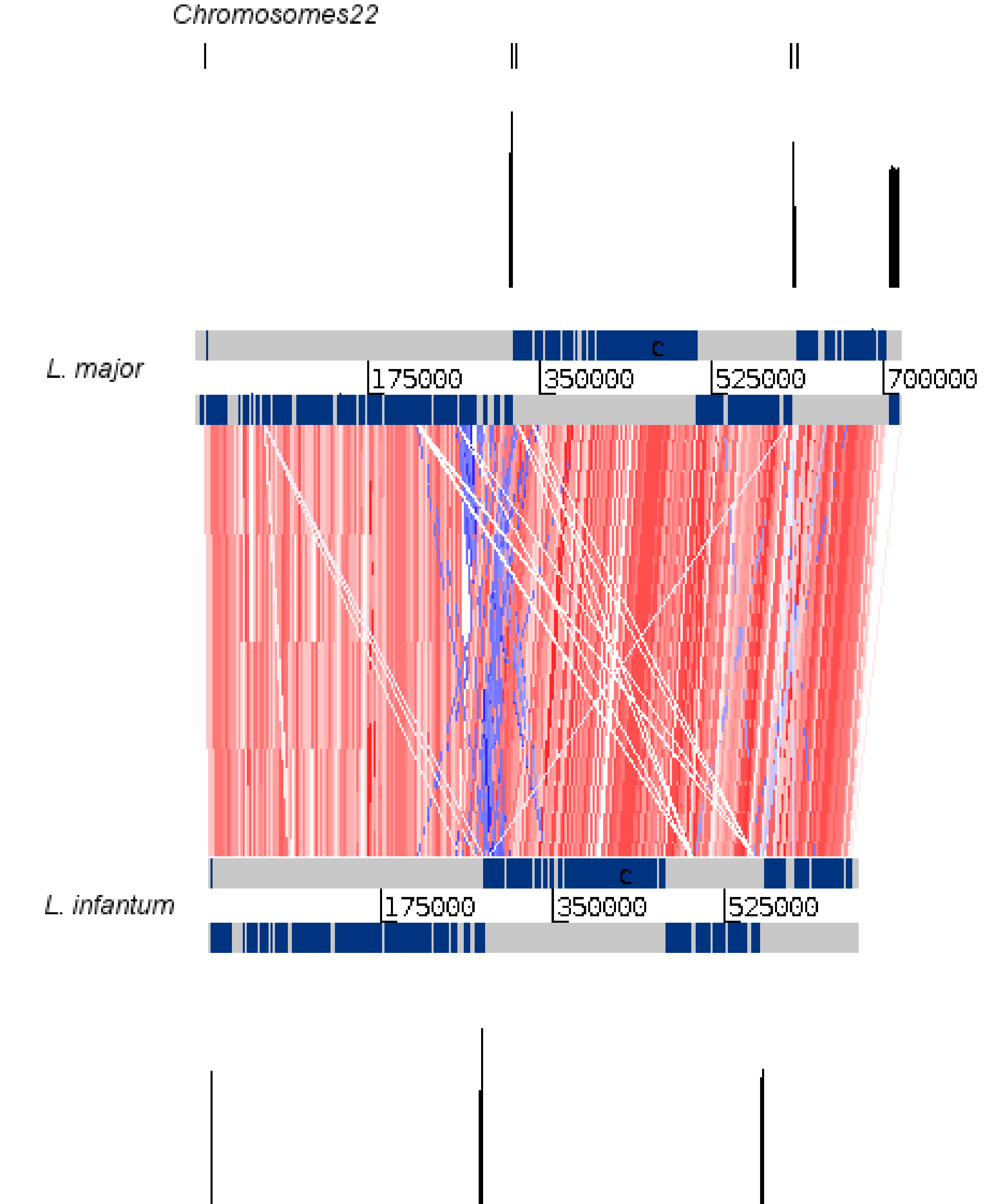
Chromosomes22
L. major
175000
350000
525000
700000
c
L. infantum
175000
350000
525000
c

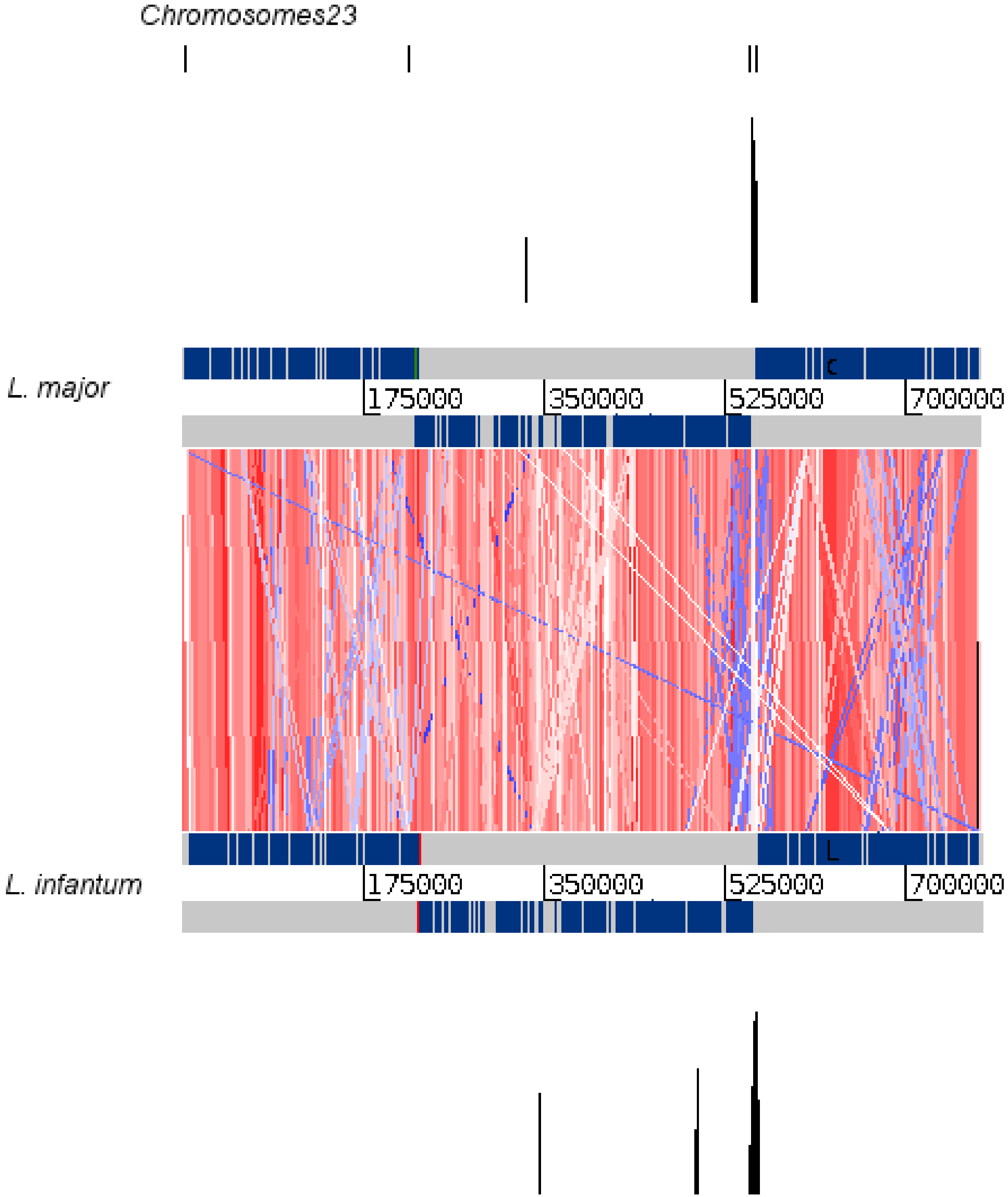
Chromosomes23
L. major
175000
350000
525000
700000
L. infantum
175000
350000
525000
700000

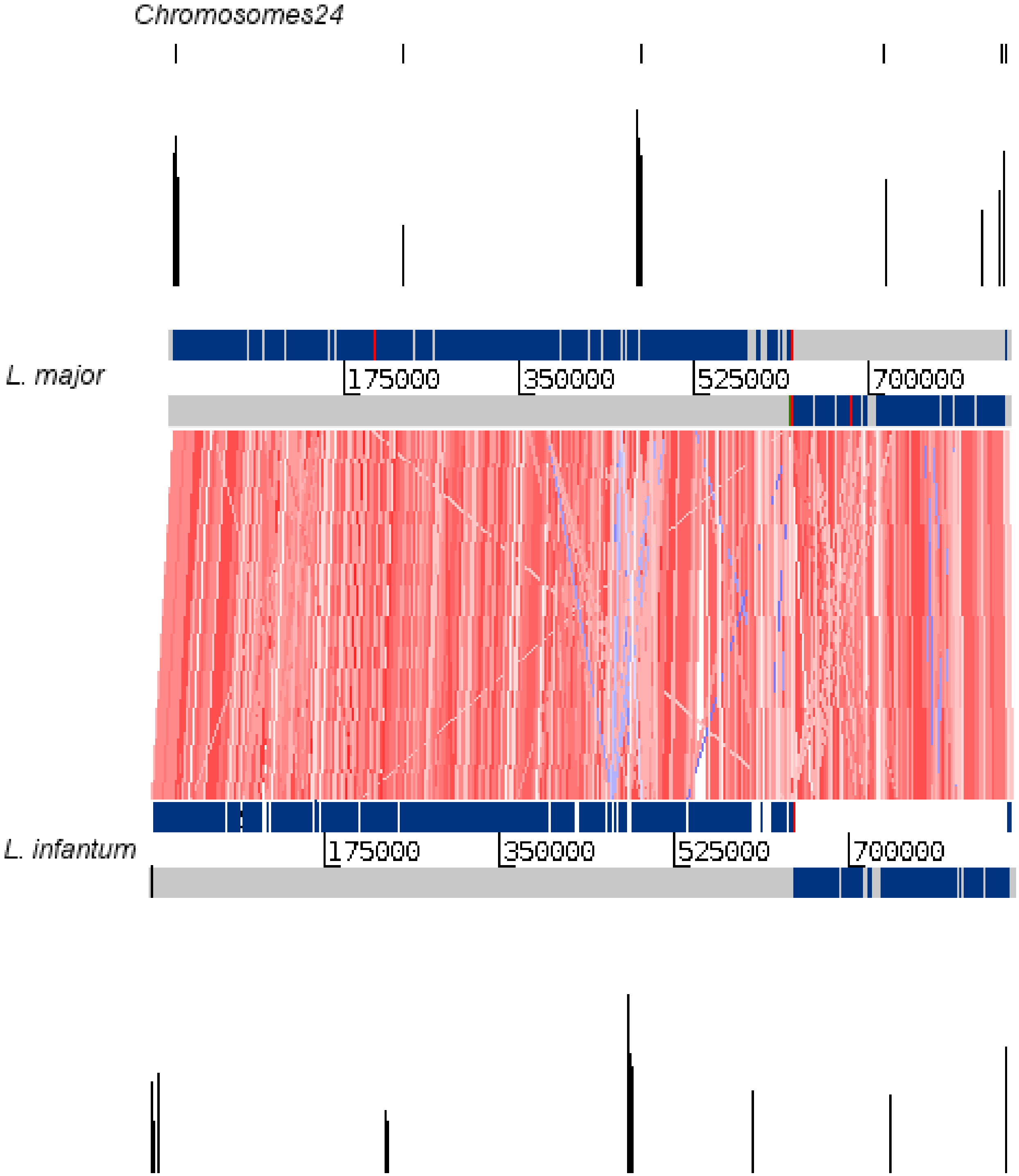
Chromosomes24
L. major
175000
350000
525000
700000
L. infantum
175000
350000
525000
700000

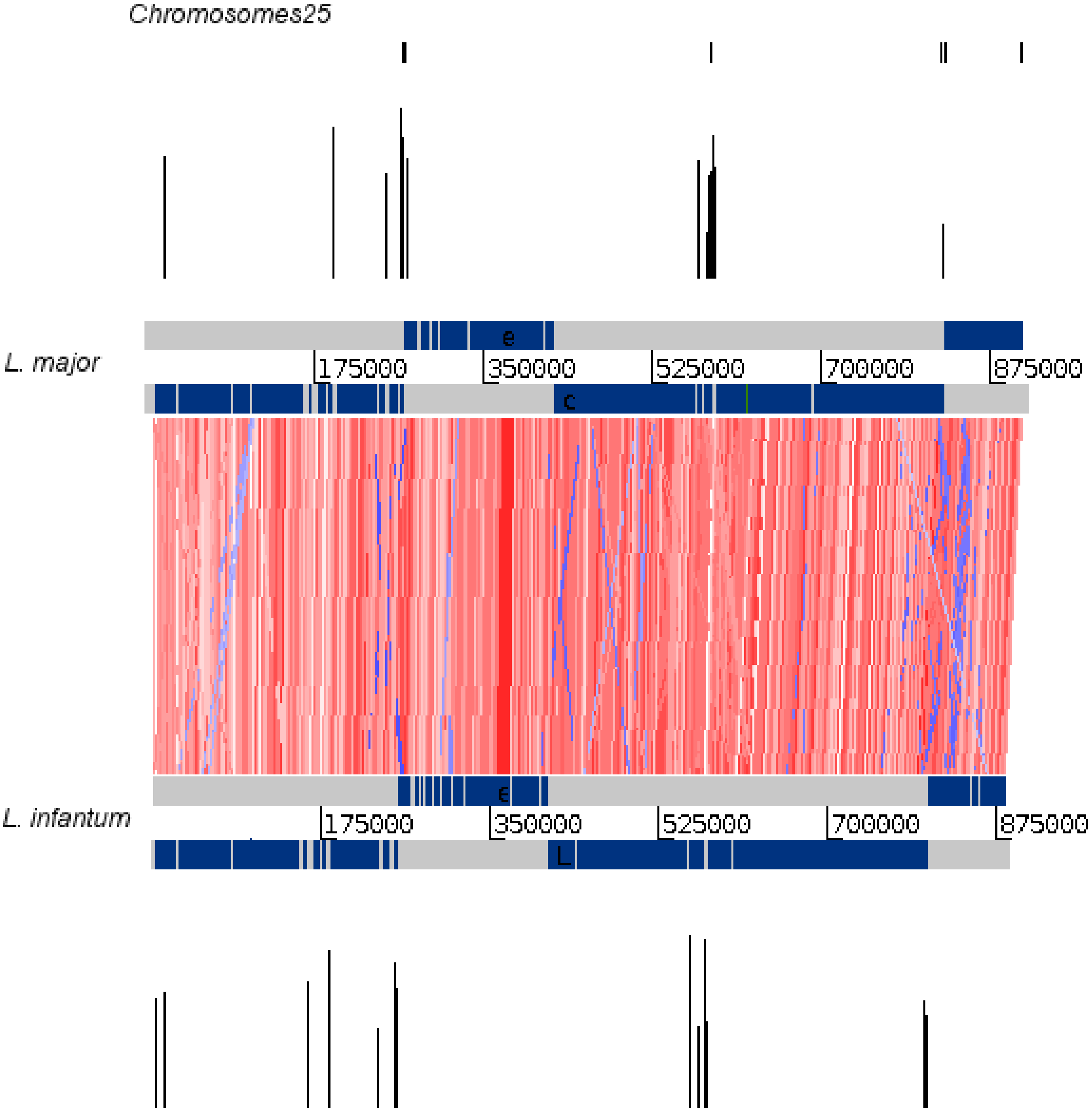
Chromosomes25
L. major
e
175000
350000
525000
700000
875000
c
e
L. infantum
175000
350000
525000
700000
875000
L

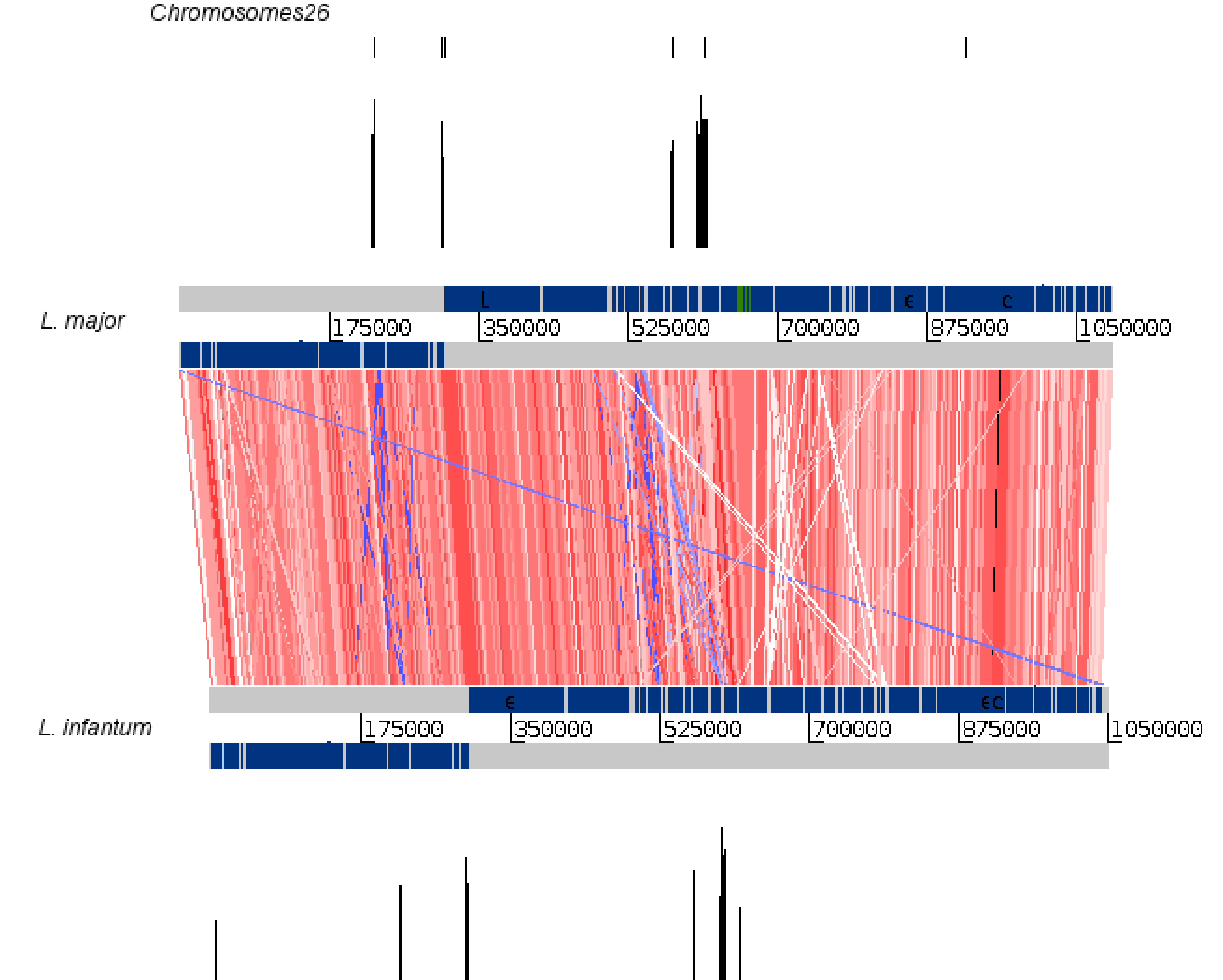

Chromosomes26
L. major
175000
350000
525000
700000
875000
1050000
L. infantum
175000
350000
525000
700000
875000
1050000

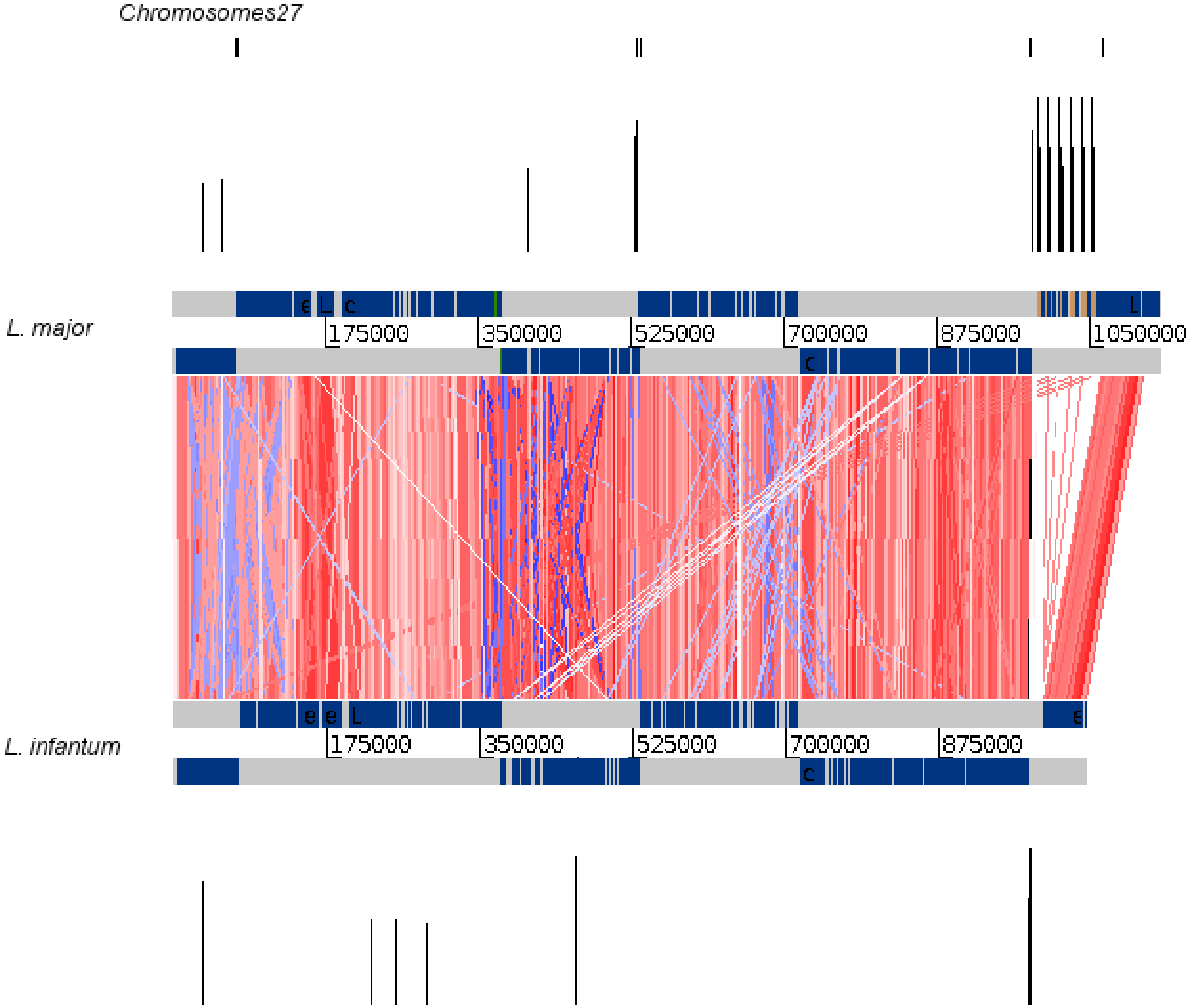

Chromosomes27
L. major
175000
350000
525000
700000
875000
1050000
L. infantum
175000
350000
525000
700000
875000

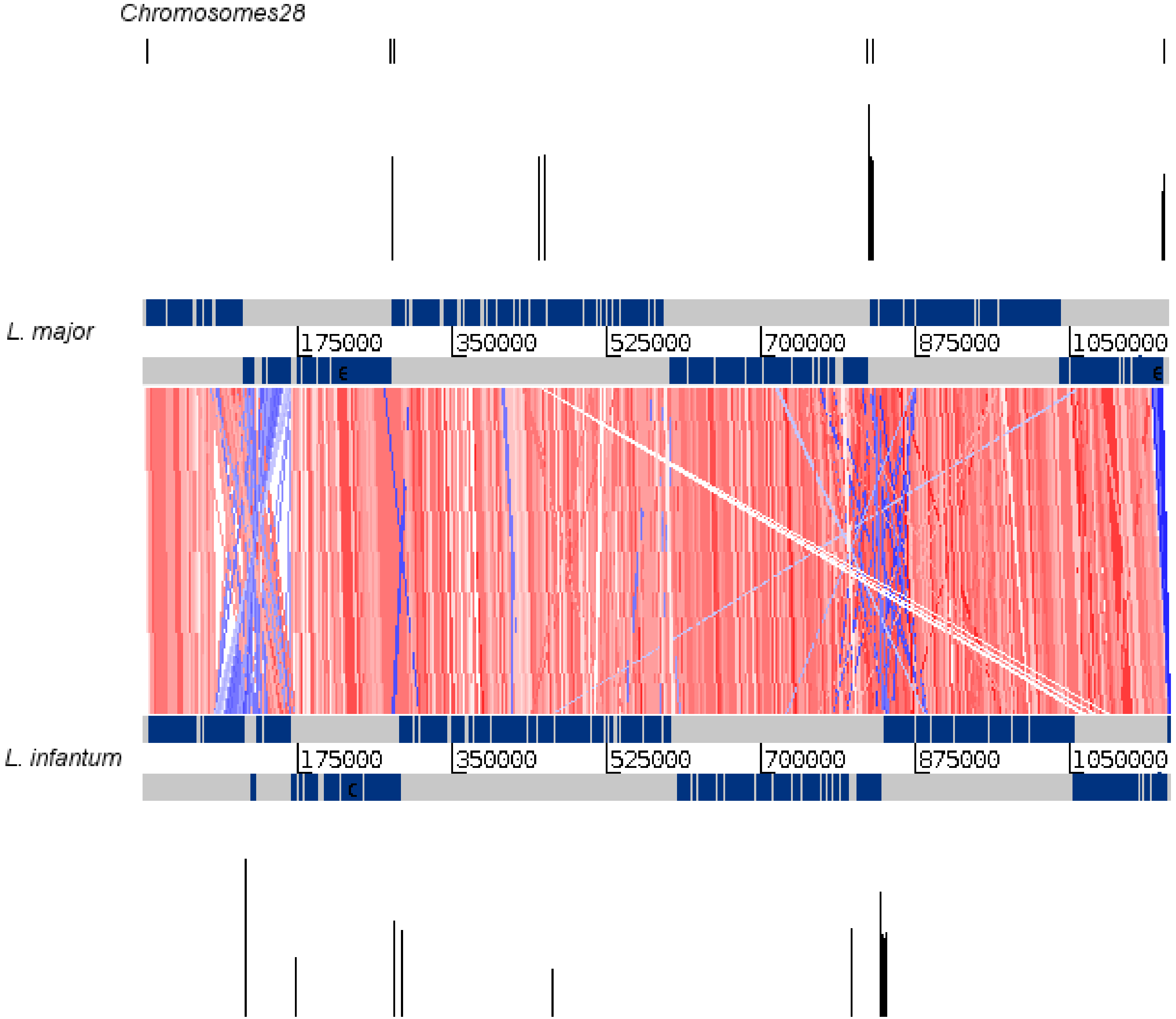

Chromosomes28
L. major
175000
350000
525000
700000
875000
1050000
L. infantum
175000
350000
525000
700000
875000
1050000

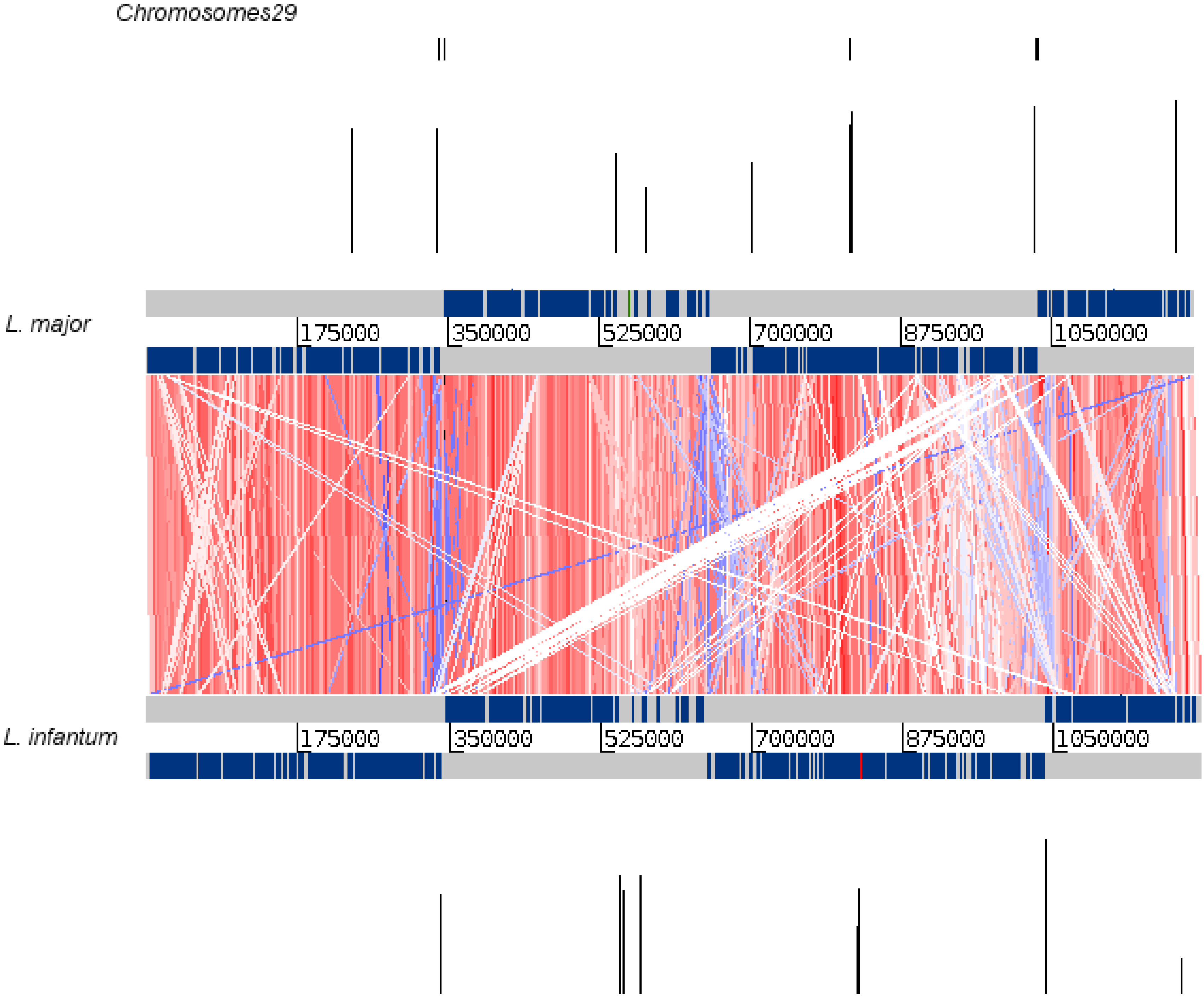
Chromosomes29
L. major
175000
350000
525000
700000
875000
1050000
L. infantum
175000
350000
525000
700000
875000
1050000

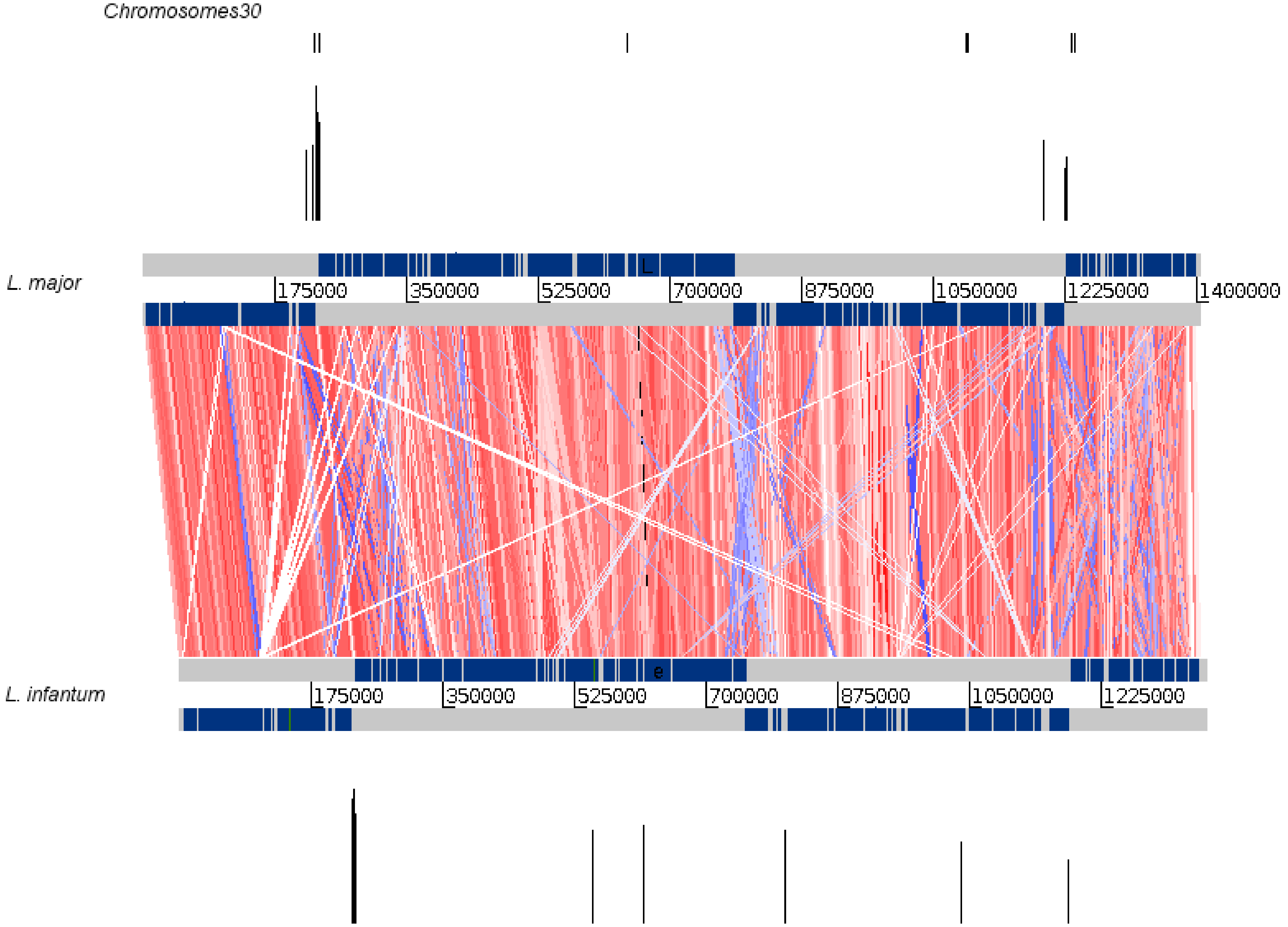

Chromosomes30
L. major
175000
350000
525000
700000
875000
1050000
1225000
1400000
L. infantum
175000
350000
525000
700000
875000
1050000
1225000

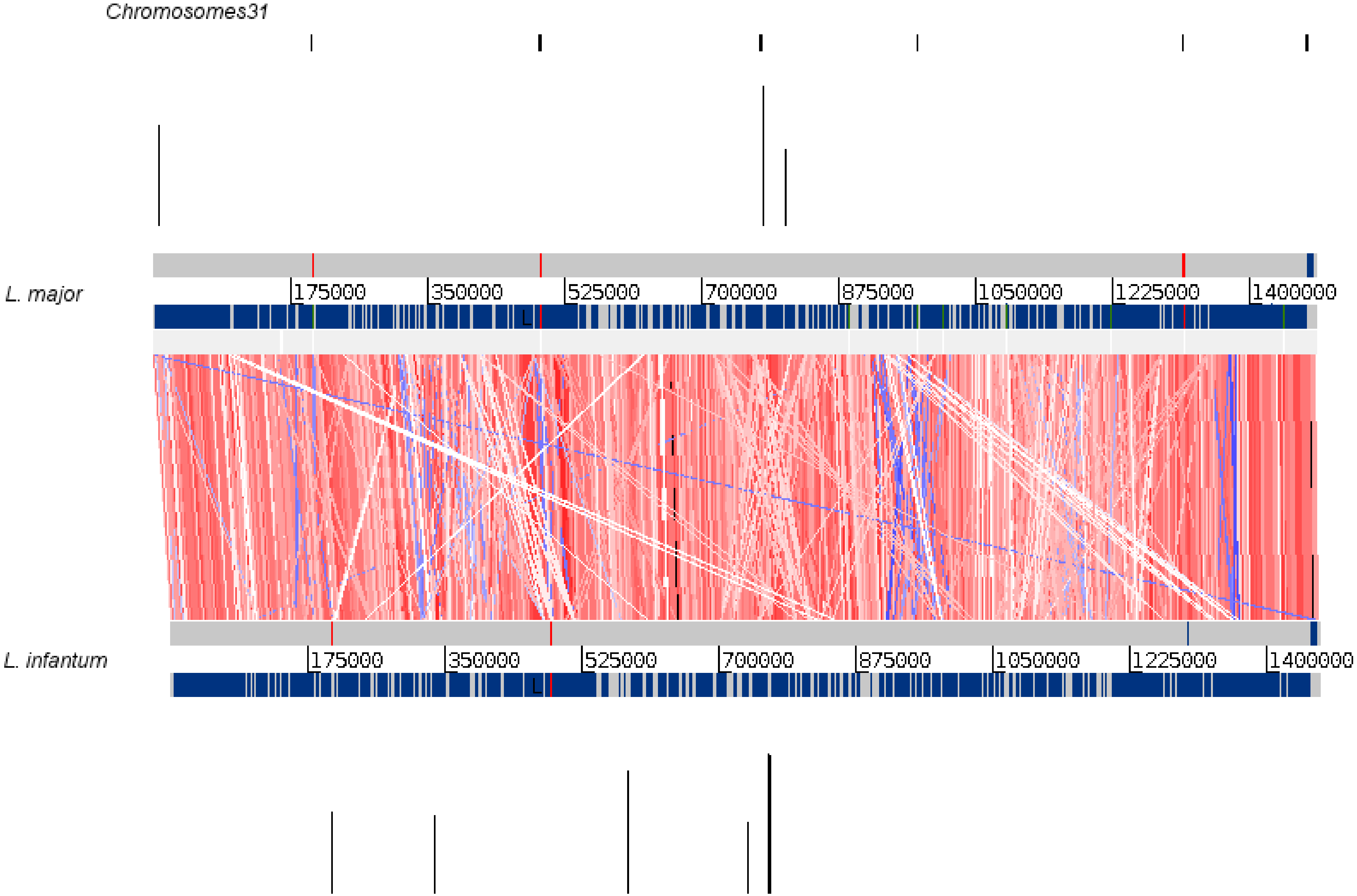
Chromosomes31
L. major
175000
350000
525000
700000
875000
1050000
1225000
1400000
L
L. infantum
175000
350000
525000
700000
875000
1050000
1225000
1400000
L

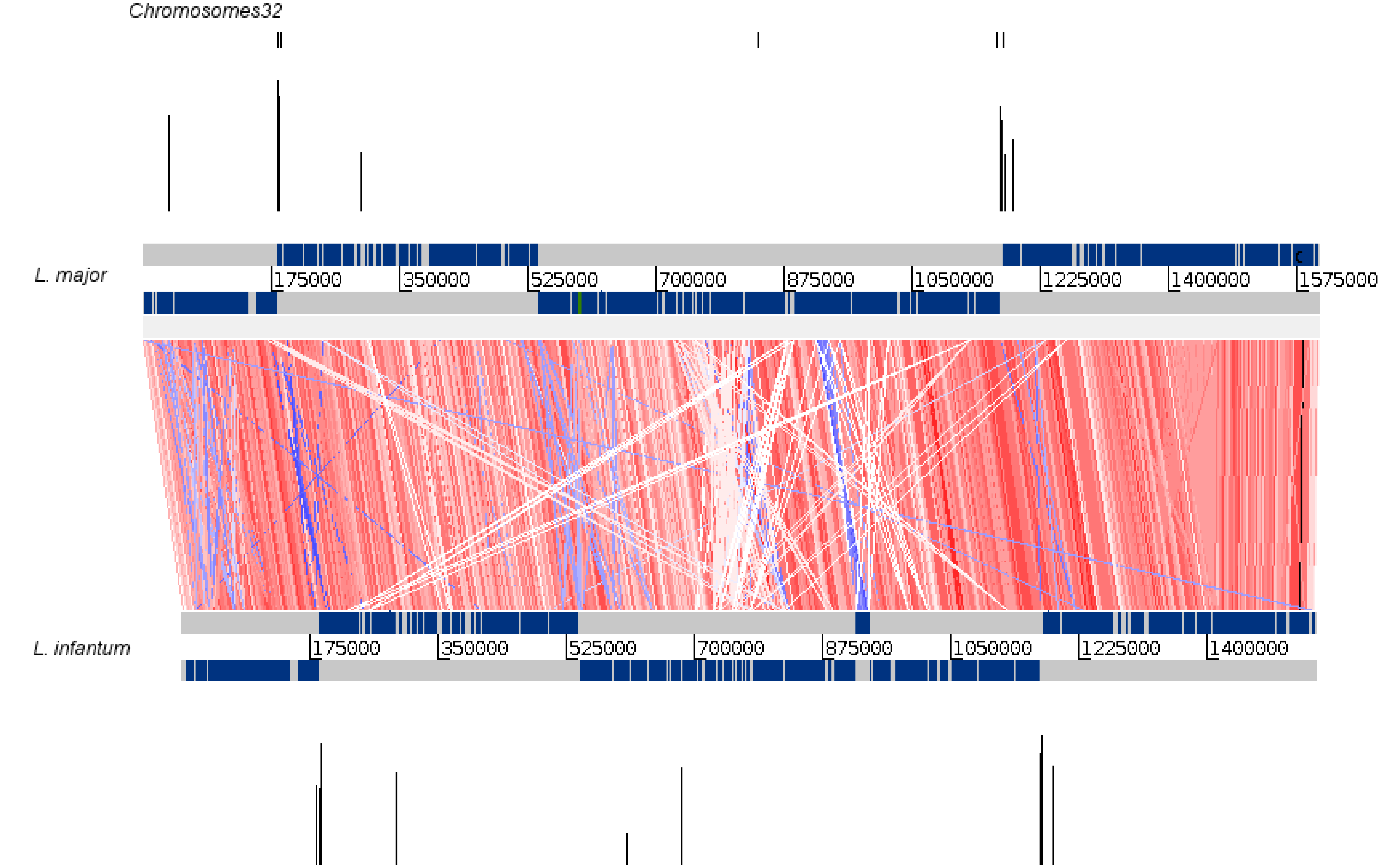
Chromosomes32
L. major
175000
350000
525000
700000
875000
1050000
1225000
1400000
1575000
L. infantum
175000
350000
525000
700000
875000
1050000
1225000
1400000

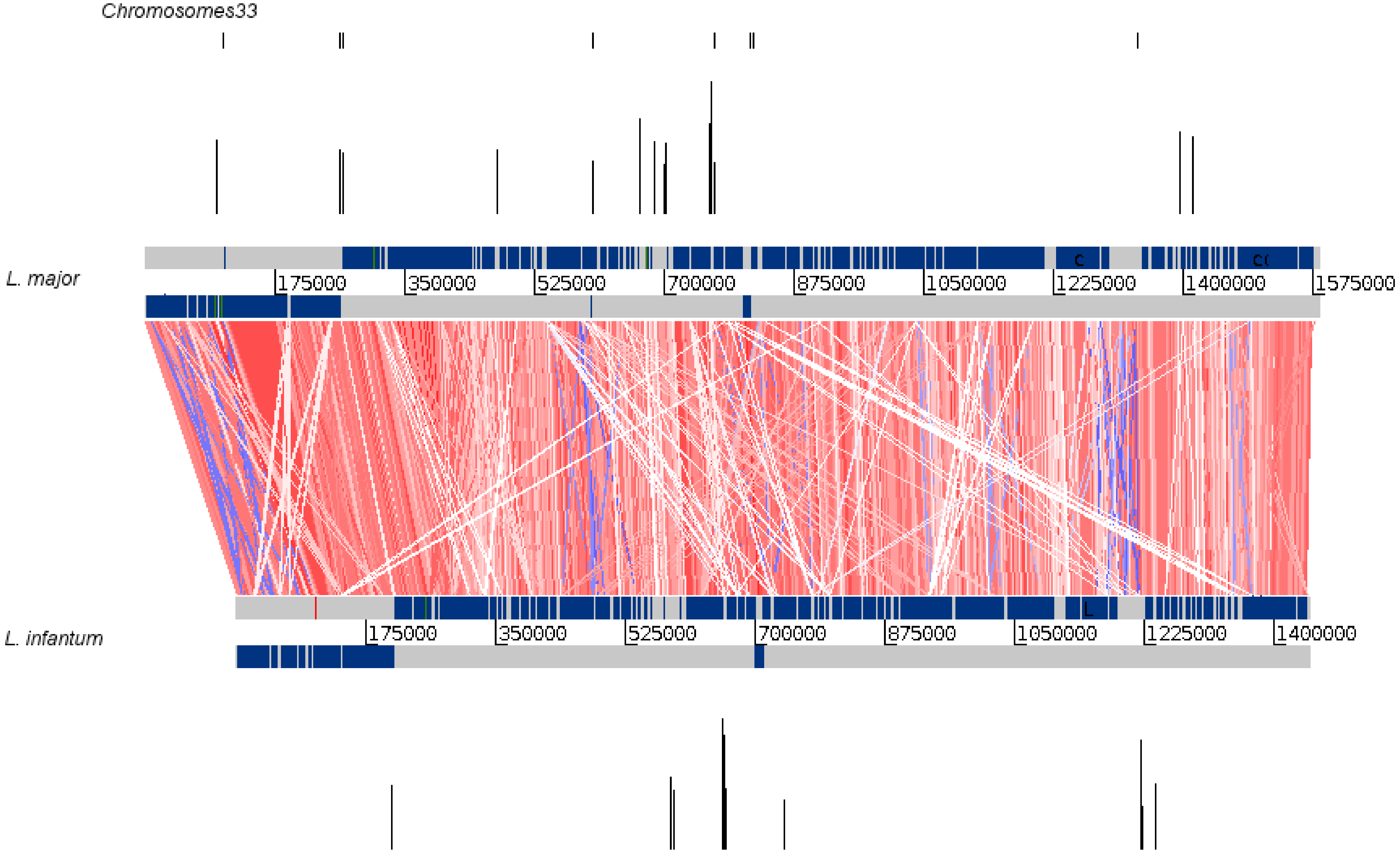

Chromosomes33
L. major
175000
350000
525000
700000
875000
1050000
1225000
1400000
1575000
L. infantum
175000
350000
525000
700000
875000
1050000
1225000
1400000

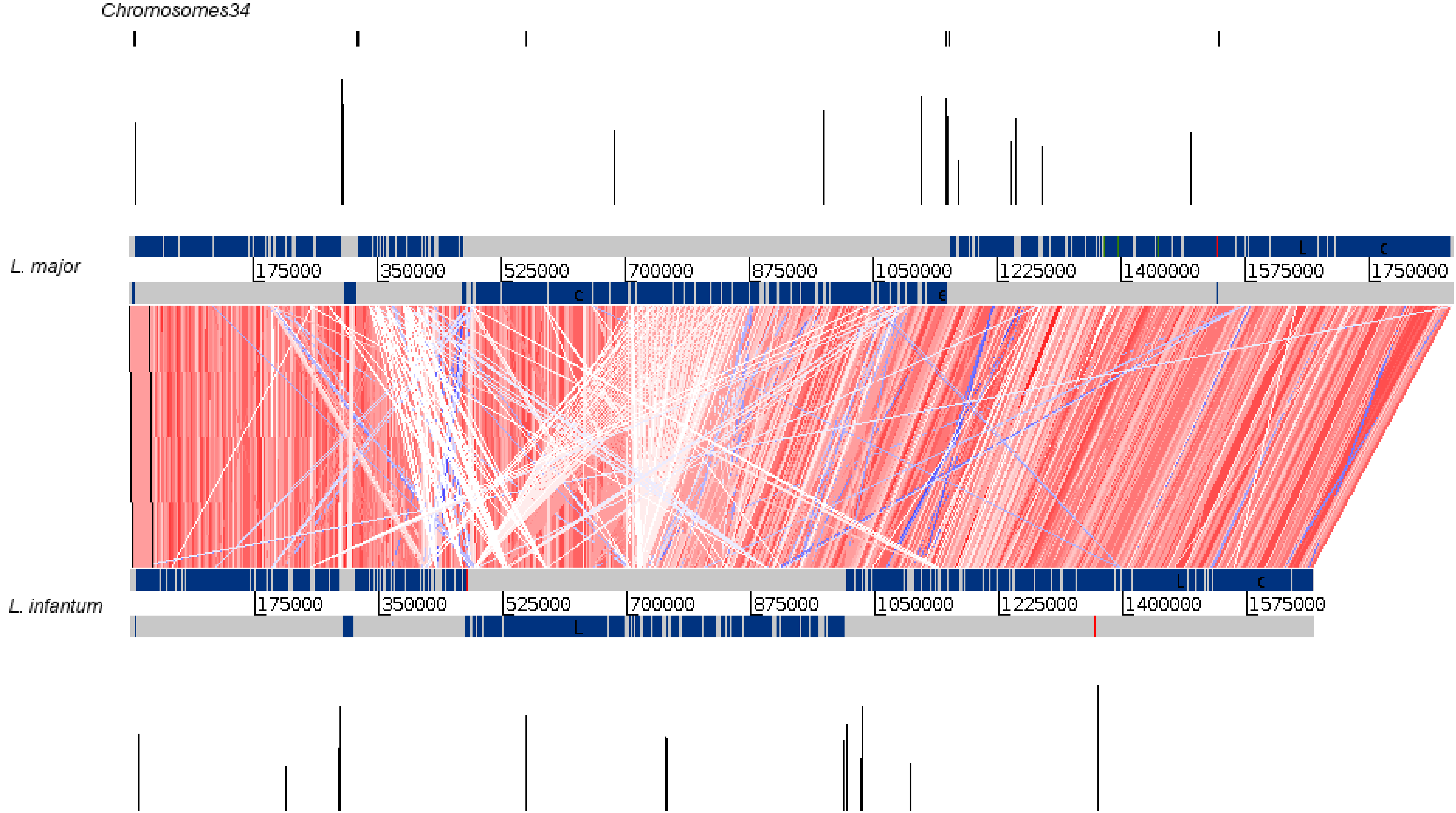

Chromosomes34
L. major
175000
350000
525000
700000
875000
1050000
1225000
1400000
1575000
1750000
L. infantum
175000
350000
525000
700000
875000
1050000
1225000
1400000
1575000

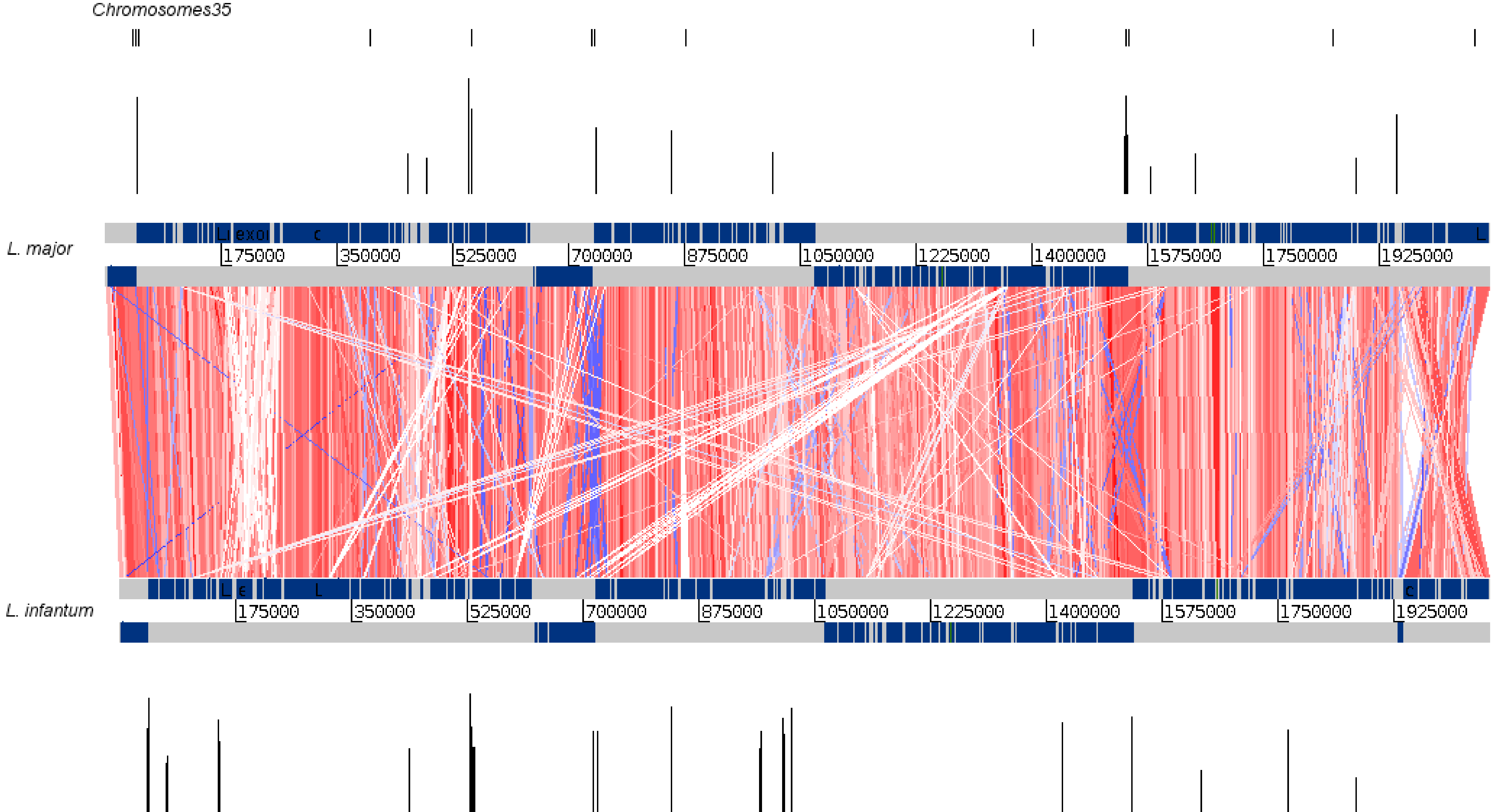
Chromosomes35
L. major
175000
350000
525000
700000
875000
1050000
1225000
1400000
1575000
1750000
1925000
L. infantum
175000
350000
525000
700000
875000
1050000
1225000
1400000
1575000
1750000
1925000

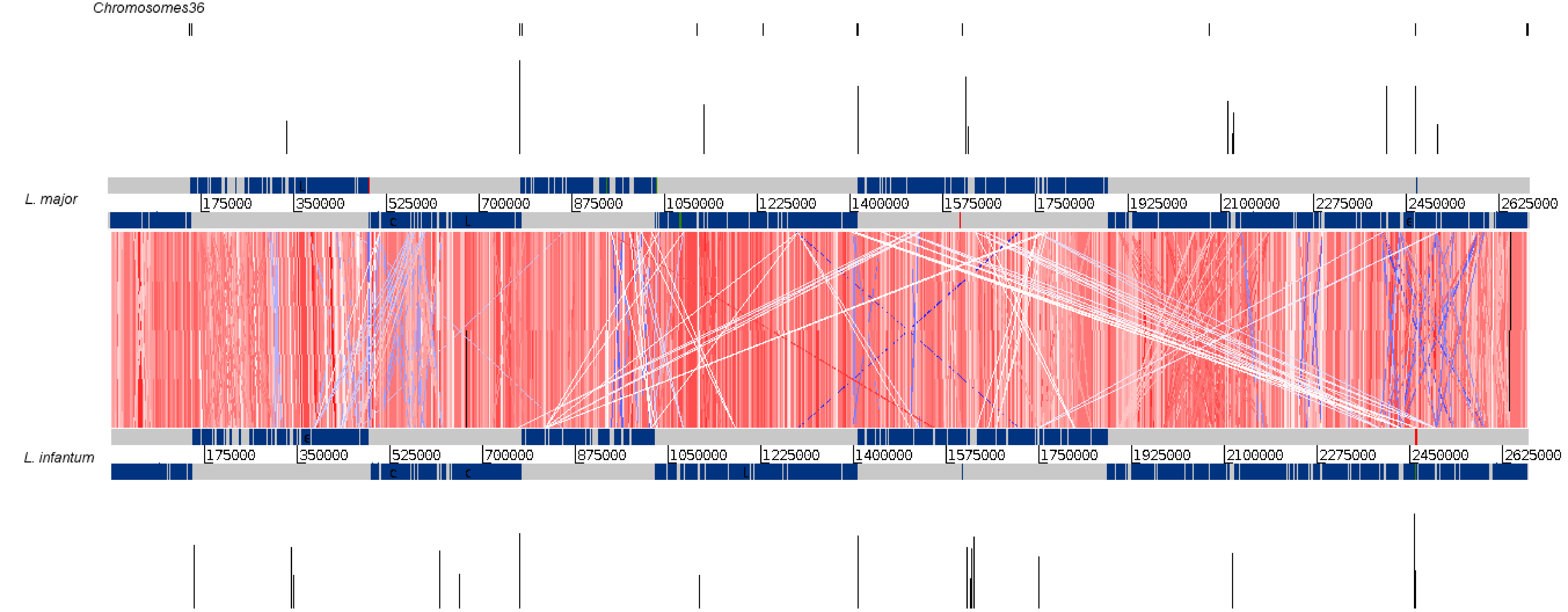
Chromosomes36
L. major
175000
350000
525000
700000
875000
1050000
1225000
1400000
1575000
1750000
1925000
2100000
2275000
2450000
2625000
L. infantum
175000
350000
525000
700000
875000
1050000
1225000
1400000
1575000
1750000
1925000
2100000
2275000
2450000
2625000
