# Supplementary material for: Genomic Analysis of Sequence-Dependent DNA Curvature in Leishmania
Source: PLoS One. 2013 Apr 30;8(4):e63068. doi: 10.1371/journal.pone.0063068 (PMC3639952; doi:10.1371/journal.pone.0063068)
Supplement: Table S1 — Genome intrinsic curvature in Tritryps. (PDF) [file pone.0063068.s008.pdf]

Supplementary Table 1. Genome intrinsic curvature

Organism	genome length (Mb)	G+C (%)	median IC (degrees/hel. turn)
L. major	32.9	59.7	2.63
L. infantum ***L. braziliensis*** ***L. mexicana***	32.3 ± 0.4	59.2 ± 1.0	2.67 ± 0.06
T. cruzi	32.5	50.3	3.36
T. brucei	26.2	46.4	3.52
E. coli	4.6	50.8	3.7
H. sapiens*	6.7	40.1	3.8

*The values correspond to the genome fragment analyzed (see Materials and Methods).
