# Supplementary material for: Genomic Analysis of Sequence-Dependent DNA Curvature in Leishmania
Source: PLoS One. 2013 Apr 30;8(4):e63068. doi: 10.1371/journal.pone.0063068 (PMC3639952; doi:10.1371/journal.pone.0063068)
Supplement: Table S2 — Chromosome intrinsic curvature in L. major. (PDF) [file pone.0063068.s009.pdf]

Supplementary Table 2

Chromosome intrinsic curvature in *L major*

Number	Length (bp)	G+C (%)	Median IC	CDS (%)
1	268984	63,08	2,37	52,61
2	355714	62,98	2,48	51,07
3	384518	62,99	2,41	61,27
4	472856	61,74	2,43	54,99
5	465823	61,93	2,45	54,58
6	516874	62,28	2,44	56,46
7	596348	61,40	2,49	53,33
8	574972	62,22	2,46	43,72
9	573441	61,09	2,50	53,66
10	570864	59,80	2,55	46,67
11	582575	59,39	2,66	45,80
12	675347	61,61	2,58	43,72
13	654604	60,53	2,54	53,91
14	622648	59,92	2,59	48,58
15	629514	61,69	2,45	55,42
16	714659	61,03	2,50	54,32
17	684831	60,86	2,52	52,01
18	739751	58,98	2,63	43,16
19	702212	59,64	2,63	45,76
20	742551	59,89	2,60	49,96
21	772974	60,42	2,54	51,25
22	716608	59,20	2,64	48,29
23	772567	59,50	2,63	46,22
24	840950	60,41	2,57	54,09
25	912849	60,06	2,56	51,49
26	1091579	60,39	2,57	52,39
27	1130447	58,80	2,69	46,30
28	1160128	59,22	2,67	47,70
29	1212674	58,71	2,72	44,56
30	1403454	58,81	2,72	45,17
31	1484336	59,03	2,69	43,67
32	1604650	58,81	2,72	46,59
33	1583673	59,07	2,69	45,37
34	1866754	59,32	2,68	46,24
35	2090491	58,23	2,75	47,00
36	2682183	58,13	2,78	48,20
